# Seroprevalence of Rift Valley fever virus and factors associated with exposure in south-central Uganda

**DOI:** 10.1186/s12879-026-13923-1

**Published:** 2026-07-07

**Authors:** Josephine Nalwadda, Victor Ssempijja, Steven J. Reynolds, Gerald Katushabe, Jonah Omooja, Evan A. Mihalakakos, Deepashri Rao, Kyle Rosenke, Mary K. Grabowski, Ronald M. Galiwango, Robert Ssekubugu, David Meya, Denis K. Byarugaba, Heinz Feldmann, David W. Hawman

**Affiliations:** 1 Laboratory of Virology, NIAID/NIH International Centers for Excellence in Research, Uganda Virus Research Institute, Entebbe, Uganda; 2 Clinical Epidemiology Unit, School of Medicine, College of Health Sciences, Makerere University, Kampala, Uganda; 3 Clinical Monitoring Research Program Directorate, Frederick National Laboratory for Cancer Research, Frederick, MD USA; 4 Division of Infectious Diseases, Johns Hopkins School of Medicine, Baltimore, MD USA; 5 Laboratory of Virology, Division of Intramural Research, NIAID/NIH, Rocky Mountain Laboratories, Hamilton, MT USA; 6 Department of Pathology, Johns Hopkins School of Medicine, Baltimore, MD USA; 7 Laboratory of Immunoregulation, NIAID/NIH, Bethesda, MD USA; 8 College of Veterinary Medicine, Makerere University, Kampala, Uganda; 9 Department of Internal Medicine, School of Medicine, College of Health Sciences, Makerere University, Kampala, Uganda; 10 Rakai Health Sciences Program, Kalisizo, Uganda; 11 Laboratory of Viral Zoonoses, Integrated Research Facility, Division of Intramural Research, NIAID/NIH, Frederick, MD USA

**Keywords:** Rift Valley fever virus, Seroprevalence, Uganda, Zoonotic infection, Arbovirus, Mosquito-borne disease

## Abstract

**Background:**

Rift Valley fever (RVF) poses a recurring public health and economic challenge in Uganda’s cattle corridor. It is a mosquito-borne zoonotic disease caused by the Rift Valley fever virus (RVFV), with high livestock density, favorable rainfall patterns, and close human–animal interaction facilitating viral transmission and disease outbreaks. Although RVF outbreaks have occurred, data on human exposure in south-central Uganda remain limited.

**Methods:**

This cross-sectional study assessed the seroprevalence and associated factors of RVFV exposure among selected communities in the greater Masaka-Rakai region of south-central Uganda. Archived human sera (*n* = 1,199) retrospectively collected between June 2018 and October 2020 from the population-based Rakai Community Cohort Study were analyzed using both indirect in-house and commercial ID Screen^®^ RVF competition enzyme-linked immunosorbent assays (ELISAs) to detect RVFV IgG antibodies. Household census and socio-demographic data were linked to serological results, and logistic regression was performed to identify factors associated with seropositivity.

**Results:**

RVFV IgG antibodies were detected in 86 of 1,199 (7.8%) individuals. In multivariable analysis, RVFV seropositivity was significantly associated with residence in agrarian communities and older age groups (25–34 and ≥ 35 years), while pig ownership was associated with lower risks of exposure.

**Conclusion:**

These findings suggest RVFV human exposure in south-central Uganda and emphasize the need for strengthened surveillance, targeted prevention, and One Health interventions to protect high-risk groups.

**Supplementary Information:**

The online version contains supplementary material available at https://doi.org/10.1186/s12879-026-13923-1.

## Background

Rift Valley fever (RVF) is a mosquito-borne zoonosis that poses a major public health and economic threat in Africa and beyond. The disease is caused by the Rift Valley fever virus (RVFV) and can progress to a viral hemorrhagic fever in humans [1]. Infected ruminants can experience mass abortions with mortality rates up to 100% [2, 3]. In humans, infections can be asymptomatic or have symptoms ranging from mild flu-like illness to severe hemorrhagic manifestations [4–6] with fatality rates of up to 50% in severe cases [7]. Comorbidities such as diabetes, chronic kidney disease, and HIV/AIDS further increase severe disease outcome [8]. Following infection, symptoms typically emerge within 2–6 days of exposure [9, 10]. The host mounts short- and long-term antibody responses. Immunoglobulin M (IgM) emerges within the first week and wanes after 2–3 months, marking recent infection, while IgG develops later and persists for years, indicating past exposure and long-term immunity [2]. Understanding the full spectrum of RVFV infections, including asymptomatic cases, is crucial for accurately estimating disease burden, as undiagnosed infections may lead to underestimation of population exposure, hindering effective surveillance, timely diagnosis, and control efforts [11].

Major RVF outbreaks have caused widespread morbidity and mortality across decades. The 2000 outbreak among humans on the Arabian Peninsula recorded a 14% case fatality rate (CFR) [12, 13]. This was the first occurrence outside of Africa, raising concerns about the wider spread of RVFV. In sub-Saharan Africa, RVFV is endemic in several countries, including Uganda, Kenya, Tanzania, Sudan, Mauritania, South Africa, and Senegal, where outbreaks often follow periods of heavy rainfall or flooding [14, 15]. Uganda’s cattle corridor, stretching diagonally from the northeastern to southwestern parts of the country, is characterized by extensive pastoral livestock systems, high livestock mobility, and frequent human–livestock interaction [16]. Environmental conditions such as high rainfall and proximity to water bodies with seasonal floods create favorable breeding sites for mosquito vectors and support livestock farming. Consequently, the region experiences recurrent RVFV outbreaks approximately every 2–4 years due to the favorable ecological conditions and proximity to endemic regions [17, 18]. Between 2016 and 2018, outbreaks across 10 districts resulted in 16 human cases and 7 deaths (CFR = 45%), all among laboratory-confirmed severe cases, consistent with reports of high mortality in severe RVF cases [19].

Human infection occurs through both mosquito bites and direct contact with infected animal materials (blood, tissues, carcasses, or aborted fetuses) during activities such as slaughter, birthing assistance, or veterinary procedures, with occupational exposure to livestock often representing the dominant risk factor [20]. Risk is heightened by keeping livestock near homes, ineffective mosquito control, and occupational exposure in farming, veterinary work, or slaughterhouse operations [21, 22]. Environmental drivers, especially heavy rainfall and flooding, create ideal breeding conditions for mosquitoes and can trigger outbreaks [23]. In south-central Uganda, episodic flooding is common, creating temporary bodies of water and expansive wetland conditions that sustain mosquito amplification cycles, thereby increasing the likelihood of RVFV circulation. In Uganda, transmission peaks during the March–May and the September–November rainy seasons [24]. Although livestock vaccines exist, there is no licensed human vaccine [25, 26] or specific antiviral treatment for RVF in humans [10]. Medical care remains supportive, and control focuses on mosquito prevention, livestock vaccination, and public health education [27, 28].

Although Uganda’s national One Health strategy emphasizes prevention of zoonotic diseases, responses remain largely reactive, triggered by outbreaks rather than proactive surveillance and early risk factor identification. The Food and Agriculture Organization (FAO) has forecasted high RVF risk across East Africa, recommending enhanced awareness and preparedness efforts at national, subnational, and community levels [29]. Further, metadata from 2008 to 2022 reveals geographic expansion of RVF clusters into previously unaffected regions, about 35% occurring in regions without prior RVFV activity, including south-central Uganda [30]. Here we retrospectively examined the seroprevalence of RVFV and factors associated with exposure among individuals in selected communities in south-central Uganda. This region lies within Uganda’s cattle corridor, extending from the western parts of Lake Victoria to the Mutukula border with Tanzania in the south. It is characterized by a tropical climate with distinct wet (March–May; September–November) and dry seasons, which influence livestock farming and RVFV epidemiology [18, 24, 31].

## Methods

### Study design, study setting, and study population

This was a cross-sectional study conducted using retrospectively collected data and serum samples from the Rakai Community Cohort Study (RCCS) participants [32, 33], of the survey round that was conducted between June 2018 and October 2020. We used demographic, behavioral, and disease surveillance data along with serum samples collected from participants of the RCCS in the greater Masaka–Rakai region. The RCCS is an ongoing longitudinal, population-based cohort study conducted by the Rakai Health Sciences Program (RHSP) since 1994. Approximately every 18 months, RCCS conducts a survey round that starts with a census of all permanent and transient residents across 21 agrarian, 16 trading, and 4 fishing communities, which are grouped according to the predominant economic activity [34]. Following the census, all eligible residents aged ≥ 15 years are invited to participate. Consenting individuals are then interviewed using structured, confidential tools and provide blood samples, GPS coordinates, and sociodemographic and health data. Each survey round recruits ~ 20,000 participants comprising new and follow-up residents from these communities. The sample size, community types, and demographics of this cohort were previously published [35].

### Study variables

The primary outcome was serological evidence of exposure to RVFV, determined through antibody detection using enzyme-linked immunosorbent assay (ELISA). Potential confounders included socio-demographic characteristics (age, gender, and highest level of education attained) and household or community-level factors, including livestock ownership (cattle, goats, and pigs), bed net use, availability of cultivable land, occupation, and community type (agrarian, trading, or fishing).

### Serological testing for Rift Valley fever virus

A serial testing algorithm was applied for RVFV IgG detection. Samples were classified as seropositive if they were positive in both the commercial ID Screen^®^ assay and the in-house ELISA. Agreement between the two assays was assessed by calculating percent agreement and Cohen’s kappa (κ) statistic, with percent agreement defined as the proportion of samples with concordant results. Analyses were performed using Stata/MP version 18 [36].

### Indirect in-house ELISA

Serum samples were heat-inactivated at 56 °C for 1 h and screened by an in-house ELISA, with samples run in duplicate. Nunc Maxisorp 96-well plates were coated overnight at 4 °C with RVFV nucleoprotein antigen (strain ZH548, Native Antigen REC31640-500) diluted at 1:12,800 in PBS. The following morning, the excess antigen was removed, plates were blocked with 0.1% milk in PBS-Tween for 1 h at room temperature (RT). Plates were washed 3x with 250µL of PBS-Tween. Serum samples diluted 1:400 in 0.1% milk were added to the plates (100µL per well) in duplicate. Blank wells, known RVFV IgG-positive human sera, and negative pooled human sera were added in duplicate to each plate as controls. Plates were incubated at RT for 1 h, then washed 3x with 250µL of PBS–Tween. A secondary human antibody biotin conjugate (Goat Anti-Human IgG-BIOT, SouthernBiotech, Cat. No.: 2040-08), diluted 1:4000 in PBS-Tween, was added (100µL per well), followed by incubation at RT for 1 h. Plates were washed 3x with 250µL of PBS-Tween. Streptavidin–HRP conjugate (SouthernBiotech, Cat. No.: 7105-05), diluted 1:4000 in PBS, was then added (100µL per well) and incubated at RT for 30 min. Plates were subsequently washed 6x with 250µL of PBS-Tween. ABTS^®^ 2-Component Microwell Peroxidase Substrate containing 2, 2’-azino-di (3-ethylbenzthiazoline-6-sulfonate, SeraCare, Material No. 5120-0032) was added at a 1:1 dilution (100µL per well), and plates were incubated in the dark for 30 min. Optical density (OD) was measured and read spectrophotometrically at 405 nm using a SmartReader™ 96 Absorbance Plate Reader (model 9600), and values were averaged across duplicate wells. The IgG cut-off value was defined as the mean + 2 standard deviations of the negative control serum pool; samples above this threshold were classified as positive. The assay was optimized before use by determining optimal antigen and antibody dilutions and assay performance was monitored using known negative and positive controls to ensure assay performance. Agreement between the assays was assessed using percentage agreement and Cohen’s kappa statistic to assess agreement beyond chance.

### ID screen^®^ RVF competition ELISA

Samples were also run for secondary assay confirmation by the ID Screen^®^ RVF Competition Multi-species ELISA (Innovative Diagnostics) for RVFV nucleoprotein IgG antibodies, following the manufacturer’s instructions. This assay is based on a competitive binding principle, in which antibodies present in the sample compete with labeled antibodies for binding to the antigen; therefore, lower signal intensity corresponds to higher antibody levels. This kit uses recombinant RVFV nucleoprotein as the coating antigen and has demonstrated a sensitivity of 98% and a specificity of 100% [37]. Results were expressed as competition percentage (S/N%), calculated as: S/N% = (OD_S_ of the sample / OD_NC_ of the negative control) × 100. Samples with S/N% ≤ 40% were classified as positive, while those with S/N% > 50% were considered negative, according to the kit instructions. The test validity was assessed in each plate run using kit-provided controls. In addition, known RVFV IgG-positive human serum was used as a positive control, while pooled normal human serum (Innovative Research) and blank wells were used as negative controls. All samples and controls were run in duplicate on each plate.

### Statistical analysis

Frequencies and percentages were used to describe the distributions of potential confounding variables, while chi-square or Fisher’s exact tests were used to test differences in distributions between the community types. Categorical variables were described using frequencies and proportions, and relevant results were presented in tables to show distribution patterns across demographic groups and visualize community types. Seroprevalence with 95% confidence intervals (CIs) was estimated for each community type from the proportion of the RVFV-positive samples, and overall seroprevalence was computed from the population-weighted proportion of RVFV-positive samples. Significant association between potential confounders and the prevalence level of RVFV was assessed using odds ratios with corresponding 95% confidence intervals (CIs) that were computed from bivariable and multivariable logistic regression analysis. Variables with *p* < 0.20 in the bivariable analysis were included in the multivariable model; significance was set at *p* < 0.05. Interaction was assessed by generating interaction terms with significant variables and using the chunk test to identify any significant interactions. Unadjusted and adjusted odds ratios (ORs) with 95% CI were reported; model fit was analyzed using the Hosmer-Lemeshow test. Dual seropositivity for RVFV and CCHFV was determined by linking individual-level serological results from the current RVFV analysis with CCHFV IgG results from the same cohort [35] and identifying participants who were positive for both infections. To determine whether exposure risk was driven primarily by direct animal contact, the broader ecological environment, or both, livestock ownership and community type interaction effect to the outcome was assessed; subgroup-specific adjusted odds ratios were reported. Data was analyzed using Stata/MP version 18 (StataCorp LLC, College Station, TX, USA) [36].

## Results

### Participant demographics

The study included 1,199 archived serum samples collected across 21 agrarian (*n* = 400), 16 trading (*n* = 399), and 4 fishing (*n* = 400) communities. Demographic characteristics are presented in Supplemental Table 1. Participant characteristics varied significantly by community type (*p* < 0.05), reflecting heterogeneity in potential risk factors for RVFV exposure. Females predominated in agrarian and trading communities, while males were more common in fishing areas. Outdoor or animal-related occupations were frequent, particularly in agrarian and fishing communities. Livestock ownership and land cultivation were highest in agrarian communities and lowest in fishing communities. As RVFV is a mosquito-borne virus, we also evaluated bed net usage. Bed net use was generally high but slightly lower in fishing areas (Supplemental Table 1).

### Seroprevalence of RVFV by community type

Among the 1,199 samples analyzed, RVFV seroprevalence was 7.8% (95% CI: 1.12–16.57). Agreement between the in-house ELISA and the commercial ID Screen^®^ assay was high, at 96.8%, with a Cohen’s kappa (κ) of 0.812, indicating almost perfect agreement between the two tests. The highest seropositivity was observed in agrarian communities (10.5%, 95% CI: 7.85–13.91), followed by fishing (6%, 95% CI: 4.05–8.80) and trading (5%, 95% CI: 3.25–7.64) communities (Table 1). The spatial distribution of community seropositivity and GPS coordinates is shown in Fig. 1. Overall, seroprevalence differed significantly across the three community types (*p* = 0.006). Trading and fishing communities had lower and statistically similar seroprevalence compared to agrarian communities (*p* = 0.541). Since their serostatus did not differ significantly, they were pooled to increase statistical power for subsequent variable-specific analyses (Table 2). In the pooled comparison, agrarian communities had a significantly higher seroprevalence (10.5%) than the combined trading/fishing communities (5.5%, *p* = 0.002).

**Table 1:** Seroprevalence of RVFV across community types

	Community type			Overall n (%)
Exposure variable	Agrarian n (%)	Trading n (%)	Fishing n (%)	
**RVFV status**				
Negative	358(89.5)	379(95.0)	376(94.0)	1113(92.2)
Positive	42(10.5)	20(5.0)	24(6.0)	86(7.8)
**Chi-squared** ***p*-values**				
Agrarian vs Trading vs Fishing	0.006			
Trading vs Fishing	-	0.541		

**Table 2:** Pooled comparison of RVFV seroprevalence

Pooled comparison		
Exposure variable	Agrarian *n* (%)	Trading or Fishing *n* (%)
**RVFV status**		
Negative	358(89.5)	755(94.5)
Positive	42(10.5)	44(5.5)
Agrarian vs. (Trading/Fishing)	**0.002**	

### Map: Geospatial distribution of RVFV seropositivity in south-central Uganda (June 2018–October 2020)

**Fig. 1:**
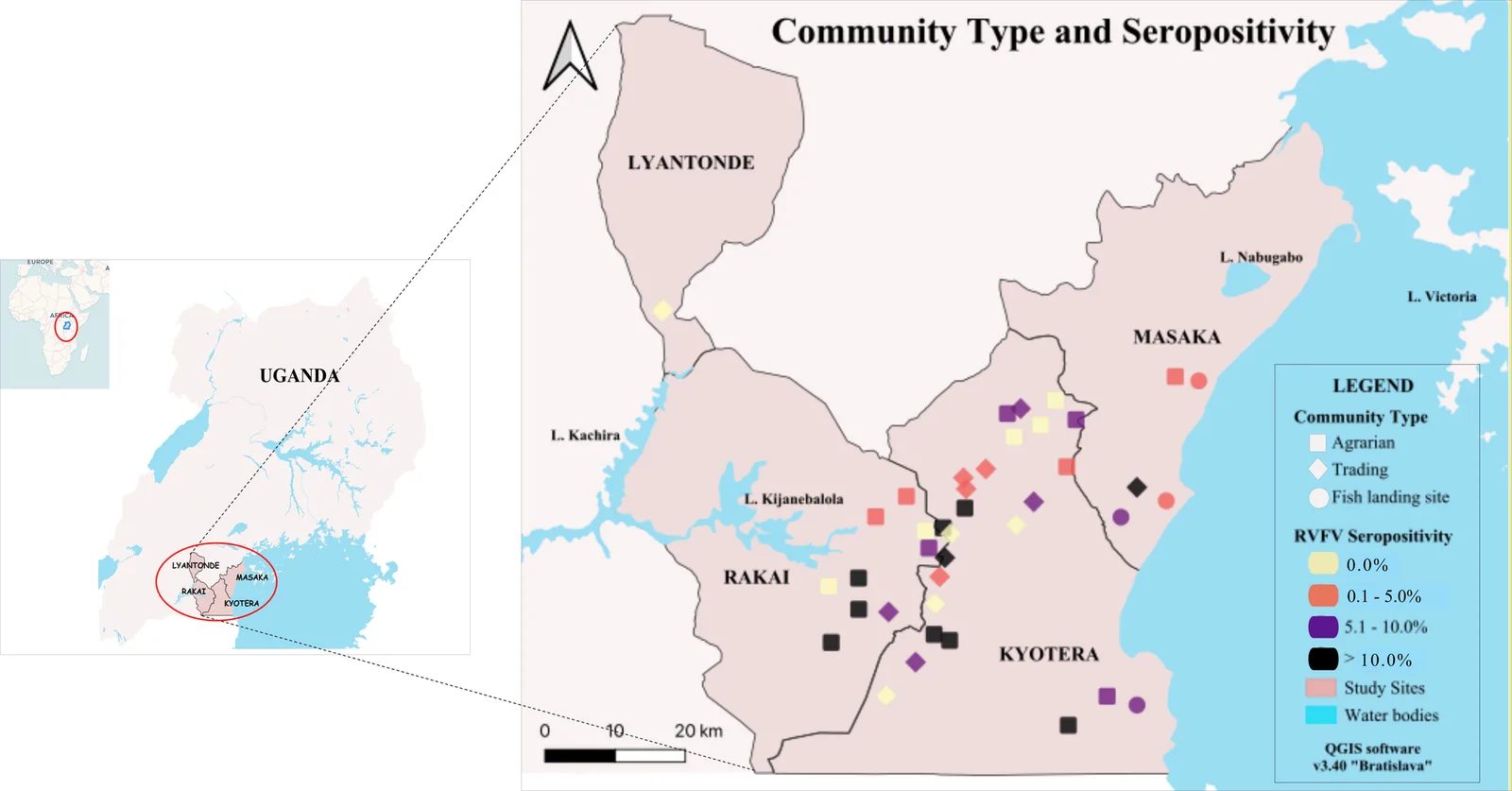
**A** thematic point map showing the spatial distribution of the 41 RCCS communities included in the study across Rakai, Kyotera, Masaka, and Lyantonde districts, Uganda. Each point represents a community, with shape indicating community type (agrarian, trading, and fishing landing sites) and color representing RVFV seropositivity levels. Major water bodies and district boundaries are shown to provide environmental and geographic context. Figure adapted from Mihalakakos et al. [35]

### Factors associated with RVFV seropositivity

Multivariable analysis identified several significant positive predictors of RVFV seropositivity: individuals residing in agrarian communities had higher odds of seropositivity than those in trading/fishing communities (adjusted odds ratio [aOR] = 2.34; 95% CI: 1.41–3.88, *p* = 0.001). Age 25–34 years (aOR = 2.00; 95% CI:1.03–3.89, *p* = 0.042) and ≥ 35 years (aOR = 2.91; 95% CI: 1.53–5.53, *p* = 0.001), compared with 15–24 years, were also associated with higher odds. Pig ownership was associated with a lower likelihood of RVFV exposure compared with non-owners (aOR = 0.40; 95% CI: 0.23–0.69, *p* = 0.001). Other factors, including gender, education, occupation type, bed net use, cattle and goat ownership, and cultivable land, were not significantly associated with seropositivity in the multivariable model (Table 3). Although RVFV is primarily mosquito-borne, and bed net use was generally high across communities, they offer limited protection against RVFV vectors, mainly *Aedes* and *Culex* mosquitoes, which often bite outdoors or during daylight and dusk hours. Since RVFV seroprevalence did not differ significantly between trading and fishing communities (*p* = 0.541), these two were combined into a single reference category for regression analyses to increase statistical power.

**Table 3:** Variable-specific burden of RVFV and factors associated with RVFV

Variable	RVFV status % (*n*/*N*)	Univariate		Multivariable	
		uOR (95% CIs)	*p*-value	aOR (95% CIs)	*p*-value
**Community type**					
Trading/Fishing	5.5% (44/799)	Ref		Ref	
Agrarian	10.5% (42/400)	**2.01(1.30–3.13)**	**0.002**	**2.34(1.41–3.88)**	**0.001**
**Gender**					
Female	6.3% (38/607)	Ref		Ref	
Male	8.1% (48/592)	1.32(0.85–2.05)	0.216	1.25(0.78-2.00)	0.352
**Age (years)**					
15–24	3.4% (17/449)	Ref		Ref	
25–34	7.4% (28/378)	**2.03(1.09–3.77)**	**0.025**	**2.00(1.03–3.89)**	**0.042**
≥ 35	11.0% (41/372)	**3.15(1.76–5.64)**	**< 0.001**	**2.91(1.53–5.53)**	**0.001**
Education*					
None	10.5% (4/38)	Ref		Ref	
Primary	9.0% (54/598)	0.84(0.29–2.47)	0.756	0.97(0.32–2.93)	0.955
Secondary/ Tertiary	5.0% (28/563)	0.44(0.15–1.34)	0.150	0.61(0.19–1.95)	0.407
**Occupation**					
Outdoor/Animal-Related	8.3% (64/773)	Ref		Ref	
Formal/Structured	4.8% (9/189)	**0.55(0.27–1.13)**	**0.106**	0.96(0.42–2.20)	0.920
Informal/Unstructured	5.5% (13/237)	**0.64(0.35–1.19)**	**0.159**	0.82(0.42–1.58)	0.554
**Livestock Ownership**					
**Own goats**					
No	7.1% (66/926)	Ref		Ref	
Yes	7.3% (20/273)	1.03(0.61–1.73)	0.911	1.02(0.57–1.84)	0.934
**Own Cattle**					
No	6.9% (73/1046)	Ref		Ref	
Yes	8.5% (13/153)	1.24(0.67–2.29)	0.498	1.42(0.73–2.77)	0.300
**Own pigs**					
No	8.5% (65/768)	Ref		Ref	
Yes	4.9% (21/431)	**0.55(0.33–0.92)**	**0.022**	**0.40(0.23–0.69)**	**0.001**
**Bed net use**					
Uses bed net	7.0% (75/1073)	Ref		Ref	
Does not use	8.7% (11/126)	1.27(0.66–2.47)	0.475	0.85(0.43–1.68)	0.634
Cultivable land**					
Has cultivable land	7.8% (66/846)	Ref		Ref	
No land	5.7% (20/353)	**0.71(0.42–1.19)**	**0.193**	0.78(0.42–1.42)	0.410

uOR = Unadjusted Odds Ratio; aOR = adjusted Odds Ratio, CI = Confidence Interval; Ref = Reference category* Education is a confounder to age; **Cultivable land is a confounder to community typePost-hoc pairwise comparisons (Bonferroni-adjusted) for age, education, and occupation at the multivariable level did not reveal statistically significant differences between non-reference subgroups (all p > 0.05), indicating that the observed primary differences in exposure occur between the reference and the other categories, rather than between non-reference subgroups

### Livestock ownership and community type

Livestock ownership (cows, goats, or pigs) and community type were assessed to examine how these factors relate to RVFV seropositivity (Table 4), given that livestock contact is a key exposure pathway for human infection. Individuals in Agrarian communities had significantly higher prevalence compared with those in Trading/Fishing areas, independent of livestock ownership. Goat and cattle ownership were not associated with seropositivity. Pig ownership was associated with a lower burden in Trading/Fishing areas, while no significant effect was observed in Agrarian communities.

**Table 4:** Interaction of livestock ownership and community type with RVFV status

Livestock x Community	RVFV status % (n/N)		Univariate				Multivariable		
			uOR (95% CIs)		*p*-value		aOR (95% CIs)		*p*-value
**Goat ownership versus community type**									
No goats: Trading/Fishing		5.6% (37/664)		Ref				Ref	
No goats: Agrarian		11.1% (29/262)		2.11(1.27-3.51)		0.004		2.36(1.40-3.97)	0.001
Goats: Trading/Fishing		5.2% (7/135)		0.93(0.40-2.13)		0.857		1.20(0.51-2.80)	0.673
Goats: Agrarian		9.4% (13/138)		1.76(0.91-3.41)		0.093		1.85(0.95-3.64)	0.073
**Cattle ownership versus community type**									
No cattle: Trading/Fishing		5.3% (37/704)		Ref				Ref	
No cattle: Agrarian		10.5% (36/342)		2.12(1.31-3.42)		0.002		2.29(1.40-3.73)	0.001
Cattle: Trading/Fishing		7.4% (7/95)		1.43(0.62-3.31)		0.399		1.66(0.70-3.89)	0.248
Cattle: Agrarian		10.3% (6/58)		2.08(0.84-5.15)		0.114		2.15(0.85-5.43)	0.104
**Pig ownership versus community type**									
No pigs: Trading/Fishing		6.6% (38/577)		Ref				Ref	
No pigs: Agrarian		14.1% (27/191)		2.34(1.38-3.94)		0.001		2.57(1.50-4.40)	0.001
Pigs: Trading/Fishing		2.7% (6/222)		0.39(0.16-0.95)		0.037		0.42(0.17-1.01)	0.054
Pigs: Agrarian		7.2% (15/209)		1.10(0.59-2.04)		0.77		0.94(0.46-1.92)	0.875

CI = Confidence Interval; Ref = Reference category; uOR = univariate odds ratio. aOR = adjusted odds ratio adjusted for age, education, gender, and occupation

## Discussion

The overall RVFV IgG seroprevalence observed in this study was 7.8% (95% CI: 1.12–16.57), indicating prior exposure despite the absence of reported RVF cases in the study area.

The 7.8% estimate observed aligns with previous Ugandan studies, reporting comparable seropositivity between 10% and 13% [18, 19, 38, 39], also similar to the pooled seroprevalence of 7.8% across Africa reported in a recent meta-analysis [40]. Similar levels have been observed among livestock-dependent communities across East Africa [41–44]. In contrast, higher seroprevalence (12.8–39%) has been reported in Kenya and Tanzania, particularly among high-risk groups like slaughterhouse workers and pastoralists [45–48]. These elevated levels likely reflect intensified ecological and occupational exposures, including mosquito density, proximity to wetlands or flood-prone areas, variations in livestock farming practices, cultural practices such as pastoralism, consumption of raw animal products [49], and the timing of sampling relative to RVF outbreak periods, when recent transmission may increase seroprevalence estimates.

RVFV-specific IgG antibodies typically appear 1–2 weeks post-infection and can persist for two years or longer, or even a lifetime [2]. Therefore, it is unclear when the seropositive individuals in our study were exposed to RVFV. The persistence of IgG, combined with reservoir–host dynamics, suggests silent, undetected transmission in this region, with many cases being asymptomatic, mild, misdiagnosed, or entirely missed by the health system. Livestock such as cattle and goats can serve as early indicators of RVFV circulation, where unusually high abortions or neonatal mortality often precede human cases. This may give a warning to health care systems to consider diagnoses of RVF when patients exhibiting febrile illness arrive at the clinic. However, in the absence of forewarning livestock disease, the clinical symptoms of RVFV infection overlap with those of malaria, typhoid, and other viral hemorrhagic fevers, increasing the likelihood of misdiagnosis in endemic settings [50–52]. For example, during the 2016 RVF outbreak in Kabale District, Uganda, initial cases presented with non-specific febrile symptoms and were not recognized as RVFV infections until molecular testing confirmed the diagnosis [53].

A recent perspective by Rostal et al. proposes that RVFV transmission may exist as a spectrum with “epidemic” regions exhibiting notable but sporadic epidemics, whereas “hyperendemic regions” may have few or no recognized outbreaks despite sustained low-level circulation [54]. Our human seroprevalence data in the absence of reported RVFV outbreaks in the area suggests this region of Uganda may be “hyperendemic” for RVFV. However, further studies are needed to understand vector ecology and RVFV dynamics in livestock species in this region of Uganda. Such cryptic circulation contributes to a hidden disease burden, and reliance solely on reported or symptomatic cases may underestimate true prevalence and delay outbreak detection within this and similar regions.

In this study, community type was significantly associated with RVFV exposure. Individuals in agrarian communities had 2.34 times higher odds of seropositivity than those in trading or fishing areas, consistent with studies from Kenya, Tanzania, and Uganda linking elevated exposure to agricultural and livestock-related activities [39, 45, 49, 55]. Agrarian communities, by definition, rely on crop cultivation and livestock rearing, and are situated in areas with extensive arable land, introducing both occupational and ecological exposure risks. Routine practices such as herding, assisting with birthing, milking, and slaughter entail close, frequent contact with livestock and potentially infectious animal fluids. However, interaction analyses indicated that the increased odds observed in the agrarian areas persisted independent of individual livestock ownership. This suggests that additional factors, such as proximity to flood-prone areas, standing water, and dense vegetation that support year-round breeding of mosquitoes, also contribute to the risk [56–58]. Together, these occupational and ecological factors explain a high-risk interface for RVFV exposure observed in agrarian communities.

Individuals aged 25–34 years had 2 times the odds of exposure, and those aged 35 years and above had nearly 3 times the odds compared to the 15–24-year age group. This likely reflects that these age groups have had more cumulative exposure to infection, and not necessarily due to their increased biological susceptibility to RVFV. Similar findings have been reported across East Africa and Ethiopia [39, 59, 60], reflecting cumulative exposure through prolonged involvement in livestock activities and repeated contact with mosquito vectors. Pig ownership was associated with a lower burden of RVF, with households that kept pigs showing 0.4 times the odds of seropositivity compared with non-owners, particularly within Trading and Fishing communities. Although pigs are not considered major amplifying hosts for RVFV, studies report low susceptibility [61] and minimal viral replication in pigs [62, 63]. However, this pattern in our data may also reflect distinct household-level husbandry patterns for households with cattle and goats versus pigs, rather than a direct protective effect of pigs themselves.

In addition to RVFV, previous analyses of this cohort have revealed exposure to another zoonotic pathogen, CCHFV, with an overall seroprevalence of 4.25% [35]. Seropositivity for CCHFV was significantly associated with cow and pig ownership, reflecting the established role of livestock in CCHFV transmission [64, 65]. Community type also influenced CCHFV exposure, with fishing communities exhibiting higher seroprevalence than agrarian or trading communities. Notably, 11 individuals were seropositive for both CCHFV and RVFV (3, 7, and 1 in agrarian, fishing, and trading communities, respectively), highlighting overlapping exposure risks or shared ecological determinants.

This study has some limitations: (1) The cross-sectional design limits causal inferences; however, careful multivariable modeling was applied, and conclusions focused on associations rather than causality. (2) Using archived samples and data, some key variables like febrile illness history and mosquito bite frequency were not available for assessment, although, where possible, proxy variables that best approximated key exposures were carefully selected and interpreted within context. (3) Seroprevalence estimates were based solely on IgG detection, reflecting past exposure, including among symptomatic individuals, and were interpreted as cumulative exposure rather than current transmission intensity. (4) Selective survival bias may underestimate the true burden if severe or fatal cases that were absent could have died before sample collection. (5) Random error, especially in variables with low counts, may have diluted true associations; this was considered when interpreting exploratory analyses. (6) Our geographical scope was limited to selected communities in the greater Masaka-Rakai region, which restricts generalizability; however, ecological relevance to RVFV transmission was prioritized, and extrapolation was applied cautiously.

## Conclusion

Our data indicate silent RVFV circulation in south-central Uganda without reported outbreaks or clinical RVF cases. Variation across community types suggests that exposure is strongly influenced by local ecological and livelihood-related factors. These findings highlight the need for strengthened, One-Health RVFV surveillance, integration of RVFV testing into routine febrile illness screening, improved clinical awareness, and integrated vector management strategies to enhance early detection and response in both outbreak and inter-epidemic periods. These findings also demonstrate the value of the RCCS as a platform for investigating zoonotic disease burden in Uganda.

## Supplementary Information

Below is the link to the electronic supplementary material.

Supplementary Material 1

Supplementary Material 2

Supplementary Material 3

## Data Availability

The data used in this study can be requested from datarequests@rhsp.org.

## Abbreviations

aOR: adjusted Odds Ratio
CCHFV: Crimean–Congo Hemorrhagic Fever Virus
CEU: Clinical Epidemiology Unit
CFR: Case Fatality Rate
CI: Confidence Interval
cELISA: competitive ELISA
ELISA: Enzyme–Linked Immunosorbent Assay
FAO: Food and Agriculture Organization
GPS: Global Positioning System
HRP: Horseradish peroxidase
IgG: Immunoglobulin G
IgM: Immunoglobulin M
OD: Optical Density
PBS: Phosphate Buffered Saline
RCCS: Rakai Community Cohort Study
RHSP: Rakai Health Sciences Program
RT: Room temperature
RVF: Rift Valley Fever
RVFV: Rift Valley Fever Virus
uOR: unadjusted Odds Ratio

## References

[1] Bouloy M, Weber F. Molecular biology of rift valley Fever virus. Open Virol J. 2010;4:8–14. 10.2174/1874357901004010008 PMC2878978 20517489

[2] Ikegami T, Makino S. The Pathogenesis of Rift Valley Fever. Viruses. 2011;3:493–519. 10.3390/v3050493 PMC3111045 21666766

[3] Ali H, et al. Rift Valley Fever: Insights into Abortive and Zoonotic Disease. Volume 3. Unique Scientific Publishers, Faisalabad, Pakistan: Zoonosis; 2023. pp. 609–24.

[4] Centers for disease control and prevention. Rift Valley fever (RVF). Centers for disease control and prevention; 2024 [cited 2026 Jun 30]. Available from: https://www.cdc.gov/rift-valley-fever/about/index.html

[5] Bastard J, et al. Reconstructing Mayotte 2018–19 Rift Valley Fever outbreak in humans by combining serological and surveillance data. Commun Med. 2022;2(1):163. 10.1038/s43856-022-00230-4 PMC9772320 36543938

[6] Bastard J, et al. Reconstructing Mayotte 2018-19 Rift Valley Fever outbreak in humans by combining serological and surveillance data. Commun Med. 2022;2:163. 10.1038/s43856-022-00230-4 PMC9772320 36543938

[7] Wright D, et al. Rift Valley fever: biology and epidemiology. J Gen Virol. 2019;100:1187–99. 10.1099/jgv.0.001296 PMC7613496 31310198

[8] Mohamed M, et al. Epidemiologic and clinical aspects of a Rift Valley fever outbreak in humans in Tanzania, 2007. Am J Trop Med Hyg. 2010;83:22–7. 10.4269/ajtmh.2010.09-0318 PMC2913502 20682902

[9] Anywaine Z, et al. Clinical manifestations of Rift Valley fever in humans: Systematic review and meta-analysis. PLoS Negl Trop Dis. 2022;16:e0010233. 10.1371/journal.pntd.0010233 PMC8986116 35333856

[10] Waqar MA, et al. Epidemiology, Clinical Manifestations, Treatment Approaches and Future Perspectives of Rift Valley Fever: Epidemiology of Rift Valley Fever. Pakistan J Health Sci. 2023;4:02–8.

[11] Petrova V, et al. Rift valley fever: diagnostic challenges and investment needs for vaccine development. BMJ Global Health. 2020;5:e002694. 10.1136/bmjgh-2020-002694 PMC7437696 32816810

[12] Balkhy HH, Memish ZA. Rift Valley fever: an uninvited zoonosis in the Arabian peninsula. Int J Antimicrob Agents. 2003;21:153–7. 10.1016/s0924-8579(02)00295-9 12615379

[13] Shoemaker T, et al. Genetic analysis of viruses associated with emergence of Rift Valley fever in Saudi Arabia and Yemen, 2000-01. Emerg Infect Dis. 2002;8:1415–20. 10.3201/eid0812.020195 PMC2738516 12498657

[14] Nanyingi MO, et al. A systematic review of Rift Valley Fever epidemiology 1931–2014. Infect Ecol Epidemiol. 2015;5:28024. 10.3402/iee.v5.28024 PMC4522434 26234531

[15] Mendy T. RVF in Senegal-summary brief-GloPID-R-pandemic PACT. 2025.

[16] Hasahya E, et al. Analysis of patterns of livestock movements in the Cattle Corridor of Uganda for risk-based surveillance of infectious diseases. Front Vet Sci. 2023;10 10.3389/fvets.2023.1095293 PMC9899994 36756309

[17] Aceng FL, et al. Rift valley fever outbreak in Sembabule District, Uganda, December 2020. One Health Outlook. 2023;5:16. 10.1186/s42522-023-00092-3 PMC10680244 38012800

[18] Nyakarahuka L, et al. Detection of Sporadic Outbreaks of Rift Valley Fever in Uganda through the National Viral Hemorrhagic Fever Surveillance System, 2017–2020. Am J Trop Med Hyg. 2023;108:995–1002. 10.4269/ajtmh.22-0410 PMC10160879 36913925

[19] Nyakarahuka L, et al. Ten outbreaks of rift valley fever in Uganda 2016–2018: epidemiological and laboratory findings. Int J Infect Dis. 2019;79:4.

[20] Kwaśnik M, Rożek W, Rola J. Rift Valley Fever - a Growing Threat To Humans and Animals. J Veterinary Res. 2021;65:7–14. 10.2478/jvetres-2021-0009 PMC8009587 33817390

[21] Asindu M, et al. Drivers and risk factors of Rift Valley fever in Uganda: findings from a preliminary survey. ILRI Research Brief; 2021.

[22] Asindu M, et al. Drivers and risk factors of Rift Valley fever in Uganda: findings from a preliminary survey. 2021.

[23] Oyas H, et al. Enhanced surveillance for Rift Valley Fever in livestock during El Niño rains and threat of RVF outbreak, Kenya, 2015–2016. PLoS Negl Trop Dis. 2018;12:e0006353. 10.1371/journal.pntd.0006353 PMC5919633 29698487

[24] Nuwagira U, Yasin I. Review of the Past, Current, and the Future Trend of the Climate Change and its Impact in Uganda. East Afr J Environ Nat Resour. 2022;5:115–26.

[25] Alkan C, Jurado-Cobena E, Ikegami T. Advancements in Rift Valley fever vaccines: a historical overview and prospects for next generation candidates. npj Vaccines. 2023;8:171. 10.1038/s41541-023-00769-w PMC10625542 37925544

[26] Faburay B, et al. Current Status of Rift Valley Fever Vaccine Development. Vaccines. 2017;5:29. 10.3390/vaccines5030029 PMC5620560 28925970

[27] Who. Rift Valley Fever. 2018. Available from: https://www.who.int/news-room/fact-sheets/detail/rift-valley-fever

[28] Simonet PNR, et al. Diversity and Abundance of Potential Vectors of Rift Valley Fever Virus in the North Region of Cameroon. Insects. 2020;11:814. 10.3390/insects11110814 PMC7699143 33227891

[29] FAO. Preparedness for Rift Valley Fever in East African Countries. 2024; Available from: https://www.fao.org/animal-health/animal-diseases/rift-valley-fever/en

[30] Situma S, et al. Widening geographic range of Rift Valley fever disease clusters associated with climate change in East Africa. medRxiv. 2024 May 17. Available from: 10.1101/2024.05.17.24307534. PMC11168176 38857944

[31] Waller L, et al. Geostatistical Modeling and Prediction of Rift Valley Fever Seroprevalence among Livestock in Uganda. Am J Trop Med Hyg. 2023;108:712–21. 10.4269/ajtmh.22-0555 PMC10076992 36878208

[32] Wawer MJ, et al. Control of sexually transmitted diseases for AIDS prevention in Uganda: a randomised community trial. Lancet. 1999;353:525–35. 10.1016/s0140-6736(98)06439-3 10028980

[33] Grabowski MK, et al. HIV Prevention Efforts and Incidence of HIV in Uganda. N Engl J Med. 2017;377:2154–66. 10.1056/NEJMoa1702150 PMC5627523 29171817

[34] Chang LW, et al. Heterogeneity of the HIV epidemic in agrarian, trading, and fishing communities in Rakai, Uganda: an observational epidemiological study. Lancet HIV. 2016;3:e388–96. 10.1016/S2352-3018(16)30034-0 PMC4973864 27470029

[35] Mihalakakos EA, et al. Longitudinal seroprevalence of Crimean-Congo hemorrhagic fever virus in Southern Uganda. Emerg Microbes Infections. 2025;14:2465315. 10.1080/22221751.2025.2465315 PMC11878160 39945753

[36] StataCorp. Stata Statistical Software: Release 18. 2023; Available from: https://www.stata.com

[37] Kortekaas J, et al. European ring trial to evaluate ELISAs for the diagnosis of infection with Rift Valley fever virus. J Virol Methods. 2013;187(1):177–81. 10.1016/j.jviromet.2012.09.016 23022325

[38] Odinoh R, et al. Have you heard of Rift Valley fever? Findings from a multi-country study in East and Central Africa. medRxiv. 2024 Dec 2. Available from: 10.1101/2024.12.02.24318288. PMC12221053 40601695

[39] Nyakarahuka L, et al. Prevalence and risk factors of Rift Valley fever in humans and animals from Kabale district in Southwestern Uganda, 2016. PLoS Negl Trop Dis. 2018;12:e0006412. 10.1371/journal.pntd.0006412 PMC5953497 29723189

[40] Ebogo-Belobo JT, et al. Contemporary epidemiological data of Rift Valley fever virus in humans, mosquitoes and other animal species in Africa: A systematic review and meta-analysis. Veterinary Med Sci. 2023;9:2309–28. 10.1002/vms3.1238 PMC10508527 37548116

[41] Kumalija MS, et al. Detection of Rift Valley Fever virus inter-epidemic activity in Kilimanjaro Region, North Eastern Tanzania. Global Health Action. 2021;14:1957554. 10.1080/16549716.2021.1957554 PMC8381910 34415237

[42] Sumaye RD, et al. Inter-epidemic acquisition of Rift Valley fever virus in humans in Tanzania. PLoS Negl Trop Dis. 2015;9:e0003536. 10.1371/journal.pntd.0003536 PMC4344197 25723502

[43] Ochieng C, et al. Seroprevalence of infections with dengue, Rift Valley fever and chikungunya viruses in Kenya, 2007. PLoS ONE. 2015;10:e0132645. 10.1371/journal.pone.0132645 PMC4503415 26177451

[44] Ndishimye P, et al. Rift Valley Fever outbreaks in the East African Community: insights from ProMed data (2010–2024). Front Public Health. 2024;12:1298594. 10.3389/fpubh.2024.1298594 PMC11668691 39722722

[45] Muturi M, et al. Ecological and subject-level drivers of interepidemic Rift Valley fever virus exposure in humans and livestock in Northern Kenya. Sci Rep. 2023;13:15342. 10.1038/s41598-023-42596-y PMC10504342 37714941

[46] Nyamota R, et al. Seroprevalence of Brucella spp. and Rift Valley fever virus among slaughterhouse workers in Isiolo County, northern Kenya. PLoS Negl Trop Dis. 2023;17:e0011677. 10.1371/journal.pntd.0011677 PMC10581456 37797043

[47] Heinrich N, et al. High seroprevalence of Rift Valley fever and evidence for endemic circulation in Mbeya region, Tanzania, in a cross-sectional study. PLoS Negl Trop Dis. 2012;6:e1557. 10.1371/journal.pntd.0001557 PMC3313937 22479657

[48] Rwegoshola KM, et al. Seasonal Transmission Dynamics of Rift Valley Fever in Kilimanjaro Region, Tanzania. East Afr Sci. 2023;5:14–21.

[49] Tigoi C, et al. High risk for human exposure to Rift Valley fever virus in communities living along livestock movement routes: A cross-sectional survey in Kenya. PLoS Negl Trop Dis. 2020;14:e0007979. 10.1371/journal.pntd.0007979 PMC7055907 32084127

[50] Iroh Tam P-Y, Obaro SK, Storch G. Challenges in the Etiology and Diagnosis of Acute Febrile Illness in Children in Low- and Middle-Income Countries. J Pediatr Infect Dis Soc. 2016;5(2):190–205. 10.1093/jpids/piw016 PMC7107506 27059657

[51] Ashraf S, et al. Uncovering the viral aetiology of undiagnosed acute febrile illness in Uganda using metagenomic sequencing. Nat Commun. 2025;16(1):2844. 10.1038/s41467-025-57696-8 PMC11930947 40122843

[52] Rugarabamu S, et al. Viral haemorrhagic fevers and malaria co-infections among febrile patients seeking health care in Tanzania. Infect Dis Poverty. 2022;11(1):33. 10.1186/s40249-022-00959-z PMC9036688 35462550

[53] Shoemaker TR, et al. First Laboratory-Confirmed Outbreak of Human and Animal Rift Valley Fever Virus in Uganda in 48 Years. Am J Trop Med Hyg. 2019;100(3):659–71. 10.4269/ajtmh.18-0732 PMC6402942 30675833

[54] Rostal MK, et al. Rift Valley fever epidemiology: shifting the paradigm and rethinking research priorities. Lancet Planet Health. 2025;9(9):101299. 10.1016/j.lanplh.2025.101299 40921175

[55] Ahmed A, et al. Sero-prevalence and spatial distribution of Rift Valley fever infection among agro-pastoral and pastoral communities during Interepidemic period in the Serengeti ecosystem, northern Tanzania. BMC Infect Dis. 2018;18:276. 10.1186/s12879-018-3183-9 PMC6001121 29898686

[56] Makhanthisa TI, et al. Characterization of mosquito host-biting networks of potential Rift Valley fever virus vectors in north-eastern KwaZulu-Natal province, South Africa. Parasites Vectors. 2024;17:341. 10.1186/s13071-024-06416-0 PMC11323694 39138532

[57] Arum SO, et al. Distribution and diversity of the vectors of Rift Valley fever along the livestock movement routes in the northeastern and coastal regions of Kenya. Parasites Vectors. 2015;8:294. 10.1186/s13071-015-0907-1 PMC4449603 26018134

[58] Biteye B, et al. Ecological distribution and population dynamics of Rift Valley fever virus mosquito vectors (Diptera, Culicidae) in Senegal. Parasites Vectors. 2018;11:27. 10.1186/s13071-017-2591-9 PMC5759860 29316967

[59] Cook EAJ, et al. The sero-epidemiology of Rift Valley fever in people in the Lake Victoria Basin of western Kenya. PLoS Negl Trop Dis. 2017;11:e0005731. 10.1371/journal.pntd.0005731 PMC5517073 28686589

[60] Oakley RB, et al. Seroprevalence and risk factors for Q fever and Rift Valley fever in pastoralists and their livestock in Afar, Ethiopia: A One Health approach. PLoS Negl Trop Dis. 2024;18:e0012392. 10.1371/journal.pntd.0012392 PMC11376510 39178328

[61] Lubisi BA, et al. Seroprevalence of Rift valley fever in South African domestic and wild suids (1999–2016). Transbound Emerg Dis. 2020;67:811–21. 10.1111/tbed.13402 31655018

[62] Gaudreault NN, et al. Comparison of Rift Valley fever virus replication in North American livestock and wildlife cell lines. Front Microbiol. 2015;6:664. 10.3389/fmicb.2015.00664 PMC4485352 26175725

[63] Lubisi BA, et al. Experimental Infection of Domestic Pigs (Sus scrofa) with Rift Valley Fever Virus. Viruses. 2023;15:545. 10.3390/v15020545 PMC9964260 36851759

[64] Hawman DW, Feldmann H. Crimean–Congo haemorrhagic fever virus. Nat Rev Microbiol, 2023. 10.1038/s41579-023-00871-9 PMC10013989 36918725

[65] Spengler JR, Bergeron É, Rollin PE. Seroepidemiological Studies of Crimean-Congo Hemorrhagic Fever Virus in Domestic and Wild Animals. PLoS Negl Trop Dis. 2016;10(1):e0004210. 10.1371/journal.pntd.0004210 PMC4704823 26741652

