# Supplementary material for: Seroprevalence of Rift Valley fever virus and factors associated with exposure in south-central Uganda
Source: BMC Infect Dis. 2026 Jul 7;26:1751. doi: 10.1186/s12879-026-13923-1 (PMC13628802; doi:10.1186/s12879-026-13923-1)
Supplement: Supplementary file 1 [file 12879_2026_13923_MOESM1_ESM.pdf]

**RAKAI COMMUNITY COHORT STUDY (RCCS) ROUND 22 FOLLOW UP FEMALE LUGANDA QUESTIONNAIRE ver 1.0 8th May 2025**

| Qn # | Description | | Variable |
|---|---|---|---|
| | INTERVIEWER | \|__\|__\|__\|__\| | FIELD_WRKR |
| | PLACE COMP_ID HERE | PLACE LABNO HERE | STUDY_ID |
| | INT DATE | ___/___/20__ dd mm yyyy | INT_DATE |
| | START TIME | \|__\|__\|:\|__\|__\| AM/PM | |
| | VISIT # | **\|R\|2\|2\|** | VISITNO |
| | **Current ID** | | CURR_ID |
| | Super Cluster # | \|__\|__\|__\| | REGION |
| | Community # | \|__\|__\|__\| | COMM_NUM |
| | HH # | \|__\|__\|__\|__\| | HH_NUM |
| | Member # | \|__\|__\|__\| | MEMBER_NUM |
| | SEX | **F** | SEX |
| | NATIONAL ID CARD NO (NIN): | \|__\|__\|__\|__\|__\|__\|__\|__\|__\|__\|__\|__\|__\|__\|<br>______________________________ | NATION_ID |
| | VICINITY # | | VIC_NO |
| | MOBILITY: | No change ……………………………………………1<br>Move within community………………………………… 2<br>In migrant……………………………………………3<br>Had out-migrated now back in the same Household........................................................... 8<br>Other (specify)……………………………………...........9<br>____________________________<br>Had out-migrated now back in the different Household..........................................................10<br>Vicinity…………………………………………………12 | MOBILITY |
| | PHOTO: | Seen ……………………………………………………1<br>No...............................................................................2<br>Taken...........................................................................3<br>Taken/check album………………………………….....4 | PHOTO |
| | CONSENT: | RCCS Baseline ………………………………………1<br>RCCS Follow-up ………………………………….......2 | CONSENT |
| | Additional study consent | Agreed…………………………………………………...1<br>Disagreed ……………………………………...….....2 | ADS_CONSE |
| | Stored samples consent | With Study_ID............................................................1<br>Without Study_ID.......................................................2<br>Not for future studies..................................................3 | LAB_CONSE |
| | Genetic testing consent | Agreed with Study ID………………………………......1<br>Agreed without Study ID…………………....…3<br>Disagreed ………………………...…..2 | GEN_CONSE |
| | Viral/bacterial gen. Consent | Agreed with Study ID………………………………...….1<br>Agreed without Study ID…..........................................…3<br>Disagreed ……………………………………….....…..2 | VIB_CONSE |
| | Interview Location | Hub ……………………………………………………..1<br>Home ………………………………………………….. 2<br>RHSP ………………………………………………… 3<br>Kampala ……………………………………………… 4 | INT_LOC |

| Qn # | Description | | Variable |
|---|---|---|---|
| | | Masaka………………………………………………… 5<br>Other……………………..…………………………….. 6 | |
| | CHPIE | Yes CHPIE.........................................................................1<br>No CHPIE……………………………………………….. 2 | CHPIE |
| | Phone Ownership | Personal……………………………………………...........1<br>Not Personal……………………………….………....…2<br>Not Applicable (no phone) ………………..………….. 8 | CONTACT |
| | Phone Number | | PHONE_NO |
| | Anthropometric | Weight 1 (Kgs) \|__\|__\|\|__\|: \|__\|__\|<br>Weight 2 (Kgs) \|__\|__\|\|__\|: \|__\|__\|<br>Weight 3 (Kgs) \|__\|__\|\|__\|: \|__\|__\|<br><br>Height 1 (cm) \|__\|__\|\|__\|: \|__\|__\|<br>Height 2 (cm) \|__\|__\|\|__\|: \|__\|__\|<br>Height 3 (cm) \|__\|__\|\|__\|: \|__\|__\|<br><br>Waist 1 (cm) \|__\|__\|\|__\|: \|__\|__\|<br>Waist 2 (cm) \|__\|__\|\|__\|: \|__\|__\|<br>Waist 3 (cm) \|__\|__\|\|__\|: \|__\|__\| | weight<br>weight2<br>weight3<br><br>height<br>height2<br>height3<br><br>waist<br>waist2<br>waist3 |
| | | | |
| | **Names:** | | |
| Q.1d. | Usually called | Erinnya lyo gwe ani lyebatera okukuyita | COMM_NAME1 |
| | Religious names | Erinnya ely'eddiini | COMM_NAME2 |
| | Other names | Amannya amalala | COMM_NAME3 |
| | Mother's maiden name: | Mother's maiden name: | NAME_MOT |

| Qn # | Description | | | | Variable |
|---|---|---|---|---|---|
| Q.1e | How old are you?<br>**(Age in completed years) (Valid range 15- and above)** | Olina emyaka emeka? | \|__\|__\| | | AGEYRS |
| | Record confirmed age | | \|__\|__\| | | CONF_AGE |
| Q.1f | How long ago were you last interviewed by any study done by RHSP/**(If period less than a year STOP, clarify on the likely study and/or** | Wayiseewo bbanga ki okuva abasawo ba Rakai Program lwebasemba/lwebase mbyeyo okukulabako mu kunoonyereza | Days \|__\|__\|<br>Weeks \|__\|__\|<br>Months \|__\|__\|<br>Years \|__\|__\| | | {DALYLI}<br>{WEEKLI}<br>{MONTHLI}<br>{YEARLI} |

| Qn # | Description | | | | Variable |
|---|---|---|---|---|---|
| | **consult QCs and editors).** | kwonna okukolebwa ekitongole kya Rakai program ng'ogyeko okujjanjabibwa mu bulwaliro. **(If period less than a year STOP, clarify on the likely study and/or consult QCs and editors).** | | | |
| Q.1g | Have you ever gone to school? | Wali osomyeko? | Yes 1<br>No 2----> **Q.6a** | \|__\| | EDUCATE |
| Q.2 | To what level? **(Probe if got additional training) (Code highest level)** | Wakomawa **(probe if got additional training)**?<br>**(Code highest level)** | | \|__\|__\| | EDUCYRS |

P1…………01
P2…………02
P3…………03
P4…………04
P5…………05
P6…………06
P7…………07
S1…………09
S2…………10
S3…………11
S4…………12
S5…………13
S6…………14
Technical/University……………15
Primary professional……………16
O'level professional ……………17
Primary or O'level apprenticeship……18
A'level apprenticeship ……………… 19

| # | | | | | |
|---|---|---|---|---|---|
| **FOR THOSE 15-24 years please ask Q.3 else skip to Q 6** | | | | | |
| Q.3 | Are you currently enrolled in school? | Mu kiseera kino oli mu ssomero? | Yes 1--->**Q.6**<br>No 2 | \|__\| | SCHOOL |
| Q.4 | At what age did you leave school? | Walina emyaka emeka we wavira mu musomero? | DK=97 | \|__\|__\| | LAGESCH |
| | | | | | |

***Note: Code Yes in Q.5 applies for primary, secondary or university/Tertially, not to vocational /apprenticeship or informal training programs.*** If not currently enrolled or left school before completing

| Q.5 | What was the main reason you left school | Ensonga enkulu eyakuletera okuva mu ssomero | **Yes** | No | |
|---|---|---|---|---|---|
| a. | Financial | Tewaali ssente | 1 | 2 | FINSCH |
| b. | Pregnancy | Wafuna olubuto | 1 | 2 | PREGSCH |
| c. | Moved | Wava mu kifo ekimu n'ongenda mu kifo ekirala | 1 | 2 | MOVESCH |
| d. | Menarche | Watandika okugenda mu nsonga z'abakyala | 1 | 2 | MENASCH |
| e. | Sexual harassment in or on the way to school | Wakakibwa okwegatta ng'oli ku somero oba ng'ogenda ku ssomero | 1 | 2 | HARASCH |
| f. | Bullying on the way or in to school | Okutulugunyizibwa oba okutisibwatisibwa ng'ogenda oba nga oli ku somero | 1 | 2 | BULLWSCH |
| I | Parent died | Omuzadde yafa | 1 | 2 | PARESCH |
| J | Accomplished my educational goals | Wafuna/watuukiriza ebirubirirwa byo eby'okusoma. | 1 | 2 | ACCOMSCH |
| K | Left school because of COVID-19 | Wava musomero lwa bulwadde bwa COVID-19 | 1 | 2 | COVIDSCH |
| L | Other | | 1 | 2 | OTHSCH |
| | Specify | | | | SPESCH |

| | | | | |
|---|---|---|---|---|
| Q.6 | What kind of work do you do, or what kind of activities keep you busy during an Average day, whether you get money for them or not? | Okola mirimu ki? oba biki by'okola ebisinga okukumalira ebiseera mu lunaku kobeere ng'ofunamu ssente oba nedda? | \|__\|__\|<br>\|__\|__\| | OCCUP1<br>OCCUP2 |
| **((Record answer(s) as given):** __________________________________________________ | | | | **OCCUPAT2**<br>**OCCUPAT1** |

**(Code up to two responses, code first mentioned occupation first)**

Agriculture for home use/barter ………………………………………………… 01
Agriculture for selling…........................................................................... 02
Housework in your own home…..............................................................03
Housekeeper (for relative or employer) ..........................................................04
Home brewing….…..........................................................................................05
Government/clerical/teaching …......................................................................06
Fishing….….......................................................................................................07
Student …..........................................................................................................08
Military/police…................................................................................................09
Shopkeeper….....................................................................................................10
Trading/vending…............................................................................................11
Bar worker or owner........................................................................................ 12
Trucker …..........................................................................................................13 OCCUPS

Unemployed (**PROBE _ NO AGRIC OR HOUSE WORK?**) ..........................14
Other (specify) ________________________________________________ 15
No additional response (use in 2nd field if one occupation is cited………………88
Medical worker (non-government) .. ..................... ..................... ....................16
Casual laborer……………..............................................................................17
Waitress/Waiter/restaurant owner………..............................................................18
Hair dresser/Salon owner……………......................................................................19
Construction (brick maker, porter, roofing, builder, painter) ……………………..20
Mechanic (automobiles, bicycles, electronics) ……..............................................21
BodaBoda………………….........................................................................................22
Sex worker/client……………………………………………………………………23
Sports betting, Gambling machine,Ludo……………........................................24
NA ………………………………………………………………………………..98
Baking……………………………………………………………………….. 25
Mining……………………………………………………………………….. 26
Textiles………………………………………………………………………. 27
Carpentry…………………………………………………………………… 28
-

| # | | | | | |
|---|---|---|---|---|---|
| | **I would like to ask you about ways in which people can plan their families. the question will help us to know how people plan their families in Rakai and neighbouring communities.** | | **Nandyagadde okukubuuzaayo ebibuuzo bitono ebikwata ku by'okuzaala. Ebibuuzo bino bijja kutuyamba okumanya embeera y'ebyokuzaala bweri mu Rakai ne bitundu ebigiriranye.** | | |
| **FOR THOSE 15-24 years please ask Q.7a else skip to Q.8** | | | | | |
| Q.7a | How old were you at the time you experienced your first menstruation? | Walina emyaka emeka omulundi lwewasokera ddala okugenda mu nsonga za bakyaala? | \|__\|___\|<br>Never Menstruated… 88<br>DK 97 | | MYEARS |
| Q.7b | Have you ever been pregnant? [Including current pregnancy] | Wali ofunye ku lubuto? (Bwoba nga oli lubuto kati lutwaliremu) | Yes 1<br>No 2 ------>Q.10<br>DK 7 ------->Q.10 | \|__\| | EVERPREG |
| Q.8 | At what age did you first get pregnant? | Walina emyaka emeka lwewasokera dala okufuna olubuto? | \|__\|__\| | | AGEPREG1 |
| Q.9 | If 55 and above skip to Q.10<br><br>Are you currently pregnant? | Oli lubuto kati? | Yes 1<br>No 2<br>DK 7 | \|__\| | PREGNOW |
| Q.10 | If you got pregnant now, how would you feel? | Singa ofuna olubuto kati | Very happy 5<br><br>Somewhat happy 4<br><br>Mixed (happy and not happy 3 | \|__\| | |

| | | | | | |
|---|---|---|---|---|---|
| | | | Unhappy 2<br>Very unhappy 1 | | |
| Q.11 | How many living children do you have? | Olina abaana bameka bozaala abakyali abalamu? | \|__\|__\|<br>If no living children 00<br>NA 98 for never been pregnant | | NUMCHILD |
| | ***IF PREGNANT CODE 8***<br>**I would like to ask you some questions about family planning** | | **Nandyagadde okukubuuzaayo ebibuuzo ebikwata ku ngeri abantu gye bayinza okwegemamu okuzaala oba okwegema okufuna embuto amangu.** | | |
| Q.12 | Are you currently using any family planning method? | Olina engeri yonna ey'okwegema okuzaala / okwegema okufuna embuto amangu gyokozesa kati? | Yes 1<br>No 2<br>N/A(pregnant) 8 | \|__\| | FPUSING |
| Q.13 | If yes what methods are you currently using? (Unprompted) | | If yes Ngeri ki gyokozesa / zokozesa kati okwegema okuzaala / okwegema okufuna embuto amangu? | | |
| | **Current use** | Yes | No | NA | |
| | Pills | 1 | **2** | 8 | FPUSING1 |
| | Condom | 1 | **2** | 8 | FPUSING2 |
| | Depot Prep/injection | 1 | **2** | 8 | FPUSING4 |
| | Sayana Plus/self FP injection | 1 | **2** | 8 | FPUSNG17 |
| | IUD/coil | 1 | **2** | 8 | FPUSING7 |
| | Implant/ Norplant | 1 | **2** | 8 | FPUSNG12 |
| | BTL/VASECTOMY | 1 | **2** | 8 | FPUSNG11 |
| | Spermicides | 1 | **2** | 8 | FPUSING3 |
| | Abstinence | 1 | **2** | 8 | FPUSING5 |
| | Calendar | 1 | **2** | 8 | FPUSING6 |
| | Lactation | 1 | **2** | 8 | FPUSING9 |
| | Herbs | 1 | **2** | 8 | FPUSNG10 |
| | Other method | 1 | **2** | 8 | FPUSING8 |
| | Specify | | | | OTHFP |

| # | | | | | |
|---|---|---|---|---|---|
| Q.15 | Have you ever been married or entered a consensual union?<br>**(If present union is the ONLY marriage ever, code Yes)** | Wali ofumbiddwako (obufumbo obw'engeri yonna nebwoba nga tewali / toli mugatte)? | Yes 1<br>No 2------>**Q.**19<br>DK 7----->**Q.19** | \|__\| | EVERMARR |
| Q.16 | How old were you the first time you got married? | Walina emyaka emeka lwewasokera ddala okufumbirwa? | \|__\|__\|DK= 97 | | AG1STMAR |
| Q.17 | Are you currently married (whether traditional, civil or | Kakati oli mufumbo (obufumbo obw'engeri | Yes 1 | \|__\| | CURRMARR |

| | | | | | |
|---|---|---|---|---|---|
| | religious, or in a consensual union)? | yonna ne bwoba nga toli mugatte)? | No 2-------->Q.19 | | |
| Q.18 | How many wives does your husband have? [**Record actual # wherever possible, otherwise[code 92=(01-02), 93=3-5, 94=6-10, 95= 11-20, 96=21-30, 98= 31-40, 99=41 and above DK=97** | Omwami wo alina abakyala bameka? | \|__\|__\| | | POLYMAR |
| Q.19 | Since the last RCCS visit, have you had a change in your sexual relationship | Okuva lwetwasembayo okukukyalira mu nkyala zaffe ezabuli mwaka, obufumbo bwo oba enkolagana yo ne muganzi wo/baganzi bo ekyuseeko? | Yes 1<br>No 2---**Q.21**<br>DK 7----->**Q.21** | \|__\| | CHANGESR |
| Q.20 | **If yes**, how has it changed? **(PROBE and code below)** | | **If yes**, Ekyuse etya? **PROBE and code below under "since last seen")** | | |
| | | | Yes | No | |
| | Engaged | Yayanjula | 1 | 2 | STATVST1 |
| | Married | Yafumbirwa | 1 | 2 | STATVST2 |
| | Ended a non-marital | Baakyawagana n'omuganzi | 1 | 2 | STATVST3 |
| | Acquired non- marital | Yafuna omuganzi | 1 | 2 | STATVST4 |
| | Divorced/ separated marital | Yanoba/baayawukana | 1 | 2 | STATVST5 |
| | Widowed | Yafiirwa omwami | 1 | 2 | STATVST6 |
| | Death of a non- marital partner | Yafiirwa omuganzi | 1 | 2 | STATVST7 |
| | Divorced/separated with marital then got back together | Yanoba/bayawukana ne badingana | 1 | 2 | STATVST9 |
| | Separated with non-marital then got back Together | Bakyawagana n'omuganzi ne badingana | 1 | 2 | STATVST10 |
| | Other | | 1 | 2 | OSTATVST |
| | Specify | | | | OTHSTATVST |
| Q.21 | Have you had sexual intercourse with any person in the last 12 months? | Mu myezi ekkumi n'ebiri egiyise wegasseeko n'omuntu yenna? | Yes 1<br>No 2 ----->Q.24<br>NR 9 | \|__\| | SEXYEAR |
| **For questions 22-24 code actual number when ever possible** | | | | | |
| Q.22 | How many different sexual partners have you had in the last 12 months, including married or consensual partners and anyone already mentioned?<br>[**Record actual # wherever possible, otherwise[code 92= a few (01-02), 93=3-5,** | Mu myezi ekkumi n'ebiri egiyise, wegasse n'abantu bameka ng'obaliddemu omwami wo, muganziwo/baganzibo n'abantu abalala bonna betwogeddeko? | \|__\|__\| | | SEXP1YR |

| | | | | |
|---|---|---|---|---|
| | **94=6-10, 95= 11-20, 96=21-30, 98= 31-40, 99=41 and above** 97=DK | | | |

| # | | | | | |
|---|---|---|---|---|---|
| Q.23 | How many partners in the last twelve months were from outside this community? [**Record actual # wherever possible, otherwise[code 92= a few (01-02), 93=3-5, 94=6-10, 95= 11-20, 96=21-30, 98= 31-40, 99=41 and above DK=97** | Bameka kw'abo bewegasse nabo mu myezi ekkumi n'ebiri egiyise abataali ba mukitundu kino | \|__\|__\| | | SEXP1OUT |
| Q.24 | How many different sexual partners have you had in your lifetime including married or consensual partners? [**Record actual # wherever possible, otherwise[code 92= a few (01-02), 93=3-5, 94=6-10, 95= 11-20, 96=21-30, 98= 31-40, 99=41 and above DK=97** | Mu bulamu bwo wakegatta n'abantu bameka ng'obaliddemu omwamiwo, muganziwo/ baganzibo n'abantu abalala bonna betwogeddeko? | \|__\|__\| | | SEXPEVER |
| **IF NEVER BEEN PREGNANT OR NEVER MARRIED OR IN A RELATIONSHIP AND NO SEXUAL PARTNER IN THE PAST OR in last 12 months. (I.e Q.7b-24 ARE NO/00)THEN ASK Q.25 ELSE SKIP TO Q.26** | | | | | |
| Q.25 | Have you ever had a sexual relationship? | Wali wegasseeko n'omuntu yenna? | Yes 1<br>No 2------>**Q.76** | \|__\| | EVERSEX |
| **FIRST SEXUAL EXPERIENCE FOR WOMEN** | | | | | |
| Q.26 | How old were you the first time you had sexual intercourse? **Record completed years, DK=97, NR=99]** | Walina emyaka emeka lwewasookera ddala okwegatta n'omuntu yenna? | \|__\|__\| | | AG1STSX |

| COMPUTER ID | PLACE LABNO HERE | CURRENT ID<br>____/_____/______/______ |
|---|---|---|

**BEGIN REPETITIVE SEXUAL PARTNER BLOCKS (FOUR TOTAL)**

| | |
|---|---|
| **(The following blocks of questions should be asked for each current and past relationship <u>in the last 12 months up to 4 partners.</u> Begin by asking about the most recent partner. If the most recent relationship was more than a year ago, but after the date when last seen still ask about this most recent partner).** If no sex since last seen and not had sex in the last 12 months then skip all repetitive sexual blocks.<br>**"Now I would like to ask you about your most recent sex partners. Please remember that all of your answers are confidential. Your answers are very important to our research to help us understand health problems in Rakai and neigbouring districts".** | **(The following blocks of questions should be asked for each current and past relationship <u>in the last 12 months up to 4 partners.</u> Begin by asking about the most recent partner. If the most recent relationship was more than a year ago, still ask about this most recent partner).**<br><br>**"Nandyagadde okukubuuzaayo ku bibuuzo bitono ebikwata ku muntu gwewakasembayo okwegatta naye. Nkukakasa nti byonna by'onombulira bikuumibwa nga bya kyama. By'ononziramu bitwalibwa nga bya mugaso nnyo mu kunoonyereza kwaffe, kitusobozese okutegeera obulungi ebikwata ku bulamu bw'abantu ba Rakai ne districts ezigiriranye."** |

| | | | | |
|---|---|---|---|---|
| Q.27a | Remembering the most recent time you had sex, what was your relationship to that partner at that time? | Bw'ojjukira omulundi gwewasembayo okwegatta. Omuntu oyo gw'osembyeyo/gwewasembayo okwegatta naye wali omuyita otya? | \|__\|__\| | RLTN1 |
| Current husband (at the time) ………………………………. 01<br>Current consensual partner (at the time) …………………….. 02<br>Former husband/consensual partner……………………….. 03<br>Boyfriend…………………………………………………. 04<br>Occasional or casual friend…………………………………05<br>Visitor (incl. wedding/funeral) ……………………………06<br>Stranger……………………………………………………. 07<br>Workmate…………………………………………………. 08<br>Boss/work supervisor………………………………………09<br>Employee…………………………………………………. 10<br>Fellow student…………………………………………….. 11<br>Sugar Daddy ………………………………………………12<br>Relative other than spouse**(specify)__ __________________**13<br>Other non relative **(specify)________________________** 14<br>Rapist (by a stranger) …………………………………… 15<br>Don't Know ………………………………………………. 97<br>Client/sex worker…………………………………………..16 | | | | OTHRLTN1<br>OTHRLNR1 |

| | | |
|---|---|---|
| Q.27b | What are/were your partner's main occupations? (Record answer(s) as given):______________________ (OCCUPAT11)<br><br>_________________________________(OCCUPAT22)<br>**(Code up to two responses, code first mentioned occupation first.)**<br>Agriculture for home use/barter…………….01<br>Agriculture for selling……………………02<br>Housework in own home…………….. 03<br>Housekeeper (for relative or employer)…….04 | \|__\|__\|{OCCUP11}<br>\|__\|__\|{OCCUP21}<br><br>Omuntu ono yali/ akola mirimu ki? oba biki by'akola/ byeyali akola ebisinga okukumalira ebiseera mu lunaku kabeere ng'afunamu /yali afunamu |

| | | | | | |
|---|---|---|---|---|---|
| | | Home brewing ……………………………. 05<br>Government/clerical/teaching …………….. 06<br>Fishing…………………………………….. 07<br>Student………………………………………08<br>Military/police ……………………………...09<br>Shopkeeper ……………………………….. 10<br>Trading/vending ………………………… 11<br>Bar worker or Owner……………………….12<br>Trucker………………………………….... 13<br>Unemployed (PROBE _ NO AGRIC OR HOUSE WORK?)…………………………….. 14<br>Other __________ ………………. 15<br>Medical worker (non-government)…… ….. 16<br>Casual laborer …………………………... 17<br>Waiter/restaurant owner……………………18<br>Hair dresser/Salon owner…………………...19<br>Construction (brick marker, porter builder, painter, roofing)……………….. 20<br>Mechanic (automobiles, bicycles, electronics)…….. 21<br>Boda Boda……………………………….. 22<br>No additional response (use in 2nd field if one occupation is cited …………………………………88<br>Do Not Know …………………………... 97<br><br>Client…………………………………………... 23<br>Sports betting, Gambling machine, Ludo……….. 24<br>NA …………………………………………….98<br>Baking………………………………………….. 25<br>Mining………………………………………….. 26<br>Textiles…………………………………………. 27<br>Carpentry……………………………………… 28 | ssente oba nedda?<br><br>{OCCUPS1} | | |
| Q.27 c | How long ago did you first have sex with this person? | Wayiseewo bbanga ki okuva lwe wasookera ddala okwegatta n'omuntu ono? | Less than 1 day code 00 day,<br>Less than 1 week code day<br>Less than one month code weeks<br>Less than one year code months<br>If 1 year or more code completed Years<br>(If DK Code 97 in days and 98 in other boxes; 99 for NR) | \|__\|__\|<br>\|__\|__\|<br>\|__\|__\|<br>\|__\|__\| | DAYS1<br>WEEKS1<br>MONTHS1<br>YEARS1 |
| Q.28 | How long ago, did you last have sex with this person? | Wasemba ddi okwegatta naye? | Less than 1 day code 00 days<br>Less than a week code days | \|__\|__\|<br>\|__\|__\|<br>\|__\|__\|<br>\|__\|__\| | RLDYSLT1<br>RLWKSLT1<br>RLMOSLT1<br>RLYYSLT1 |

| | | | | | |
|---|---|---|---|---|---|
| | | | Less than a month code weeks<br>Less than one year code months<br>If one year or more code completed year Year<br>(if DK code 97 in days and 98 9n other boxes; 99 for NR) | | |
| Q.29 | Are you still in a sexual relationship with him? | Okyegatta n'omuntu oyo? | Yes 1<br>No 2<br>DK 7 | \|__\| | RLTONGO1 |
| Q.30 | Does (was) he live (living) in this household ? | Musula (Mwali musula) wamu? | Yes 1……………Q.32<br>No 2<br>DK 7 | \|__\| | RLTNH1 |
| Q.31 | Does (was) he regularly live (living) in this community? | Abeera (Yali abeera) mu kitundu kino ebiseera ebisinga? | Yes 1<br>No 2<br>DK 7 | \|__\| | RLTNCM1 |
| Q.32 | Is/was he older, younger, or about the same age? | Omuntu oyo (yali) muto kuggwe, nga mwenkana oba nga akusinga obukulu? | Older 1<br>Younger 2<br>Same age 3……….>Q.34<br>Do not know 7 ..Q.34 | \|__\| | RLTNAGE1 |
| Q.33 | About how many years [older/younger]? **(Record actual # or 97=don't know)** | Akusinga (Yali akusinga) oba nga omusinga emyaka emeka? **Record actual # or 97=don't know)** | | \|__\|__\| | RLTNYRS1 |
| Q.34 | Have you and this partner ever used a condom? | Ggwe n'omuntu oyo, mwali mukozesezza ku bupiira (condoms)? | Yes 1<br>No 2……>Q.36<br>NR 9……Q.36 | \|__\| | CNDEVER1 |
| Q.35 | During the most recent/ last 12 months you were having sexual relationship with this partner, how often did you use condoms? | Mu myezi ekkumi n'ebiri egisembyeyo /egyasembayo nga wegatta n'omuntu oyo, obupiira (mwali) mubukozesa buli kiseera, luusi na luusi oba temubukozesezako \temwabukozesako nakatono? | Never 1<br>Sometimes/inconsistent 2<br>Always 3<br>DK 7 | \|__\| | RNYRCON1 |
| | | | | | |
| | | | | | |
| | | | | | |
| Preamble: Sometimes people enter into relationships because they need help paying for things they need, even if that is not directly exchanged for sex. | | | **Ebiseera ebimu abantu basalawo okwegatta mu mukwano olw'okuba nti beetaga obuyambi okusobola okugula ebintu** | | |

| | | | | | |
|---|---|---|---|---|---|
| | | | **byebaba beetaga, ne bwekiba nti tekitwaliddwa buterevu nga okwetunda oba okwegatta ofune ssente.** | | |
| | | | | | |
| Q.37 | *Did you enter into a sexual relationship with this partner mainly in order to get things you need, money, gifts, or other things that are important to you* | *Wasaalawo okwegatta n'omuntu ono okusobola okufuna ebintu bye wali wetaga,ekirabo oba ebintu eby'omugaso gyoli* | Yes 1<br>No 2 | \|__\| | SEXNEEDS1 |

**Insert spouse's household current ID if spouse is in the study area and relationship still on going . If the spouse is not in study area, code 888/888/8888/888**

Spouse's Current ID**.** |__|__|__|__|/|__|__|__|__|/|__|__|__|__|__|/|__|__|__|__| **P1CURR_ID**

| **SECOND BLOCK** | | | | | |
|---|---|---|---|---|---|
| **"Can you please tell me about the sexual partner just prior to the one we just discussed?" (In the last twelve month)** | | | **"Nandyagadde okukubuuzayo ebibuuzo bitono ebikwata ku muntu omulala gwewegatta naye mumyezi ekkumi n'ebiri egiyise eyaddibwako oyo gwetwogeddeko. (In the last twelve months)** | | |
| Q.36 b | Have you had any other sexual partners in the past 12 months other than the one we just discussed? | Mu myezi ekkumi n'ebiri egiyise, wali wegasse n'omuntu omulala yenna eyaddibwako oyo gwetwogeddeko? | **Yes 1**<br>**No 2………Q.76** | \|__\| | MORE2 |
| Q.38 | Remembering the most recent time you had sex, what was your relationship to that partner at that time? | Omuntu oyo gw'osembyeyo/gwewasembayo okwegatta naye wali omuyita otya? | | \|__\|__\| | RLTN2 |
| Current husband(at the time) …………………………………….. 01<br>Current consensual partner (at the time) …………………….. 02<br>Former husband/consensual partner……………………………… 03<br>Boyfriend…………………………………………………… 04<br>Occasional or casual friend……………………………………05<br>Visitor (incl. wedding/funeral) ……………………………….06<br>Stranger……………………………………………………. 07<br>Workmate…………………………………………………. 08<br>Boss/work supervisor……………………………………….09<br>Employee…………………………………………………. 10<br>Fellow student……………………………………………….. 11 | | | | | |

| | | |
|---|---|---|
| Sugar Daddy …………………………………………….12<br>Relative other than spouse**(specify)__** ___________________13<br>Other non relative **(specify)__________________________** 14<br>Rapist (by a stranger) ……………………………………. 15<br>Don't Know ……………………………………………… 97<br>Client/sex worker………………………………………….16 | OTHRLTN2<br>OTHRLNR2 | |

| | | | | | |
|---|---|---|---|---|---|
| Q.39 | What are/were your partner's main occupations? (Record answer(s) as given):______________________ (OCCUPAT12)<br><br>_________________________________(OCCUPAT22)<br>**(Code up to two responses, code first mentioned occupation first.)**<br>Agriculture for home use/barter…………….01<br>Agriculture for selling…………………………02<br>Housework in own home……………... 03<br>Housekeeper (for relative or employer)…….04<br>Home brewing …………………………….. 05<br>Government/clerical/teaching …………………….. 06<br>Fishing…………………………………….. 07<br>Student…………………………………………… 08<br>Military/police ……………………………...09<br>Shopkeeper ……………………………….. 10<br>Trading/vending …………………………. 11<br>Bar worker or Owner…………………………...12<br>Trucker…………………………………... 13<br>Unemployed (PROBE _ NO AGRIC OR HOUSE WORK?).. 14<br>Other ________________________…………. 15<br>Medical worker (non-government)…………… ….. 16<br>Casual laborer ……………………………………... 17<br>Waiter/restaurant owner……………………………. 18<br>Hair dresser/Salon owner…………………………... 19<br>Construction (brick marker, porter builder, painter, roofing).. 20<br>Mechanic (automobiles, bicycles, electronics)…….. 21<br>Boda Boda……………………………………….. 22 | \|__\|__\|{OCCUP12}<br>\|__\|__\|{OCCUP22}<br><br>Omuntu ono yali/ akola mirimu ki? oba biki by'akola/ byeyali akola ebisinga okukumalira ebiseera mu lunaku kabeere ng'afunamu /yali afunamu ssente oba nedda?<br><br>{OCCUPS2} | | | |

| | | | | | |
|---|---|---|---|---|---|
| | No additional response (use in 2nd field if one occupation is cited ………………………………………………… 88<br>Do Not Know ………………………………... 97<br>Client…………………………………………... 23<br>Sports betting, Gambling machine, Ludo………….. 24 NA ……………………………………………………………………………..98<br><br>Baking…………………………………………………………………….. 25<br>Mining…………………………………………………………………….. 26<br>Textiles……………………………………………………………………. 27<br>Carpentry…………………………………………………………………… 28 | | | | |

| | | | | | |
|---|---|---|---|---|---|
| Q.40 | How long ago did you first have sex with this person? | Wayiseewo bbanga ki okuva lwe wasookera ddala okwegatta n'omuntu ono? | Less than 1 day code 00 days,<br>Less than 1 week code days<br>Less than a month code weeks<br>Less than one year code months<br>If 1 year or more code completed Years (If DK Code 97 in days and 98 in other boxes; 99 for NR) | \|__\|__\|<br>\|__\|__\|<br>\|__\|__\|<br>\|__\|__\| | DAYS2<br>WEEKS2<br>MONTHS2<br>YEARS2 |
| Q.41 | How long ago, did you last have sex with this person? | Wasemba ddi okwegatta naye? | Less than 1 day code 00 days<br>Less than a week code days<br>Less than a one month code weeks<br>Less than one year code months<br>(if DK code 97 in days and 98 9n other boxes; 99 for NR) | \|__\|__\|<br>\|__\|__\|<br>\|__\|__\| | RLDYSLT2<br>RLWKSLT2<br>RLMOSLT2 |
| Q.42 | Are you still in a sexual relationship with him? | Okyegatta n'omuntu oyo? | Yes 1<br>No 2<br>DK 7 | \|__\| | RLTONGO2 |

<table>
<tr><td>Q.43</td><td>Does (was) he live (living) in this household ?</td><td>Musula (Mwali musula) wamu?</td><td>Yes 1……Q.45<br>No 2<br>DK 7</td><td>|__|</td><td>RLTNH2</td></tr>
<tr><td>Q.44</td><td>Does (was) he regularly live (living) in this community?</td><td>Abeera (Yali abeera) mu kitundu kino ebiseera ebisinga?</td><td>Yes 1<br>No 2<br>DK 7</td><td>|__|</td><td>RLTNCM2</td></tr>
<tr><td>Q.45</td><td>Is/was he older, younger, or about the same age?</td><td>Omuntu oyo (yali) muto kuggwe, nga mwenkana oba nga akusinga obukulu?</td><td>Older 1<br>Younger 2<br>Same age 3….>Q.47<br>Do not know 7..Q.47</td><td>|__|</td><td>RLTNAGE2</td></tr>
<tr><td>Q.46</td><td>About how many years [older/younger]? (Record actual # or 97=don't know)</td><td>Akusinga (Yali akusinga) oba nga omusinga emyaka emeka? Record actual # or 97=don't know)</td><td colspan="2">|__|__|</td><td>RLTNYRS2</td></tr>
<tr><td>Q.47</td><td>Have you and this partner ever used a condom?</td><td>Ggwe n'omuntu oyo, mwali mukozesezza ku bupiira (condoms)?</td><td>Yes 1<br>No 2……>Q.49<br>NR 9……Q.49</td><td>|__|</td><td>CNDEVER2</td></tr>
<tr><td>Q.48</td><td>During the most recent/ last 12 months you were having sexual relationship with this partner, how often did you use condoms?</td><td>Mu myezi ekkumi n'ebiri egisembyeyo /egyasembayo nga wegatta n'omuntu oyo, obupiira (mwali) mubukozesa buli kiseera, luusi na luusi oba temubukozesezako \temwabukozesako nakatono?</td><td>Never 1<br>Sometimes/inconsistent 2<br>Always 3<br>DK 7</td><td>|__|</td><td>RNYRCON2</td></tr>
<tr><td></td><td></td><td></td><td></td><td></td><td></td></tr>
<tr><td colspan="3"></td><td colspan="3"></td></tr>
<tr><td></td><td></td><td></td><td></td><td></td><td></td></tr>
<tr><td colspan="3">Preamble: Sometimes people enter into relationships because they need help paying for things they need, even if that is not directly exchanged for sex.</td><td colspan="3">Ebiseera ebimu abantu basalawo okwegatta mu mukwano olw'okuba nti beetaga obuyambi okusobola okugula ebintu byebaba beetaga, ne bwekiba nti tekitwaliddwa buterevu nga okwetunda oba okwegatta ofune ssente.</td></tr>
<tr><td></td><td></td><td></td><td></td><td></td><td></td></tr>
<tr><td>Q.49</td><td>Did you enter into a sexual relationship with this partner mainly in order to get things you need, money, gifts, or other things that</td><td>Wasaalawo okwegatta n'omuntu ono okusobola okufuna ebintu bye wali wetaga,ekirabo oba ebintu eby'omugaso gyoli</td><td>Yes 1<br>No 2</td><td>|__|</td><td>SEXNEEDS2</td></tr>
</table>

| | | | | | |
|---|---|---|---|---|---|
| | *are important to you* | | | | |

**Insert spouse's household current ID if spouse is in the study area and relationship still on going . If the spouse is not in study area, code 888/888/8888/888**

Spouse's Current ID**.** |__|__|__|/|__|__|__|/|__|__|__|__|/|__|__|__| **P2CURR_ID**

| | | | | | |
|---|---|---|---|---|---|
| **THIRD BLOCK** | | | | | |
| **"Can you please tell me about the sexual partner just prior to the one we just discussed?" (In the last twelve month)** | | **"Nandyagadde okukubuuzayo ebibuuzo bitono ebikwata ku muntu omulala gwewegatta naye mumyezi ekkumi n'ebiri egiyise eyaddibwako oyo gwetwogeddeko. (In the last twelve months)** | | | |
| Q.50 | Have you had any other sexual partners in the past 12 months other than the one we just discussed? | Mu myezi ekkumi n'ebiri egiyise, wali wegasse n'omuntu omulala yenna eyaddibwako oyo gwetwogeddeko? | **Yes 1**<br>**No 2………Q.76** | \|__\| | MORE3 |
| Q.51 | Remembering the most recent time you had sex, what was your relationship to that partner at that time? | Omuntu oyo gw'osembyeyo/gwewasembayo okwegatta naye wali omuyita otya? | | \|__\|__\| | RLTN3 |
| Current husband(at the time) …………………………………… 01<br>Current consensual partner (at the time) …………………….. 02<br>Former husband/consensual partner……………………………… 03<br>Boyfriend…………………………………………………. 04<br>Occasional or casual friend……………………………………05<br>Visitor (incl. wedding/funeral) ………………………………06<br>Stranger…………………………………………………… 07<br>Workmate…………………………………………………. 08<br>Boss/work supervisor………………………………………09<br>Employee…………………………………………………. 10<br>Fellow student…………………………………………….. 11<br>Sugar Daddy ……………………………………………12<br>Relative other than spouse**(specify)**__ ___________________13<br>Other non relative **(specify)**__________________________ 14<br>Rapist (by a stranger) …………………………………… 15<br>Don't Know ……………………………………………… 97<br>Client/sex worker…………………………………………16 | | | | | OTHRLTN3<br>OTHRLNR3 |
| **Insert spouse's household current ID if spouse is in the study area and relationship still on going . If the spouse is not in study area, code 888/888/8888/888**<br>Spouse's Current ID**.** \|__\|__\|__\|/\|__\|__\|__\|/\|__\|__\|__\|__\|/\|__\|__\|__\| | | | | | P3CURR-ID |
| Q.52 | What are/were your partner's main occupations? | | | \|__\|__\|{OCCUP13} | |

| | | | | |
|---|---|---|---|---|
| | (Record answer(s) as given):_____________________ (OCCUPAT13)<br><br>_______________________________(OCCUPAT23)<br>**(Code up to two responses, code first mentioned occupation first.)**<br>Agriculture for home use/barter…………….01<br>Agriculture for selling…………………………02<br>Housework in own home……………... 03<br>Housekeeper (for relative or employer)…….04<br>Home brewing …………………………….. 05<br>Government/clerical/teaching …………………….. 06<br>Fishing…………………………………... 07<br>Student……………………………………………08<br>Military/police ……………………………...09<br>Shopkeeper ……………………………….. 10<br>Trading/vending ……………………….. 11<br>Bar worker or Owner…………………………...12<br>Trucker………………………………….. 13<br>Unemployed (PROBE _ NO AGRIC OR HOUSE WORK?).. 14<br>Other _______________________………………. 15<br>Medical worker (non-government)…………… ….. 16<br>Casual laborer …………………………………... 17<br>Waiter/restaurant owner……………………………. 18<br>Hair dresser/Salon owner…………………………... 19<br>Construction (brick marker, porter builder, painter, roofing).. 20<br>Mechanic (automobiles, bicycles, electronics)…….. 21<br>Boda Boda…………………………………………. 22<br>No additional response (use in 2nd field if one occupation is cited ………………………………………………. 88<br>Do Not Know ………………………………... 97<br>Client…………………………………………... 23<br>Sports betting, Gambling machine, Ludo………….. 24 NA<br>……………………………………………………………………………..98<br><br>Baking……………………………………………………………………….. 25 | \|__\|__\|{OCCUP23}<br><br>Omuntu ono yali/ akola mirimu<br>ki? oba biki by'akola/ byeyali akola ebisinga okukumalira ebiseera mu lunaku kabeere ng'afunamu/ yali afunamu ssente oba nedda?<br><br>{OCCUPS3} | | |

| | | | | | |
|---|---|---|---|---|---|
| | Mining…………………………………………………………………………….. 26<br>Textiles………………………………………………………………………. 27<br>Carpentry………………………………………………………………………… 28 | | | | |
| Q.53 | How long ago did you first have sex with this person? | Wayiseewo bbanga ki okuva lwe wasookera ddala okwegatta n'omuntu ono? | Less than 1 day code 00 days,<br>Less than 1 week code days<br>Less than one month code weeks<br>Less than one year code months<br>If 1 year or more code completed Years (If DK Code 97 in days and 98 in other boxes; 99 for NR) | \|__\|__\|<br>\|__\|__\|<br>\|__\|__\|<br>\|__\|__\| | DAYS3<br>WEEKS3<br>MONTHS3<br>YEARS3 |
| Q.54 | How long ago, did you last have sex with this person? | Wasemba ddi okwegatta naye? | Less than 1 day code 00 day<br>Less than a week code days<br>Less than a month code weeks<br>Less than one year code months<br><br>(if DK code 97 in days and 98 9n other boxes; 99 for NR) | \|__\|__\|<br>\|__\|__\|<br>\|__\|__\| | RLDYSLT3<br>RLWKSLT3<br>RLMOSLT3 |
| Q.55 | Are you still in a sexual relationship with him? | Okyegatta n'omuntu oyo? | Yes 1<br>No 2<br>DK 7 | \|__\| | RLTONGO3 |
| Q.56 | Does (was) he live (living) in this household ? | Musula (Mwali musula) wamu? | Yes 1………Q.58<br>No 2<br>DK 7 | \|__\| | RLTNH23 |
| Q.57 | Does (was) he regularly live (living) in this community? | Abeera (Yali abeera) mu kitundu kino ebiseera ebisinga? | Yes 1<br>No 2<br>DK 7 | \|__\| | RLTNCM3 |
| Q.58 | Is/was he older, younger, or about the same age? | Omuntu oyo (yali) muto kuggwe, nga mwenkana oba | Older 1<br>Younger 2<br>Same age 3….>Q.60 | \|__\| | RLTNAGE3 |

<table>
<tr><td></td><td></td><td>nga akusinga obukulu?</td><td>Do not know 7..Q.60</td><td></td><td></td></tr>
<tr><td>Q.59</td><td>About how many years [older/younger]? (Record actual # or 97=don't know)</td><td>Akusinga (Yali akusinga) oba nga omusinga emyaka emeka? Record actual # or 97=don't know)</td><td colspan="2">|__|__|</td><td>RLTNYRS3</td></tr>
<tr><td>Q.60</td><td>Have you and this partner ever used a condom?</td><td>Ggwe n'omuntu oyo, mwali mukozesezza ku bupiira (condoms)?</td><td>Yes 1<br>No 2……>Q.62<br>NR 9……Q.62</td><td>|__|</td><td>CNDEVER3</td></tr>
<tr><td>Q.61</td><td>During the most recent/ last 12 months you were having sexual relationship with this partner, how often did you use condoms?</td><td>Mu myezi ekkumi n'ebiri egisembyeyo /egyasembayo nga wegatta n'omuntu oyo, obupiira (mwali) mubukozesa buli kiseera, luusi na luusi oba temubukozesezako \temwabukozesako nakatono?</td><td>Never 1<br>Sometimes/inconsistent 2<br>Always 3<br>DK 7</td><td>|__|</td><td>RNYRCON3</td></tr>
<tr><td></td><td></td><td></td><td></td><td></td><td></td></tr>
<tr><td colspan="3"></td><td colspan="3"></td></tr>
<tr><td></td><td></td><td></td><td></td><td></td><td></td></tr>
<tr><td colspan="3">Preamble: Sometimes people enter into relationships because they need help paying for things they need, even if that is not directly exchanged for sex.</td><td colspan="3">Ebiseera ebimu abantu basalawo okwegatta mu mukwano olw’okuba nti beetaga obuyambi okusobola okugula ebintu byebaba beetaga, ne bwekiba nti tekitwaliddwa buterevu nga okwetunda oba okwegatta ofune ssente.</td></tr>
<tr><td></td><td></td><td></td><td></td><td></td><td></td></tr>
<tr><td>Q.62</td><td>Did you enter into a sexual relationship with this partner mainly in order to get things you need, money, gifts, or other things that are important to you</td><td>Wasaalawo okwegatta n’omuntu ono okusobola okufuna ebintu bye wali wetaga,ekirabo oba ebintu eby’omugaso gyoli</td><td>Yes 1<br>No 2</td><td>|__|</td><td>SEXNEEDS3</td></tr>
<tr><td colspan="6">FOURTH BLOCK</td></tr>
<tr><td colspan="3">"Can you please tell me about the sexual partner just prior to the one we just discussed?" (In the last twelve month)</td><td colspan="3">"Nandyagadde okukubuuzayo ebibuuzo bitono ebikwata ku muntu omulala gwewegatta naye mumyezi ekkumi n'ebiri egiyise eyaddibwako oyo gwetwogeddeko. (In the last twelve months)</td></tr>
</table>

| | | | | | |
|---|---|---|---|---|---|
| Q.63 | Have you had any other sexual partners in the past 12 months other than the one we just discussed? | Mu myezi ekkumi n'ebiri egiyise, wali wegasse n'omuntu omulala yenna eyaddibwako oyo gwetwogeddeko? | **Yes 1**<br>**No 2………Q.76** | \|__\| | MORE4 |
| Q.64 | Remembering the most recent time you had sex, what was your relationship to that partner at that time? | Omuntu oyo gw'osembyeyo/gwewasembayo okwegatta naye wali omuyita otya? | | \|__\|__\| | RLTN4 |
| Current husband(at the time) …………………………………… 01<br>Current consensual partner (at the time) …………………….. 02<br>Former husband/consensual partner……………………………… 03<br>Boyfriend……………………………………………………. 04<br>Occasional or casual friend……………………………………05<br>Visitor (incl. wedding/funeral) ………………………………06<br>Stranger……………………………………………………. 07<br>Workmate…………………………………………………. 08<br>Boss/work supervisor………………………………………09<br>Employee…………………………………………………. 10<br>Fellow student……………………………………………….. 11<br>Sugar Daddy ……………………………………………12<br>Relative other than spouse**(specify)**__ ___________________13<br>Other non relative **(specify)**__________________________ 14<br>Rapist (by a stranger) …………………………………… 15<br>Don't Know ……………………………………………… 97<br>Client…………………………………………16 | | | | | OTHRLTN4<br>OTHRLNR4 |
| **Insert spouse's household current ID if spouse is in the study area and relationship still on going . If the spouse is not in study area, code 888/888/8888/888**<br>Spouse's Current ID**. .** \|__\|__\|__\|/\|__\|__\|__\|/\|__\|__\|__\|__\|/\|__\|__\|__\| | | | | | P4CURR-ID |
| Q.65 | What are/were your partner's main occupations? (Record answer(s) as given):______________________ (OCCUPAT14)<br>________________________________(OCCUPAT24)<br>**(Code up to two responses, code first mentioned occupation first.)**<br>Agriculture for home use/barter…………….01<br>Agriculture for selling…………………02<br>Housework in own home……………... 03<br>Housekeeper (for relative or employer)…….04<br>Home brewing …………………………….. 05<br>Government/clerical/teaching …………………….. 06<br>Fishing……………………………………... 07<br>Student……………………………………08<br>Military/police ……………………………...09<br>Shopkeeper ………………………………... 10 | \|__\|__\|{OCCUP14}<br>\|__\|__\|{OCCUP24}<br><br>Omuntu ono yali/ akola mirimu ki? oba biki by'akola/ byeyali akola ebisinga okukumalira ebiseera mu lunaku kabeere ng'afunamu/yali afunamu ssente oba nedda? | | | |

| | | | | | |
|---|---|---|---|---|---|
| | Trading/vending …………………………. 11<br>Bar worker or Owner…………………………...12<br>Trucker………………………………….. 13<br>Unemployed (PROBE _ NO AGRIC OR HOUSE WORK?).. 14<br>Other _______________________……………… 15<br>Medical worker (non-government)………………….. 16<br>Casual laborer ……… 17<br>Waiter/restaurant owner………………. 18<br>Hair dresser/Salon owner………………... 19<br>Construction (brick marker, porter builder, painter, roofing)…………………………………………… 20<br>Mechanic (automobiles, bicycles, electronics)…21<br>Boda Boda……………………………………….. 22<br>No additional response (use in 2nd field if one occupation is cited …………………………………………………. 88<br>Do Not Know …………………………... 97<br>Client…………………………………... 23<br>Sports betting, Gambling machine, Ludo…….. 24<br>NA ……………………………………………………………………………..98<br><br>Baking…………………………………………………………………….. 25<br>Mining…………………………………………………………………….. 26<br>Textiles…………………………………………………………………….. 27<br>Carpentry……………………………………………………………………… 28 | {OCCUPS4} | | | |

| | | | | | |
|---|---|---|---|---|---|
| Q.66 | How long ago did you first have sex with this person? | Wayiseewo bbanga ki okuva lwe wasookera ddala okwegatta n'omuntu ono? | Less than 1 day code 00 day,<br>Less than 1 week code days<br>Less than one month code weeks | \|__\|__\|<br>\|__\|__\|<br>\|__\|__\|<br>\|__\|__\| | DAYS4<br>WEEKS4<br>MONTHS4<br>YEARS4 |

| | | | | | |
|---|---|---|---|---|---|
| | | | Less than one year code months<br> If 1 year or more code completed Years (If DK Code 97 in days and 98 in other boxes; 99 for NR) | | |
| Q.67 | How long ago, did you last have sex with this person? | Wasemba ddi okwegatta naye? | Less than 1 day code 00 day<br>Less than a week code days<br>Less than a month code weeks<br>Less than one year code months<br> (if DK code 97 in days and 98 9n other boxes; 99 for NR) | \|__\|__\|<br>\|__\|__\|<br>\|__\|__\| | RLDYSLT4<br>RLWKSLT4<br>RLMOSLT4 |
| Q.68 | Are you still in a sexual relationship with him? | Okyegatta n'omuntu oyo? | Yes 1<br>No 2<br>DK 7 | \|__\| | RLTONGO4 |
| Q.69 | Does (was) he live (living) in this household? | Musula (Mwali musula) wamu? | Yes 1………Q.71<br>No 2<br>DK 7 | \|__\| | RLTNH4 |
| Q.70 | Does (was) he regularly live (living) in this community? | Abeera (Yali abeera) mu kitundu kino ebiseera ebisinga? | Yes 1<br>No 2<br>DK 7 | \|__\| | RLTNCM4 |
| Q.71 | Is/was he older, younger, or about the same age? | Omuntu oyo (yali) muto kuggwe, nga mwenkana oba nga akusinga obukulu? | Older 1<br>Younger 2<br>Same age 3….>Q.73<br>Do not know 7 ..Q.73 | \|__\| | RLTNAGE4 |
| Q.72 | About how many years [older/younger]? **(Record actual # or 97=don't know)** | Akusinga (Yali akusinga) oba nga omusinga emyaka emeka? **Record actual # or 97=don't know)** | \|__\|__\| | | RLTNYRS4 |
| Q.73 | Have you and this partner ever used a condom? | Ggwe n'omuntu oyo, mwali mukozesezza ku bupiira (condoms)? | Yes 1<br>No 2……>Q.75<br>NR 9……Q.75 | \|__\| | CNDEVER4 |
| Q.74 | During the most recent/ last 12 months you were having sexual relationship with this partner, how often did you use condoms? | Mu myezi ekkumi n'ebiri egisembyeyo /egyasembayo nga wegatta n'omuntu oyo, obupiira (mwali) mubukozesa buli kiseera, luusi na luusi oba | Never 1<br>Sometimes/inconsistent 2<br> Always 3<br>DK 7 | \|__\| | RNYRCON4 |

| | | | | | |
|---|---|---|---|---|---|
| | | temubukozesezako \temwabukozesako nakatono? | | | |
| | | | | | |
| | | | | | |
| | | | | | |
| Preamble: Sometimes people enter into relationships because they need help paying for things they need, even if that is not directly exchanged for sex. | | | **Ebiseera ebimu abantu basalawo okwegatta mu mukwano olw'okuba nti beetaga obuyambi okusobola okugula ebintu byebaba beetaga, ne bwekiba nti tekitwaliddwa buterevu nga okwetunda oba okwegatta ofune ssente.** | | |
| | | | | | |
| Q.75 | *Did you enter into a sexual relationship with this partner mainly in order to get things you need, money, gifts, or other things that are important to you* | *Wasaalawo okwegatta n'omuntu ono okusobola okufuna ebintu bye wali wetaga,ekirabo oba ebintu eby'omugaso gyoli* | Yes 1<br>No 2 | \|__\| | SEXNEEDS4 |

| | | | |
|---|---|---|---|
| **END OF REPETITIVE SEXUAL BLOCK<br>IF NOT MARRIED AND NO SEX IN LAST 12 MONTHS SKIP TO Q.77** | | | |
| **Disagreements on some issues can occur between men and women, which sometimes result into violence. i would like to ask you some questions on violence.** | **Mu nkolagana ez'omukwano wakati w'abaami n'abakyala obutakkaanya ku bintu ebimu butera okubaawo, emirundi egimu buvaamu obusambattuko. nandyagadde okukubuuzaayo ebibuuzo ku busambattuko.** | | |
| Q76. In the past 12 months did any of your partners: **PROMPTED** | Mu myezi ekkumi n'ebiri egiyise, omuntu yena gwe wegatta naye yali akukozeeko ebintu bino? [PROMPT | | |
| | | Yes No NA | |
| Verbally abuse or shout at you? | Okukuvuma/okukuwogganira / Okukuboggolera | 1 2 8 | PABUSEYR |
| Push, slap, punch, kick, drag or otherwise physically hurt you? | Okukusindika/ okkukukuba oluyi /okukukuba ekikonde/okukusamba/okukusikambura/ oba okukulumya ku mubiri? | 1 2 8 | PSPKDYR |
| | | | |
| Forced you to have sex when you did not want to? | Okukukaka okwegatta nga toyagala. | 1 2 8 | OTHFSXPY |
| Other | Ekirala | 1 2 8 | POTHVYR |
| specify | | | OTHPVYRS |

| | | | | | |
|---|---|---|---|---|---|
| | **I am now going to ask you some questions about hiv testing. Please know that whatever answers you provide will be kept strictly confidential** | **Ngenda kukubuuzaayo ebibuuzo ebikwata ku kwekebeeza akawuka ka siriimu.Byonotudda mu byonna bijja kukumibwa nga byakyama.** | | | |
| Q.77 | Have you ever received your HIV results from anywhere? | Wali ofunyeko ebyava mu musaayi gwo ebikwata ku kawuka ka siriimu okuva awantu wonna? | Yes 1<br>No 2---->**Q.** 83<br>NR 9----->**Q.** 83<br>NA(Never tested) 8-->**Q.** 83 | \|__\| | RHIVEVER |
| | | | | | |
| Q.78 | How long ago did you last receive your last HIV results? | Wayisewo banga ki okuva lwewasembayo okufuna ebyava mu kukebeera omusaayi ebikwata ku kawuka ka siriimu? | code in days, weeks, months,<br>years if completed years code 00 in the rest of the boxes if less than a week,month and year code 98<br>(If DK Code 97 in days and 98 in other boxes; 99 for NR) | \|__\|__\|days<br>\|__\|__\|weeks<br>\|__\|__\|months<br>\|__\|__\|years | HIVPERIODD<br>HIVPERIOD W<br>HIVPERIOD M<br>HIVPERIODY |
| Q.80 | What was the result of this last HIV test? | Ebyava mu musaayi gwo ebisembyeyo byali bitya? | Negative 1____Q.83<br>Positive 2<br>Indeterminate 3____Q.83<br>No Response 9<br>Don't know/Don't remember 7____Q.83 | \|__\| | HIVRSLT |
| Q.81 | When was your first positive HIV test? Please give month and year if you can. | Ddi lwebasokeraddala okukebera nebakizuula/bakugamba nti olina akawuuka akaleeta siriimu? Please give month and year if you can. | Month<br>Year<br>DK/NR 9997 | \|__\|__\|<br>\|__\|__\|__\|__\| | FHIVTESTM<br>FHIVTESTY |
| Q.82 | I When was your last negative HIV test? This would be your last negative before you tested positive. Please give month and year. | Basemba ddi okukukebera n'osangibwa nga tolina kawuka akaleeta siriimu?Guno guyinza okuba nga gwe murundi gwewasembayo okwekebeza no sangibwa nga tolina | Month<br>Year<br>DK/NR 9997<br>9998 first time they tested, they were HIV<br>Yes 1 If yes why?<br>No 2<br>NR 9 | \|__\|__\|<br>\|__\|__\|__\|__\| | LHIVTSTNM<br>LHIVTSTNY |

| | | | | | |
|---|---|---|---|---|---|
| | | kawuka ka siriimu Please give month and year. | positive or were born with HIV | | |
| Q.83 | In the past 12 months, have you tried to get an HIV test at a health care facility but were refused? | Mu myezi ekumi nebbiri egiyise, ogenzeeko ewakebererwa okugezako okwekebeza akawuuka akaleeta sirimu mu musaayi ne bagana okukukebera? | Yes 1<br>No 2<br>NR 9 | \|__\| | THIVTSTR |
| | | | | | |
| Q.84 | Have you ever been contacted by partner notification services to get HIV tested? | wali otukiriddwako omuntu yenna nga akulaga obwetavu bwo kwekebeza akawuka akaleta silimu mu musaayi okugeza nga omusawo okukubira essimu oba okukutukirira mu buntu oba omwagalwa wo okukikutegezaako? | Yes 1<br>No 2------------->**Q.86**<br>NR 9------->**Q.86** | \|__\| | APN |
| Q.85 | Did you get HIV tested because you were contacted by partner notification services ? | Wekebeza akawuka akaleeta silimu mu musaayi olwokuba omusawo oba omwagalwa wo yakukubira essimu oba okukutukirira mu buntu? | Yes 1<br>No 2<br>NR 9<br>Already on ART 3 | \|__\| | APNTEST |
| Q.86 | Have you ever used PreP? | Wali omizeeko ku ddagala erikendeza ku katyabaga akokukwatibwa akawuuka ka siriimu PrEP? | Yes 1<br>No 2-------------Q.88<br>DK/NR 7 | \|__\| | PREPEVER |
| Q.87 | Are you currently using PreP? | Mukiseera kino omira eddagala erikendeza ku katyabaga ko kukwatibwa akawuka ka siriimu PreP? | Yes 1<br>No 2<br>NR 7 | \|__\| | PREPUSE |

| # | |
|---|---|
| **FOR PERSONS WHO ARE HIV+, (I.E. RESPONSE TO Q.80 WITH 2=POSITIVE), ASK Q.89 ELSE SKIP TO 98** | |
| **We want to ask you about use of health care services and long term medication. This information will be strictly** | **Nandyagadde okukubuzayo ebibuuzo ebikwata ku ngeri abantu gyebakozesamu obujjanjabi ne ku ddagala erimiribwa abantu okumala ebbanga** |

| | | | | | |
|---|---|---|---|---|---|
| confidential. you do not have to answer any of these questions. | | | egwanvu. By'ononziramu byonna bijja kukumibwa nga byakyama. Osobola okuddamu ebibuuzo ebimu ebirala n'obireka. | | |
| Q.89 | Have you ever been to a clinic to receive care for HIV? | Wali obaddeko/ogenzeko mu kifo kyona ew'ajjanjabirwa okufuna edaggala erikendeeza obungi bw'akawuka ka sirimu mu musaayi (ARVS)? | Yes 1<br>No 2-----skip to Qn. 98<br>DK 7___Q.98<br>NR 9____Q.98 | \|__\| | HIVCARE2 |
| Q.90 | Have you ever been on ARVs? | Omira/wali omizeeko eddagala erikendeeza obungi bw'akawuka ka siriimu mu musaayi (ARVS)? | Yes 1<br>No 2_____Q.98<br>DK 7_____Q.98<br>NR 9_____Q.98 | \|__\| | ARVMED |
| Q.91 | Are you currently taking ARVs? | Eddagala lino erikendeeza obungi bw'akawuka ka siriimu mu musaayi olimira kati (ARVS)? | Yes 1<br>No 2<br>DK 7<br>NR 9 | \|__\| | CUARVMED |
| Q.92a | What is /was your current source of ARVs? **{Codes from the table below}**<br><br>**PATIENT ID: ________**<br><br>**CARD SEEN**<br>Yes …..1<br>No........2 | Eddagala lino erikendeeza akawuka ka siriimu mu musaayi (ARVS) otera/wateranga kulifuna kuva wa? | \|__\|__\|__\|<br>Other arv current source<br>________________________<br><br>Other clinic type for current medication<br>Gov't 1<br>NGO 2<br>Private 3<br>\|__\| | | ARVSOURC2<br>OTHARVSOU1<br>ARVTYPE1 |

**IF YES TO Q.89 PLEASE ASK**

**Q93a. In the past year, did you change the clinic where you receive HIV care?**

**Mu myezi ekumi n'ebbiri egise okyusizako eddwaliro w'ofunira eddagala erikendeza obungi bw'akawuka ka siriimu mu musaayi?**

- Yes
- No
- Unsure/don't remember
- Refused

**Q93b. What motivated you to change the clinic where you receive HIV care? Kiki ekyakusikiriza okukyusa eddwaliro w'ofunira eddagala erikendeza obungi bw'akawuka ka siriimu mu musaayi?**

[*SELECT ALL THAT APPLY*]

- I didn't want to be seen or recognized when accessing services
- Clinic was too far away
- Clinic was too crowded or busy
- I relocated to a new community
- I was mistreated by staff or providers
- I thought I could get better care somewhere else
- Clinic closed or shut down
- Other, specify: ________

Q93c. Were you issued a transfer form before you switched to the new facility? **Wafuna ebaluwa eyakukiriza okukyusa eddwaliro w'ofunira eddagala erikendeza obungi bw'akawuka ka siriimu mu musaayi okugenda ku ddwaliro eddala?**

- Yes (*formal transfer*)
- No (*self-transfer*)
  - Unsure/don't remember
  - Refused

Q94. Did you disclose your HIV status or ARV use history when you enrolled in the new facility? **Bwewagenda okufuna eddagala erikendeza obungi bw'akawuka ka siriimu mu musaayi ewapya wabategeza nti omira eddagala erikendeza akawuka akaleeta obulwadde bwa siriimu?**

- Disclosed both HIV status and ARV use history
- Disclosed only HIV status (not ARV use history)
- Did not disclose HIV status (retested for HIV)
- Unsure/don't remember
  - Disclosed both HIV status and ARV use history
  - Disclosed only HIV status (not ARV use history)
  - Did not disclose HIV status (retested for HIV)
  - Unsure/don't remember

**"People sometimes forget to take their ARVs daily.**
**Olw'olumu abantu berabira okumira eddagala lyabwe erikendeza obungi bw'akawuka ka siriimu mu musaayi buli luna**
**Q.95 In the past 30 days, how many days have you missed taking any ARV pills?"Mu naku amakumi assaatu eziyise naku meka zewerabira okumira eddagala lyo erikendeza akawuka ka siriimu mu musaayi? Response should be 0-30.**

| | | | | |
|---|---|---|---|---|
| Q.96 | How long ago did you first start ART medications? | Wayiseewo bbanga ki okuva lwe watandika okumira eddagala erikendeza obungi bw'akawuka ka siriimu mu musaayi? | ARTDAYS (0-6 acceptable responses)<br><br>ARTWKS<br><br>(1-3 acceptable responses) | \|__\|__\|{artdays}<br><br>\|__\|__\|{artwks} |

| | | | | |
|---|---|---|---|---|
| | | | ARTMOS (1-11 acceptable responses)<br><br>ARTYRS (1 – 20 acceptable responses)<br><br>**## Code 97 for Don’t know in ART days** | \|__\|__\|{artmos}<br><br>\|__\|__\|{artyrs} |
| | | | | |
| 97 | Through which of the following service delivery models do you currently receive your ARVs?<br><br>Ku bino wammanga ngeriki gy’ofunamu eddagala lyo erikendeeza obungi bwa akawuka ka siriimu mu musaayi(ARVs) | Pick-up at a clinic or hospital after interacting with a clinician (FBIM) 01<br><br>Participate in an adherence support group where we receive our ARVs at the facility (FBG) 02<br><br>Pick-up at a clinic or hospital directly at the pharmacy *without* interacting with a clinician or nurse (FTDR) 03<br><br>Direct delivery by a neighbor, family member, facility peer, or other provider (CCLAD) 04<br><br>Pick-up at a pop-up space set up by providers or peers in the community (CDDP) 05<br><br>Pick-up at a private or retail pharmacy in the community 06<br><br>Other, specify: _____ | Ndifuna okuva mu dwaliro ng’omusaawo amaze okundaba oba okunzijjanjaba (FBIM) 01<br><br>Ndifuna okuva ku ddwaliro nga maze okufuna okubuddabuddibwa okwawamu ku kumira eddagala lyange obulungi (FBG) 02<br><br>Ndifuna okuva mu dwaliro buterevu ku dirisa webagabira eddagala nga simaze kwogera namusaawo yena ajjanjaba (FTDR) 03<br><br>Abantu ab’enjawulo balindetera buterevu okugeza balirwana bange,ab’enganda zange mikwano gyange oba abagaba eddagala abalala (CCLAD) 04<br><br>Ndifuna okuva mu kitundu okiba kitekeddwawo abasaawo mu kitundu wetusobola okufunira eddagala oba ku balwadde banange 05<br><br>Ndifuna okuva webatunda eddagala mu kitundu mwembera pharmacy 06<br><br>Other, specify: _____ | |

**HEALTH UNITS CODES FOR RAKAI DISTRICT 2008 TO DATE**

| NAME OF HEALTH UNIT | CODE | OWNERSHIP |
|---|---|---|
| Baale Gunda | 037 | GOV'T |
| Bakka | 104 | GOV'T |
| Bethrehem | 010 | GOV'T |
| Bikira | 002 | NGO |
| Bitabago | 047 | PRIVATE |

| | | |
|---|---|---|
| Bugona | 099 | GOV'T |
| Bukeeri HCIII | 123 | GOV'T |
| Bulamu | 100 | GOV'T |
| Butembe | 078 | GOV'T |
| Butiti | 098 | GOV'T |
| Buyamba Dwaniro | 028 | GOV'T |
| Buyamba NGO | 050 | NGO |
| Buyiisa | 072 | GOV'T |
| Buziranduulu | 015 | GOV'T |
| Byakabanda | 076 | GOV'T |
| Byerima | 095 | GOV'T |
| Gayaaza | 005 | GOV'T |
| Gwanda | 085 | GOV'T |
| Heal the nation | 044 | NGO |
| Kabira | 012 | GOV'T |
| Kabusota | 070 | GOV'T |
| Kabuwoko H/C | 006 | GOV'T |
| Kabuwoko NGO | 007 | NGO |
| Kacheera | 031 | GOV'T |
| Kagamba | 027 | GOV'T |
| Kakundi | 033 | GOV'T |
| Kakuuto | 016 | GOV'T |
| Kaleere | 034 | GOV'T |
| Kalisizo H/C | 087 | NGO |
| Kalisizo T.C/Hosp | 001 | GOV'T |
| Kalisizo/Kyango | 014 | GOV'T |
| Kamulegu H/C III | 120 | GOV’T |
| Kasaali | 004 | GOV'T |
| Kasankala | 055 | GOV'T |
| Kasankala NGO | 059 | NGO |
| Kasasa | 023 | GOV'T |
| Kasensero | 089 | GOV'T |
| Katatenga | 049 | GOV'T |
| Kayanja | 063 | GOV'T |
| Kayanja Prison | 045 | GOV'T |
| Kayayumbe | 048 | NGO |
| Kayonza | 035 | GOV'T |
| Kayonza Ddwaniro | 103 | GOV'T |
| Kibaale H/C II | 030 | GOV'T |
| Kibaale Home | 067 | NGO |
| Kibanda | 022 | GOV'T |
| Kibanda Clinic | 068 | PRIVATE |
| Kibuuka | 011 | GOV'T |
| Kifamba | 021 | GOV'T |
| Kijjeja | 086 | GOV'T |
| Kijonjo | 097 | GOV'T |
| Kimuli | 041 | GOV'T |
| Kirumba | 101 | GOV'T |
| Kiziba | 051 | GOV'T |
| Kitovu Hospital | 118 | Private |

| | | |
|---|---|---|
| Kitovu Mobile | 119 | NGO |
| Kyabigondo | 057 | GOV'T |
| Kyakanyomozi | 079 | GOV'T |
| Kyakonda | 074 | GOV'T |
| Kyakuwa | 092 | GOV'T |
| Kyalulangira | 065 | GOV'T |
| Kyanamukaaka HC IV | 121 | GOV'T |
| Kyebe | 018 | GOV'T |
| Kyempewo | 102 | GOV'T |
| Kyotera G/C | 071 | PRIVATE |
| Kyotera M/C | 080 | PRIVATE |
| Kyotera Moslems | 075 | NGO |
| Kyotera T.C/Mitukula | 024 | GOV'T |
| Lukerere | 061 | GOV'T |
| Lwabakooba | 056 | GOV'T |
| Lwakaloolo | 054 | GOV'T |
| Lwamaggwa NGO | 069 | NGO |
| Lwamaggwwa | 029 | GOV'T |
| Lwamba | 036 | GOV'T |
| Lwanda | 025 | GOV'T |
| Lwankoni | 042 | GOV'T |
| Lwembajjo | 052 | GOV'T |
| Lwensinga | 060 | GOV'T |
| Lyantonde Hospital | 122 | GOV'T |
| Magabi | 082 | GOV'T |
| Mayanja | 090 | GOV'T |
| Mbuye | 026 | NGO |
| Micungiro | 038 | GOV'T |
| Minziiro | 040 | GOV'T |
| Mirigwe | 094 | GOV'T |
| Mukisa | 064 | PRIVATE |
| Mutukula | 017 | GOV'T |
| Muzito | 081 | PRIVATE |
| Nabigasa | 009 | GOV'T |
| Nabyajjwe | 062 | GOV'T |
| Nakasoga | 084 | GOV'T |
| Nakatoogo | 008 | GOV'T |
| Nangoma | 020 | GOV'T |
| Nazareth | 088 | NGO |
| Ndolo | 039 | GOV'T |
| Nkenge | 053 | GOV'T |
| No additional Response | 888 | |
| Not applicable | 998 | |
| Others | 115 | |
| Rakai Program Kalisizo Hub | 105 | NGO |
| Rakai hospital | 032 | GOV'T |
| Sanje Dom | 046 | PRIVATE |
| Sserulunda | 013 | GOV'T |
| St. Bernard Mannya | 093 | NGO |
| St. Joseph | 083 | PRIVATE |

| | | |
|---|---|---|
| St. Martine | 043 | PRIVATE |
| St.Gyaviira | 058 | PRIVATE |
| St.Mugagga | 073 | PRIVATE |
| Taso Masaka | 117 | NGO |
| Uganda Cares Masaka | 116 | NGO |

| # | | | | |
|---|---|---|---|---|
| **International Physical Activity Questionnaire** | | | | |
| **Think about all the vigorous activities that you did in the last 7 days. Vigorous physical activities refer to activities that take hard physical effort and make you breathe much harder than normal, like heavy lifting, digging, vigorous exercise, fetching water, or fast bicycling. Think only about those physical activities that you did for at least 10 minutes at a time.** | | | **Lowooza ku bintu byokoze nga byeetaaga amanyi mu nnaku omusanvu eziyise. Ebintu ebikolebwa namanyi byebyo ebyetaaga okuteekamu amaanyi amangi** okugeza okusitula ebizitowa, okulima,okukyima amazzi oba okuvuga akagaali mu bwangu **nebikuleetera okussa enyo okusinga kumbeera eyabulijo. Lowooza kwebyo byokka ebyakolebwa wakiri mu ddakiika kkumi(10minutes) omulundi ggumu.** | |
| Q.98 | During the last 7 days, on how many days did you do vigorous physical activities? | Mu nnaku omusanvu eziyise, ennaku mmeka z'okoze ebintu ebyetaaga amanyi amanji | \|__\| days [range 0-7] per week<br>If 0, skip to Q.100 | IPAQ1 |
| Q.99 | How much time did you usually spend doing vigorous physical activities on one of those days? | Watwalangabudde ki ngokola ebintu ebyetaaga amanyi amangi mu lunaku olumu kwezo? | \|__\|__\| hours per day<br>\|__\|__\| minutes per day<br>97 Don't know/Not sure | IPAQ2h<br>IPAQ2m |
| **Think about all the moderate activities that you did in the last 7 days. Moderate activities refer to activities that take moderate physical effort and make you breathe somewhat harder than normal, like carrying light loads, bicycling at a regular pace, sweeping, or moderate exercise? Do not include walking. Think only about those physical activities that you did for at least 10 minutes at a time.** | | | ***Munnaku omusanvu eziyise, Lowooza ku bintu byokoze nga byetaaga amaanyi agekigero. Ebintu ebikolebwa namaanyi agekigero byebyo ebyetaaga okuteekamu amaanyi agekigero nebikuleetera okussa namaanyi amangiko okusinga ku mbeera eyabulijo okugeza okusutula ebintu ebiwewuka ebitali bizito, okuvuga eggali mu mpolampola, okweera. Okutambula tokubaliramu/tokutwaliramu Lowooza kwebyo byokka ebyakolebwa wakiri mu ddakika kumi(10minutes) omulundi gumu.*** | |
| Q.100 | During the last 7 days, on how many days did you do moderate physical activities?. | Mu nnaku musanvu eziyise, ennaku mmeka zokozemu ebintu ebyetaaga amaanyi agekigero? | \|__\| days<br>[range 0-7] per week<br>If 0, skip to Q.102 | IPAQ3 |

| | | | | |
|---|---|---|---|---|
| Q.101 | How much time did you usually spend doing moderate physical activities on one of those days? | Watwalanga budde ki nga okola ebintu ebyetaaga amanyi agekigero mulunaku olumu kwezo? | \|__\|__\| hours per day<br>\|__\|__\| minutes per day<br>97 Don't know/Not sure | IPAQ4h<br>IPAQ4m |
| Think about the time you spent walking in the last 7 days. This includes at work and at home, walking to travel from place to place, and any other walking that you have done solely for recreation, sport, exercise, or leisure. | | | Munnaku omusanvu eziyise, lowooza ku budde bwewamala ng'otambula. Muno mulimu ng'oli kumulimu, awaka, okutambula okuva mukifo ekimu okudda mukirala, n'okukola ebyemizanyo, okukola dduyiro mu budde bwo obweddembe. | |
| Q.102 | During the last 7 days, on how many days did you walk for at least 10 minutes at a time? | Mu nnaku omusanvu eziyisse, naku mekka zotambudde wakiri eddakiika kumi omulundi gumu nga toyimiriddemu? | \|__\| days [range 0-7] per week<br>**If 0, SKIP TO Q.105** | IPAQ5 |
| Q.103 | How much time did you usually spend walking on one of those days? | Watwalanga budde ki ngotambula mu lunaku olumu kwezo? | \|__\|__\| hours per day<br>\|__\|__\| minutes per day<br>97 Don't know/Not sure | IPAQ6h<br>IPAQ6m |

**Nandyagadde okukubuuzayo ebibuuzo ebikwata ku kufumba.**

| # | | | | | |
|---|---|---|---|---|---|
| **Cooking Practices** | | | | | |
| | | | | | |
| Q.105 | Does your household primarily cook indoors or outdoors?? | Mu maka mwobeera, musinga kufumbira munda oba wabweru? | Indoors…….1<br>Outdoors……2 | \|__\| | COOKPRAC4 |
| Q.106 | Are you the primary person responsible for cooking in your household? | Mumaka mwobera, gwe avunanyizibwa ku kufumba? | Yes…………1<br>No…………2 | \|__\| | COOKPRAC5 |
| **DIET** | | | | | |
| **The next questions ask about foods and drinks. I have cards here that shows you some examples. As you answer** | | | **Ebibuuzo ebiddako bikwata ku bibala n'enva endiirwa zotera okulya.<br>Nina ekipande ekiraga ebimu kubika byebibala nenva endiirwa ebiiri mukitundu. Buli kifananyi** | | |

| | | | | |
|---|---|---|---|---|
| **these questions please think of a typical week in the last year.** | | | **ekiraga ebintu ebyandibadde biribwa, ngoddamu ebibuuzo bino mwattu lowooza ku wiiki emu mu myezi ekkumi nebiri egiyise?** | |
| Q.107 | In a typical week, on how many days do you eat fruit? (SHOW CARD) | Okutwalira awamu mu wiiki, nnaku mmeka z'olyamu ebibala (show card) | \|__\|__\| number of days [range 0-7] | FRUIT |
| Q.108 | In a typical week, on how many days do you eat vegetables? (SHOW CARD) | Okutwalira awamu mu wiiki, nnaku mekka z'olyamu enva endiirwa? (show card) | \|__\|__\| number of days [range 0-7] | VEG |
| | | | | |
| Q.109 | In a typical week, on how many days do you eat processed food, e.g. packaged snacks? (SHOW CARD) | Okutwalira awamu mu wiiki, nnaku mmeka z'olyamu eby'okulya ebikoleddwawo/obumpankyumpankyu | \|__\|__\| number of days [range 0-7] | SNACKS |
| Q.110 | In a typical week, on how many days do you eat food that has been fried in oil or fat, e.g. mandazi, chapati, rolex, fried meats and fish? (SHOW CARD) | Okutwalira awamu mu wiiki, nnaku mmeka z'olyamu ebintu ebisikiddwa mu butto oba omuzigo okugeza mandazi, chapati ,eby'enyanja ebisiike, enyama ensiike | \|__\|__\| number of days [range 0-7] | FRIEDFOOD |
| Q.111 | In a typical week, on how many days do you drink sugar-sweetened beverages, e.g. sodas, pre-packed juices, sweetened fruit drinks? (SHOW CARD) | Okutwalira awamu mu wiiki, nnaku mmeka z'onywamu eby'okunywa ebiri mu sukali, soda oba juice omupakingire ddala | \|__\|__\| number of days [range 0-7] | BEVERAGE |

| # | | | | | |
|---|---|---|---|---|---|
| **With the next questions, we would like to learn more about salt in your diet.** | | | **Ebibuuzo ebiddako twagala okwongera okumanya ebikwata ku mmunyo mubyolya** | | |
| Q.112 | How often do you add salt, salty seasoning or a salty sauce to your prepared foods | Emirundi gyenkanawa gyoyongera omunyo oba gyoyongera ebirungo ebirimu omunyo mu | Always………1<br>Often…………2<br>Sometimes…3<br>Rarely………4<br>Never………5<br>Don't know…7 | Buli kiseera 1<br>Ebiseera ebisinga obungi 2<br>Luusi na luusi 3<br>Tekitera kubaawo 4 | \|__\|SALT |

| | | | | | |
|---|---|---|---|---|---|
| | | byokulya ebimaze okufumbibwa? | | Tekibererawo ddala 5<br>Don't know 7 | |
| **Smoking** | | | | | |
| Q.113 | Have you ever smoked any of the following tobacco products? | Wali onyweddeko kubintu bino wammanga nga biva mu taaba? | Yes No<br>Cigarettes(Sigala) 1 2<br>Taaba 1 2<br>Pipes(Emindi) 1 2<br><br>**If all are 2, then skip to Q.117.** | | CIGAEVER<br>TABAEVER<br>PIPEEVER |
| Q.114 | Do you currently smoke any of the following tobacco products? | Mukiseera kino onywa ku bintu bino wammanga nga biva mu taaba? | Yes No<br>Cigarettes(Sigala) 1 2<br>Taaba 1 2<br>Pipes(Emindi) 1 2 | | CIGACUR<br>TABACUR<br>PIPECUR |
| Q.115 | If smoked cigarette ask else skip to Q. 116<br><br>On a typical day, when you smoke (or smoked), how many cigarettes do you smoke? | Mulunaku olumu lwonywa oba lwewabanga onywedde sigala onywa/wanywanga eminwe emmeka? | \|__\|__\| | | SMOKENUM |
| Q.116 | How many years total have you smoked? | Emyaka emeka nga ogigasse gyonyweredde/gye wanywera sigala , taaba oba emindi? | \|__\|\|__\| **If less than a year code 96** | | SMOKEYRS |
| **Alcohol** | | | | | |

| | | | | | |
|---|---|---|---|---|---|
| Q.117 | How often do you have a drink containing alcohol? | Emirundi gyenkanawa gyonywa ekintu ekirimu omwenge?(prompted) | Never 1<br>Monthly or less 2<br>2-4 times a month 3<br>2-3 times a week 4<br>4 or more times a week 5 | ***Never ( no alcohol in the past 12 months) [skip to Q.120 ]*** \|__\|<br>Sinywa ngako oba sinyweddeko mumyezi 12 egiyise….. 1<br>omulundi gumu mu mwezi oba obutaweera…2<br>emirundi 2 okutuka kwe 4 mu mwezi…. 3<br>emirundi 2 okutuka kwe'essatu mu wiiki…. 4<br>emirundi 4 oba okusingawo mu wiiki…5 | ETOH1 |
| Q.118 | How many standard drinks containing alcohol do you have on a typical day? (show card) | Olunaku lwoba onywedde omwenge byakunywa bimeka ebirimu ebitamiza byonywa?( prompted) | 1 or 2… 1<br>3 to 4……2<br>5 to 6…….3<br>7 to 9…….4<br>10 or more…5 | \|__\| | ETOH2 |
| Q.119 | How often do you have six or more drinks on one occasion? | Emirundi gyenkanawa gyofuna eby'okunywa mukaaga oba okusingawo omulundi gumu. | Never 1<br>Daily or almost daily 2<br>Weekly 3<br>Monthly 4<br>Less than monthly 5 | Tekibangawo 1 \|__\|<br>Buli lunaku oba kumpi buli lunaku 2<br>Wakiri omulundi gumu mu wiiki 3<br>Wakiri omulundi gumu mu mwezi 4<br>Emyezi egimu mu mwaka naye si buli mwezi 5 | ETOH3 |
| # | | | | | |

**Quality of Life**

| | | | | |
|---|---|---|---|---|
| Under each heading, please tick the ONE box that best describes your health TODAY. | | Kubino byengenda okukusomera njagala ombulire ekisinga okunyonyola embeera y'obulamu bwo bweri olwalero. | | |
| Q.120 | O I have no problems walking about 1.<br>O I have some problems in walking about 2.<br>O I am confined to bed 3. | Sirina buzibu mukutambula tambula ….1<br>Ninamu ko obuzibu obutonotono mu kutambula tambula ……. 2<br>Sisobolera ddala kutambula……3 | \|__\| | QOLWALK |
| Q.121 | O I have no problems washing or dressing myself 1.<br>O I have some problems washing or dressing myself 2.<br>O I am unable to wash or dress myself 3. | Sirina buzibu mukwenaaza oba okweyambaza 1<br>Ninamu ko obuzibu obutonotono mukwenaaza oba okweyambaza 2<br>Sisobolera ddala kwenaaza oba okweyambaza 3 | \|__\| | QOLDRESS |
| Q.122 | O I have no problems doing my usual activities 1.<br>O I have some problems doing my usual activities 2.<br>O I am unable to do my usual activities 3. | Sirina buzibu mu kukola mirimu gyange egyabulijjo 1<br>Ninamu ko obuzibu butonotono mu kukola emirimu gyange egyabulijjo 2<br>Sisobolera ddala kukola mirimu gyange egyabulijjo 3 | \|__\| | QOLACT |
| Q.123 | O I have no pain or discomfort 1.<br>O I have moderate pain or discomfort 2.<br>O I am in extreme pain or discomfort 3. | Sirina wannuma oba okuwulira obubi 1<br>Ninamu ko obulumi oba okuwulira obubi kutonotono 2<br>Nina obulumi bwamaanyi nnyo oba mpulira bubi nnyo nnyo 3 | \|__\| | QOLPAIN |
| Q.124 | O I am not anxious and depressed 1.<br>O I am moderately anxious and depressed 2.<br>O I am extremely anxious and depressed 3. | Siri mweralikirivu era siri mwenyamivvu 1<br>Ninamu ko obweralikirivvu era n'obwenyamivvu butonotono 2<br>Nina obwerarikirivvu n'obwenyamivvu bwamaanyi nnyo 3 | \|__\| | QOLANX |
| Q.125 | We would like to know how good or bad your health is TODAY.<br>-This scale is numbered from 0 to 100.<br>-100 means the best health you can imagine.<br>0 means the worst health you can imagine.<br>-Mark an X on the scale to indicate how your health is TODAY.<br>-Now, please write the number you marked on the scale in the box below. | Twandyagadde okumanya embeera y'obulamu bwo bweri kati? nnungi oba mbi *(provide a scale)*<br>Akapimo kano katekeddwako ennamba okuva ku zeero paka ku kikumi (0-100)<br>-kikumi (100) kitegeeza obulamu obulungi enyo bwosobola okulowooza ko<br>-zeero (0) kitegeeza obulamu obubi enyo bwoyinza okulowooza ko<br>-Ng'okozesa akapimo kano laga obulamu bwo bwebuli olwaleero<br>-kati wandiika ennamba gy'olambye kukapimo kano mu ka boxi wammanga | \|__\|_<br>_\|__\| | QOL100 |

**TB HISTORY/SYMPTOMS**

| | | | | | |
|---|---|---|---|---|---|
| Q.126 | Have you ever been diagnosed with TB before? | Wali okebedwako n'ozulibwa ng'olina obulwadde bwa kafuba (TB)? | Yes………1<br>No………2<br>skip to Qn 128<br>DK/NR….-→Q.128 | \|__\| | TBDX |
| Q.127a | Have you ever been treated for TB before? | Wali ofunyeeko obujanjabi bw'obulwadde bwakafuba (TB)? | Yes………1<br>No…………2<br>DK/NR……7 | \|__\| | TBTX |
| Q.127b | If yes, how long ago (months/years) was your most recent diagnosis?<br>○<br>• | Wayisewo bbanga ki Okuva lwebasembayo Okeberebwa n'ozulibwa ng'olina obulwadde bwa kafuba (TB)?M | | \|__\|__\|DD<br>\|__\|__\|WW<br>\|__\|__\|MM<br>\|__\|__YY | |

| | | | | | |
|---|---|---|---|---|---|
| Q. 127c<br>Q. 127d<br>Q. 127e | Have you ever been treated for TB?<br>If yes are you currently being treated for TB? If no to above how long ago (months/years) did you complete you most recent course of treatment? | Wali ofunyeeko obujanjabi bw'obulwadde bwakafuba (TB)?<br>Mukiseera kino ofuna obujanjabi bw'obulwadde bwakafuba (TB)?<br>Wayisewo bbanga ki ng'omazeyo obujanjabi bw'obulwadde bwakafuba (TB) obusembyeyo? | Yes………1<br>No…………2<br>DK/NR……7<br>Yes………1<br>No…………2<br>DK/NR……7_<br>\|__\|__\|DD<br>\|__\|__\|WW<br>\|__\|__\|MM<br>\|__\|__\|YY | ☐ | TBTX |

| | | | | | |
|---|---|---|---|---|---|
| Q.127f | Do you currently have a cough? | okolola | Yes 1<br>No 2 | \|__\| | |
| Q.128 | If yes for how long have you been coughing? | Okololedde banga ki? | \|__\|__\|DD<br>\|__\|__\|WW<br>\|__\|__\|MM<br>\|__\|__\|YY | | |
| Q.129 | Do you have a fever? (I would defer to the Rakai team as to how best to | Oyokya | Yes 1<br>No 2 | | |

| | | | | | |
|---|---|---|---|---|---|
| | word this, if "fever" is not well understood.) | | | | |
| Q. 13 0 | In the past three months, have you lost weight - either 5kg or enough weight to make your clothes feel loose? | Mu myezi essatu egiyise okoze nga kino kiweza kiro 5 oba engoye okulebera | Yes 1<br>No 2 | \|__\| | |
| Q.131a | In the past week, have you had drenching sweats at night, enough to make you feel a need to change your clothes? | Mu naku omusanvu eziyise ofunyeko okutuyana ekiro okwamanyi nga walina okukyusa engoye | Yes 1<br>No 2 | \|___\| | |

**History of NCDs and Health Care Utilization**

| | | | | | |
|---|---|---|---|---|---|
| Q.131b | Before today, have you ever been told by a doctor or other health worker that you have raised blood pressure or hypertension? | Wali otegezeddwako Dokita oba omusawo omutendeke nti olina obulwadde bwa pulesa? | Yes…………1<br>No…………2---→132<br>DK/NR……7--->132 | \|__\| | HTNTOLD |
| Q.131c | In the past two weeks, have you taken any drugs (medication) for raised blood pressure prescribed by a doctor or other health worker? | Munnaku kkumi nanya (14) eziyise omizeeko ku ddagala lyonna erijjanjaba pulesa nga Dokita oba omusawo omutendeke yenna yalikuwandiikidde? ***(to consult)*** | Yes…………1<br>No……………2-skip to Q.131<br>DK/NR……7-skip to Q.131 | \|__\| | HTNMED |
| 0 | | | | | |
| | | | | | |

| | | | | | |
|---|---|---|---|---|---|
| Q.131d | Are you currently taking any herbal or traditional remedy for your raised blood pressure? | Mukiseera kino okozesa eddagala lyekinnansi okukujjanjaba obulwadde bwa pulesa? | Yes……………1<br>No…………2<br>DK/NR………7 | \|__\| | HTNTRAD |
| Q.132 | Have you ever been told by a doctor or other health worker that you have raised blood sugar or diabetes? | Wali otegezeddwako Dokita oba omusawo omutendeke nti olina obulwadde bwa sukaali oba sukali mungi mu musaayi gwo? | Yes…………1<br>No……………2___-skip to 136<br>DK/NR……7--→Q.135 | \|__\| | DIABTOLD |
| Q.133 | In the past two weeks, have you taken any drugs (medication) for diabetes prescribed by a doctor or other health worker? | Munnaku kumi nannya(14) eziyise omizeeko/okozesezaa ko eddagala erijjanjaba sukaali nga Dokita oba omusawo omutendeke yalikuwandikidde? | Yes……………1<br>No…………2-skip to Q.135<br>DK/NR………7-skip to Q135 | __\| | DIABMED |
| | | | | | |
| | | | | | |
| Q.134 | Are you currently taking any herbal or traditional remedy for your diabetes? | Mukiseera kino okozesa eddagala ly'ekinnansi okukujjanjaba obulwadde bwa sukaali? | Yes……………1<br>No……………2<br>DK/NR………7 | \|__\| | DIABTRAD |
| Q.135 | Have you ever been told by a doctor or other health worker that you have had a heart attack? | **Wali otegezeddwako Dokita oba omusawo omutendeke nti olina obulwadde bwo** okulumwa mukifuba nga kizze mubwangu nga kireteddwa obulwadde bw'omutima | Yes……………1<br>No……………2<br>DK/NR………7 | \|__\| | HEARTTOLD |
| Q. 136 | Have you ever been told by a doctor or other health worker that you have had a stroke? | **Wali otegezeddwako Dokita oba omusawo omutendeke nti olina obulwadde bwo** okulumwa mukifuba nga kizze mubwangu nga kireteddwa obulwadde | | | |

| | | | | | |
|---|---|---|---|---|---|
| | | bw'okusanyalala oludda olumu olw'omubiri oba okusanyalala omubiri gwona? | | | |
| Q.137 | Have you ever been told by a doctor or other health worker that you have a respiratory condition such as asthma ? | **Wali otegezeddwako Dokita oba omusawo omutendeke nti olina obulwadde bwa asthma /oluyiro?** | Yes……………1<br>No……2-→Q.140<br>DK/NR…7→Q.140 | \|__\| | ASTHMATOLD |
| Q.138 | Are you currently on any medicine for a respiratory condition such as asthma or(chronic Obstructive pulmonary disease (COPD)? | **Mukiseera kino olina eddagala lyona ly'okozesa okwejjanjaba Asthma/oluyiro?** | Yes……………1<br>No……………2-skip to Q,140<br>DK/NR………7-skip to Q.140 | \|__\| | ASTHMAMED |
| | | | | | |
| | | | | | |
| Q.140 | How many times have you visited a doctor or health care worker in the past 12 months? | Mumyezi ekumi n'ebbiri egiyise emirundi emmeka gyogenzeeko okulaba omusawo? | \|__\|__\|<br><br>If 0, skip to Q.142<br>DK……97 | | HEALTHNUM |
| Q.141 | For what health condition(s) did you visit the doctor or health care worker(s) in the past 12 months? (Select all that apply, unprompted) | Mumyezi ekumi n'ebbiri egiyise buzibuki oba bulwaddeki bwewalina obwakuleetera okugenda okulaba omusawo? | Yes No<br>HIV 1 2<br>Hypertension 1 2<br>HeartDisease 1 2<br>Diabetes 1 2<br>Respiratory Condition (Asthma, COPD) 1 2<br>Malaria 1 2<br>STI 1 2<br>Pregnancy 1 2<br>Injury 1 2<br>Diarrhea 1 2<br>Fever 1 2<br>Other\|__________ 1 2 | \|__\|<br>\|__\|<br>\|__\|<br>\|__\|<br>\|__\|<br>\|__\|<br><br>\|__\|<br><br>\|__\|<br>\|__\|<br>\|__\|<br>\|__\| | HIV12M<br>HTN12M<br>HEART12M<br>DIAB12M<br>COPD12M<br>MALA12M<br>STI12M<br>PREG12M<br>INJURY12M<br>DIARRH12M<br>FEVER12M<br>OTH12M<br>OTH12MSP |
| **Asthma and Chronic Bronchitis** | | | | | |

| | | | | |
|---|---|---|---|---|
| Q.142 Have you had any wheezing in the past 12 months? | Mumyezi ekumi n'ebbiri egiyise, ofunyeko obukaaba mukifuba? | Yes……………1<br>No……………2<br>DK/NR………7 | \|__\| | ASTHMA |
| Q.143 Have you had any productive cough (with phlegmn) for 3 consecutive months, 2 years in a row? | Ofunyeko okukololola nga ekikolondorwa kivaayo okumala emyezi esatu egiddiringana mu bbanga ery'emyaka ebiri? | Yes……………1<br>No……………2<br>DK/NR………7 | \|__\| | BRONCHITIS |

| | | | | | | |
|---|---|---|---|---|---|---|
| **If ever interviewed in R20 and not a vicinity participant skip to Q.194** | | | **ST. GEORGE'S RESPIRATORY QUESTIONAIRE (SGRQ)** | | | |
| ***This questionnaire is designed to help us learn much more about your breathing and how it affects your life.*** | | | | **Ebibuuzo bino bikoleddwa okutuyamba okumanya engeri okussakwo gyekukutataganya n'engeri gyekukosamu obulamu bwo. .** | | |
| **Q.144** | ***Would you describe your current health as*** | Wandigambye nti embeera y'obulamu bwo nungi nnyo , | Very good …1<br>Good …2<br>Fair ….3 | Nnungi nnyo..1<br>Nnungi ….  2<br>Bwetyo bwetyo.. 3 | \|__\| | **SG1** |

| | | | | | | |
|---|---|---|---|---|---|---|
| | ***Very good, good, fair, poor, or very poor?*** | nungi, BwetyoBwetyo, mbi, mbi nnyo. | Poor….4<br>Very poor….5 | Mbi …. 4<br>Mbi nnyo… 5 | | |
| **Questions about how much chest trouble you have had over the past 03 months** | | | Ebibuuzo bino bikwata kungeri gyotataganyiziddwa mu kifuba mu banga eryemyezi esaatu egiyise. | | | |
| Q.145 | over the past 3 months, I have coughed | Mu myezi esaatu egiyise, okolodde | **Most days a week..1**<br>**Several days a week…2**<br>**A few days a month..3**<br>**Only with chest infections..4**<br>**Not at all..5** | Ennaku ezisinga mu wiiki… 1<br>Ennaku eziwerako mu wiiki …2<br>Ennaku ntono mu mwezi… 3<br>Obulwadde bubadde mukifuba mwoka….4<br>Tekibaddewo nakatono… 5 | \|__\| | **SG2** |
| Q.146 | Over the past 3 months, I have brought up phlegm (sputum) | Mu myezi esaatu egiyise okolodde n'ovaamu ekikolondolwa: | **Most days a week..1**<br>**Several days a week..2**<br>**A few days a month…3**<br>**Only with chest infections…4**<br>**Not at all….5** | Ennaku ezisinga mu wiiki…1<br>Ennaku eziwerako mu wiiki…. 2<br>Ennaku ntono mu mwezi…. 3<br>Obulwadde bubadde mukifuba mwoka …..4<br>Tekibaddewo nakatono…. 5 | \|__\| | **SG3** |
| Q.147 | Over the past 3 months, I have had shortness of breath | Mu myezi esaatu egiyise, obadde n'obuzibu mu kussa: | **Most days a week…1**<br>**Several days a week…2**<br>**A few days a month…3**<br>**Only with chest infections….4**<br>**Not at all…..5** | Ennaku ezisinga mu wiiki … 1<br>Ennaku eziwerako mu wiiki… 2<br>Ennaku ntono mu mwezi…. 3<br>Obulwadde bubadde | \|__\| | **SG4** |

| | | | | | | |
|---|---|---|---|---|---|---|
| | | | | mukifuba mwoka …..4<br>Tekibaddewo nakatono…..5 | | |
| Q.148 | Over the past 3 months, I have had attacks of wheezing | Mu myezi esaatu egiyise ofunyeko obukaaba mukifuba ng'ossa: | **Most days a week…1**<br>**Several days a week…2**<br>**A few days a month…3**<br>**Only with chest infections….4**<br>**Not at all…..5** | Ennaku ezisinga mu wiiki…. 1<br>Ennaku eziwerako mu wiiki …..2<br>Ennaku ntono mu mwezi… 3<br>Obulwadde bubadde mukifuba mwoka …..4<br>Tekibaddewo nakatono…. 5 | \|__\| | **SG5** |
| Q.149 | **During the past 3 months how many severe or very unpleasant attacks of chest trouble have you had? *Please tick in one*** | Mubanga ery'emyezi esaatu egiyise emirundi emeka gy'ofunyeko obuzibu obwamanyi mukifuba? | More than 3 attacks…1<br>3 attacks…2<br>2 attacks….3<br>1 attack…4<br>No attacks….5 | Emirundi egissuka mwe'saatu…. 1<br>Emirundi esaatu… 2<br>Emirundi ebiiri..3<br>Omulundi gumu…4<br>Tokifunyeko… 5____Q.151 | \|__\| | **SG6** |
| Q.150 | **How long did the worst attack of chest trouble last? *Please tick in one* (go to SECTION 7 if you had no severe attacks)** | Obuzibu mukifuba obwasinga okuba obwamaanyi bwatwala banga ki? (Go to SECTION 7 if you had no severe attacks) | A week or more 1.<br>3 or more days 2<br>1 or 2 days<br>Less than a day 4 | Wiiki namba oba okusingawo… 1<br>Ennaku ssatu oba okusingawo… 2<br>Olunaku lumu oba bbiri….. 3<br>Olunaku telwawera…. 4 | \|__\| | **SG7** |
| Q.151 | **Over the past 3 months, in an average week, how many good days (with little chest** | Mu myezi essatu egiyise, okutwalirawamu mu wiiki, nnaku mmeka zobadde n'obuzibu mukifuba obutono? | No good days 1<br>1 or 2 good days 2<br>3 or 4 good days 3<br>Nearly every day is good 4 | Tewabaddewo lunaku lulungi..1<br>Olunaku lumu oba bbiri… 2<br>Ennaku ssatu oba nnya… 3 | \|__\| | **SG8** |

| | | | | | | |
|---|---|---|---|---|---|---|
| | **trouble) have you had?** ***Please tick in one*** | | Every day is good 5 | Kyenkana buli lunaku luba lulungi …4<br>Buli lunaku luba lulungi…. 5 | | |
| Q.152 | **If you have a wheeze, is it worse in the morning?** ***Please tick in one*** | Bwofuna obukaaba mu kifuba nga ossa, buyitirira/bweyongera mubudde obw'okumakya? | Yes 1<br>No 2 | \|__\| | **SG9** | |
| Section 1 | | | | | | |
| Q.153 | **How would you describe your chest condition? (please tick one)** | Oyinza kunyonyola otya embeera y'ekifuba kyo? | The most important problem I have 1<br>Causes me quite a lot of problems 2<br>Causes me a few problems 3<br>Causes no problem 4 | Kyekizibu kyosinga okubera nakyo..1 \|__\|<br>Kikuleetera obuzibu bwamaanyi … 2<br>Kikuleetera obuzibu butono…. 3<br>Tewali buzibu bwekikuleetera … 4 | \|__\|<br>SGA1 | |
| Q.155 | If you have ever had paid employment | Bwoba nga wali obaddeko nomulimu ogukusasuza ssente z'omusaala | My chest trouble made me stop work ..1<br>My chest trouble interferes with my work or made me change my work..2<br>My chest trouble does not affect my work…3 | Obuzibu bwekifuba bwakuletera okulekerawo okukola ….. 1<br>Obuzibu bwekifuba bwakutataganya mu mirimugyo oba bwakuletera okukyusa omulimo gwo…2<br>Obuzibu bwekifuba kyo tebukosa mirimo gyo…..3 | \|__\|SGA3 | |
| **SECTION 2** | | | | | | |
| **Questions about what activities usually make you feel breathless these days (please tick in each box that applies to you these days)** | | | | Ebibuuzo ebikwata ku bintu byokola ebikuletera okuwulira ngolina obuzibu mu kussa/okuziyira ennaku zino | | |

| | | | Nsaba onziremu oba kitufu oba sikitufu ku bino byengenda okukusomera | | |
|---|---|---|---|---|---|
| | | | **True (Kituufu)** | **False (Sikituufu)** | |
| Q.156 | Sitting or lying still | Okutuula oba okwebaka awamu nga tovaawo | **1** | **2** | **SGB1** |
| Q.157 | Getting washed or dressed | Okunaaba oba okwambala | **1** | **2** | **SGB2** |
| Q.159 | Walking around the home | Okutambulatambulirako awaka | **1** | **2** | **SGB3B** |
| Q.158 | Walking outside on the level | Okutambulira ku museetwe oba awaterevu | **1** | **2** | **SGB3** |
| Q.160 | Walking up a flight of stairs | Okulinnya amaddaala | **1** | **2** | **SGB4** |
| Q.161 | Walking up hills | Okulinnya akasozi | **1** | **2** | **SGB5** |
| Q.162 | Playing sports or games (eg football, netball, Ludo, etc) | Okuzanya obuzanyo/ emizanyo (okugeza okubaka omupiira, okusamba omupiira, Ludo) | **1** | **2** | **SGB6** |
| **SECTION 3** | | | | | |
| | Some more questions about your cough and breathlessness these days. | Ebibuuzo bino ebirala bikwata kukukolola n'obuzibu mukussa/okuziyiira ennaku zino. Nsaba onziremu oba kitufu oba sikitufu ku bino byengenda okukusomera | **True (Kituufu)** | **False (Sikituufu)** | |
| Q.163 | My cough hurts | Okukolola kukukosa /kukulumya | **1** | **2** | **SGC1** |
| Q.164 | My cough makes me tired | Okukolola kukukooya | **1** | **2** | **SGC2** |
| Q.165a | I am breathless when I talk | Ofuna obuzibu mu kussa /okuziyiira ngoyogera | **1** | **2** | **SGC3** |
| Q.165b | I am breathless when I bend over | Oziyira oba ofuna obuzibu mu kusa ng'okutamyeko | **1** | **2** | **SGC3B** |
| Q.166 | My cough or breathing disturbs my sleep | Okukolola oba engeri gyossamu kutataganya okwebaka kwo | **1** | **2** | **SGC4** |

| | | | | | |
|---|---|---|---|---|---|
| Q.167 | I get exhausted easily | Okoowa mangu | **1** | **2** | **SGC5** |
| **SECTION 4** | | | | | |
| | Questions about other effects that your chest troubles may have on you these days. | Ebibuuzo bino bikwata ku bintu ebirala ebiyinza okuba nga bivudde ku buzibu bwekifuba munnaku zino<br><br>Nsaba onziremu oba kitufu oba sikitufu ku bino byengenda okukusomera | **True (kituufu)** | **False (Sikituufu)** | |
| Q.168 | My cough or breathing is embarrassing in public | Okukolola oba obuzibu mu kussa kukuswaza ng'olimu bantu | **1** | **2** | **SGD1** |
| Q.169 | My chest trouble is nuisance to my family, friends or neighbours | Obuzibu mukifuba kizibu eri abengandazo, mikwanogyo oba abemirirwano | **1** | **2** | **SGD2** |
| Q.170a | I get afraid or panic when I cannot get my breath | Otya bwoba tosobola kussa | **1** | **2** | **SGD3** |
| Q.170b | I feel that I am not in control of my chest problem | mpulira nga obulwadde bw'ekifuuba kyange sibusobola | **1** | **2** | **SGD3B** |
| Q.171 | I do not expect my chest to get any better | Tosuubira kifuba kyo kutereera | **1** | **2** | **SGD4** |
| Q.172 | I have become frail or an invalid because of my chest | Onafuye oba ofuuse owokwegendereza olw'ekifuba kyo | **1** | **2** | **SGD5** |
| Q.173 | Exercise is not safe for me | Okukola dduyiro kyabulabe gyoli | **1** | **2** | **SGD6** |
| Q.174 | Everything seems too much of an effort | Buli Kintu kyona kyokola kirabika nga ekyetaaga okusaamu amaanyi amangi | **1** | **2** | **SGD7** |
| **SECTION 5**<br>**Q. 175a**<br>**Probe to see if respondent is taking any medication for chest problem** Yes 1 \|__\|<br>If not receiving medication go straight to Section 6 No 2<br>N/A (no chest problem) 8 | | | | | **CHESTPROB** |

| | | | | | |
|---|---|---|---|---|---|
| | **Question about medication if you are receiving no medication go straight to section.** | Ebibuuzo bino bikwata ku ddagala lyo lyomira/okozesa olw'obuzibu bw'ekifuba<br><br>Nsaba onziremu oba kitufu oba sikitufu ku bino byengenda okukusomera | **True (Kitufu)** | **False (Sikitufu)** | |
| Q.175b | My medication does not help me very much | Eddagala lyo terilina nnyo kyerikuyamba | **1** | **2** | **SGE1** |
| Q.176 | I get embarrassed using my medication in public | Oswala okukozesa eddagala lyo nga oli mu bantu | **1** | **2** | **SGE2** |
| Q.177 | I have unpleasant side effects from my medication | Ofuna obuzibu nga buva ku ddagala lyo | **1** | **2** | **SGE3** |
| Q.178 | My medication interferes with my life a lot | Eddagala lyo litataganya nyo obulamu bwo | **1** | **2** | **SGE4** |
| **SECTION 6** | | | | | |
| | **These are questions about how your activities might be effected by your breathing.** | Ebibuuzo bino bikwata kungeri ebintu byokola gyebiyinza okuba nga bikoseddwa olwengeri gyossamu<br>Nsaba onziremu oba kitufu oba sikitufu ku bino byengenda okukusomera | **True (Kitufu)** | **False (Sikitufu)** | |
| Q.179 | I take long time to get washed or dressed | Otwala obudde buwanvu okunaaba oba okwambala | **1** | **2** | **SGF1** |
| Q.180 | I cannot take a bath or shower, or I take a long time | Tosobola kunaaba oba otwala obudde buwanvu ng'onaaba | **1** | **2** | **SGF2** |
| Q.181 | I walk slower than other people, or I stop for rests | Otambula mpola bwogeragerанya ku bantu abalala oba oyimiriramu okuwumulamu bw'oba otambula | **1** | **2** | **SGF3** |

| | | | | | |
|---|---|---|---|---|---|
| Q.182 | Jobs such as housework take a long time, or I have to stop for rest | Emirimo j'ewaka gikutwalira obudde buwanvu oba Ojiyimirizamu oluvanyuma nogenda mumaaso okujikola | **1** | **2** | **SGF4** |
| Q.183 | If I walk up one flight of stairs, I have to go slowly or stop | Bwoba otambula ngolinya amaddala okutuuka kumwaliiro ogudako/kunyumba eddako oba olina okugenda empola oba okuyimiriramu. | **1** | **2** | **SGF5** |
| Q.184 | If I hurry or walk fast, I have to stop or slow down | Bwo'tambula ngoyanguwa, oba oyina okuyimiriramu oba okukendeezako | **1** | **2** | **SGF6** |
| Q.185 | My breathing makes difficult to do things such as walk up hills, carrying things Upstairs, Light gardening such as weeding, dance, Play bowl or play golf | Engeri gyossamu ekifula kizibu okukola ebintu okugeza okutambula ng'olinya akasozi, okwambusa ebintu waggulu mu nyumba eyakalina, okulima okutonotono okugeza okukoola, okuzinamu/okubibya, oba okuzanya golf | **1** | **2** | **SGF7** |
| Q.186 | My breathing makes it difficult to do things such as heavy loads, dig the garden Shovel snow, jog or walk at 5 miles per hour, play tennis or swim | Engeri gyossamu ekifula kizibu okukola ebintu okugeza okusitula ebizito, okulima, okudduka oba okutambula mayilo taano(5miles) buli saawa, okuzanya tennis oba okuwuga | **1** | **2** | **SGF9** |
| | | | | | |
| Q.187 | My breathing makes it difficult to do things such as very heavy manual work, Run, cycle swim fast or | Engeri gyossamu ekifula kizibu okukola ebintu okugeza okukola ebintu ebyeetaaga amanyi amangi, okudduka, okuvuga eggali, okuwuga mubwangu oba okuzanya emizanyo egyokuvuganya | **1** | **2** | **SGF11** |

| | | | | | |
|---|---|---|---|---|---|
| | play competitive sport | | | | |
| **SECTION 7** | | | | | |
| | We would like to know how your chest usually affects your daily life. | Twandyagadde okumanya engeri ekifuba kyo gyekitera okukosa embeera y'obulamu bwo eyabulijjo Nsaba onziremu oba kitufu oba sikitufu ku bino byengenda okukusomera | **True (KITUFU)** | **False (Sikitufu)** | |
| Q.188 | I cannot play sports or games | Tosobola kuzanya mizanyo | **1** | **2** | **SGG1** |
| Q.189 | I cannot go out for entertainment or recreation | Tosobola kugenda kwesanyusamu | **1** | **2** | **SGG2** |
| Q.190 | I cannot go out of the house to do the shopping | Tosobola kuva munyumba kugenda kugula bintu kumadduuka | **1** | **2** | **SGG3** |
| Q.191 | I cannot do housework | Tosobola kukola mirimo gyawaka | **1** | **2** | **SGG4** |
| Q.192 | I cannot move far from my bed or chair | Tosobola ku genda wala kuva ku kubuliri bwo oba mu ntebe | **1** | **2** | **SGG5** |

| | | | | |
|---|---|---|---|---|
| | **Here is a list of other activities that your chest troubles may prevent you doing. (You do not have to tick these, they are just to remind of way in which your breathlessness may affect you):**<br>Going for walk or walk the dog<br>Doing things at home or in the garden<br>Sexual intercourse<br>Going out to church, pub, or place of entertainment<br>Going out in bad weather or into smoky rooms<br>Visiting family or friend or playing with children | Bino wammanga bye bintu ebirala ebiyinza okukulema okukola olw'obuzibu bwolina mukifuba. Ebintu bino bikujjukizamu engeri obuzibu mukussa gyebuyinza okukossamu.<br>Okugenda okutambula oba okutambuza embwa<br>Okukola ebintu awaka oba okugenda okulima<br>Okwegatta | Okugenda mu sinzizo, mu baala, ekiddula oba ebifo ebisanyukirwamu<br>Okugenda ebweru mumbeera y'obudde embi oba mubisenge omuli omuka<br>Okukyalira ab'engandazo oba mikwanogyo oba okuzanya n'abaana | |

| | | | |
|---|---|---|---|
| | **Now would you tick in the box (one only) which you think best describes how your chest affects you.** | Kati njagala ombulire ku bino, kiki kyolowooza ekisinga okunyonyola obulungi engeri ekifuba gyekikukossamu | |
| Q.193 | It does not stop me doing anything I would like to do 1<br><br>It stops me doing one or two things I would like to do 2<br>It stops me doing most of the things I would like to do 3<br>It stops me doing everything I would like to do 4 | Tekikulemesa kukola kintu kyona kyewandyagadde okukola 1<br>Kikulemesa okukola ekintu kimu oba bibiri byewandyagadde okukola<br>2 Kikulemesa okukola ebintu ebisinga byewandyagadde okukola<br>3<br>Kikulemesa okukola ebintu byona byewandyagadde okukola 4 | SGEND |

| | | | | | |
|---|---|---|---|---|---|
| **THE PEPFAR PROGAM EVALUATION MODULE (DREAMS)**<br>**FOR WOMEN 15-32 YEARS PLEASE ASK ELSE SKIP TO 211** | | | | | |
| Q.194 | Have you ever been enrolled in the DREAMS program? | Wali wenyigiddeko mu ntekateeka ezikwata ku bawala n'abakyala abato eziri mu Program gyebayita DREAMS? | Yes 1 If yes provide **DREAMS ID:** _______<br>No 2__Q.197<br>NR 9-__Q.197 | \|__\| | EVERDREAM |
| | | | | | |
| | | | | | |
| | | | | | |
| | | | | | |
| | | | | | |
| | | | | | |
| | | | | | |
| | | | | | |
| | | | | | |
| | | | | | |
| | | | | | |
| | | | | | |
| | | | | | |
| | | | | | |
| | | | | | |
| Q.195 | Did you graduate from DREAMS? | Wattikirwa mu DREAMS | Yes ……..1<br>No ………2---Q.200 | | GRADUATE |
| Q.196 | Which year did you graduate? | Wattikirwa mwaka ki? | | \|__\|__\|__\|__\| | GRAD_YR |

## Migration and Mobility Module

| | | | | | |
|---|---|---|---|---|---|
| Q.197 | **In the past 12 months, have you spent any nights away from your current residence (?** | **Mu myezi kumi n'ebbiri egiyise osuzeeko mukifo ekirala ekitali kyobeeramu kati**? | Yes No<br>1 2<br>**If 2 skip to measurements** | \|___\| | NIGHTWAY |
| | | | | | |
| | | | | | |
| | | | | | |

Q.198 In the past 12 months, how many nights did you spend in Kampala?

**Mu myezi kumi n'ebbiri egiyise biro bimeka by'osuzeeko e Kampala?**

|___|___|DDNWKLA
|___|___|WWNWKLA

|__|__|MMNWKLA

Q.199 In the past 12 months, how many nights did you spend in Masaka?

**Mu myezi kumi n'ebbiri egiyise biro bimeka by'osuzeeko e Masaka?**

|__|__|DDNWMSK
|__|__|WWNWMSK
|__|__|MMNWMSK

**PREGNANCY QUESTIONNAIRE FOR ALL WOMEN**
**Nandyagadde okukakasiza ddala oba oli lubuto oba nedda. N'olwekyo, kankankase nga nziramu okkubuuza ku bino wammanga.**

1. Oli lubuto kati? {PREGNOW}

   Yes 1: |__| (**VISIBLE END, NOT VISIBLE GO TO 2**)
   No 2: |__|
   Don't know 7: |__|

2. Wasemba ddi okugenda munsonga? **(UNPROMPTED)** **{LMP}**

   Within the last 30 days 1: |__| {GO TO END}
   More than 1 month ago ***NOT on Depo*/Norplant** 2: |__| {Offer Preg Test}
   More than 1 month ago ***ON Depo/Norplant*** 4: |__| {GO TO END}
   Never had a period/Menopause/Just delivered 3: |__| {GO TO END}

3. **OFFER PREGNANCY TEST.**

   Accept Test? Yes 1: |__| {ACCEPTST}
   No 2: |__| {**END**}

4. **ADMINISTER PREGNANCY TEST.**

   Is she pregnant? Yes 1: |__| {PREGTEST}
   No 2: |__| {GO TO END}

**---------------->******(END ACTIVITIES******<---------------**

| COMPUTER ID | LABNO | ___/____/___/______ |
| --- | --- | --- |

**TIME ENDED** **|__|__|:|__|__|am/pm END_TIME}**
Editor #|__|__|__|{EDITOR} QC# |__|__|__|{**QC**}

Data entry clerk #|__|__|__| {DATACLERK} {DE DATE } ____/___/___{dd mm yyyy}

**PARTNER IDENTIFICATION**

| PLACE COMP_ID HERE | PLACE LABNO HERE |
|---|---|
| | |

**Int_date** **___/___/_____**
**(dd mm yyyy)**
VISIT# |**R|22**|{VISITNO}
**Current ID**
Super Cluster# |__|__|__| {REGION }
Community# |__|__|__| {COMM_NUM }
HH # |__|__|__|__|__| {HH_NUM }
Member # |__|__|__| {MEMBER _NUM}

**INTERVIEWER: FOR ALL RESPONDENTS IN MARRIAGE OR CONSENSUAL UNION TRY TO OBTAIN INFORMATION THAT WILL ALLOW US TO LINK THE INDIVIDUAL TO HER MARRIAGE OR CONSENSUAL PARTNERS WITHIN THE SAME HOUSEHOLD OR WITHIN THE SAME COMMUNITY. PAY ATTENTION TO CASES OF POLYGAMY WHERE WIVES MAY BE LIVING WITHIN ONE HOUSE OR IN DIFFERENT HOUSEHOLDS. USE ADDITIONAL PAGES IF >3 PARTNERS. FOR SAME HOUSEHOLD PARTNERS, TRY TO FIND THE MEMBER # FROM THE HOUSEHOLD CENSUS LISTING. FOR PARTNERS**

**IN DIFFERENT HOUSEHOLDS, THIS INFORMATION WILL HAVE TO BE COMPLETED LATER.**

**Partner 1** **BLOCK # |__|**{**BLOCKNO1**}

Nandyagadde ntegeere bulungi, olina omwami gw'osula naye mu maka muno?

**(If yes,)** Amanya ge y'ani? Mbuulira amannya gonna gebatera okumuyita.

______________________________________________________________________

Current ID # for partner 1: |___|____|__|/|___|___|___|/|__|___|__|__/|__|__|__|

(Supercluster/Community / Household /Member)

**(If no,)**Atera kubeera mu kitundu kino? Yes ... 1 No... 2 Out spouse....3

Erinya lye y'ani?

______________________________________________________________________

**EDITOR: TRY TO LOCATE ID FROM HOUSEHOLD CENSUSES IN COMMUNITY**

**Current ID# for partner1:** |___|____|__|/|___|___|___|/|__|___|__|__/|__|__|__|

(Supercluster / Community / Household / Member)

**(If not)** Osobola okumbulira wa gy'abeera?

______________________________________________________________________

______________________________________________________________________

| **SPECIMENS COLLECTED** | | | |
|---|---|---|---|
| **Purple Top** | **PT Result Req.** | **Blood** | **RT Result Req.** |
| Yes …….1<br>No …......2<br>NA ….....8<br>Re-bled..11 | Single………….........1<br>No............................2<br>Couple-Married ........3<br>Couple non-marital...4<br>AKP……………….5<br>NA…….…………...8 | Yes …..….1<br>No …........2<br>NA ..…......8<br>Re-bled….11 | Single .......................1<br>No.............................2<br>Couple-Married …….3<br>Couple-non-marital ...4<br>AKP……………...….5<br>NA………….....…....8 |
| Rebled Date: ____/____/______ | | Rebled Date: ____/____/______ | |
