# Supplementary material for: Seroprevalence of Rift Valley fever virus and factors associated with exposure in south-central Uganda
Source: BMC Infect Dis. 2026 Jul 7;26:1751. doi: 10.1186/s12879-026-13923-1 (PMC13628802; doi:10.1186/s12879-026-13923-1)
Supplement: Supplementary file 2 [file 12879_2026_13923_MOESM2_ESM.pdf]

RAKAI COMMUNITY COHORT STUDY (RCCS) ROUND 21 FOLLOW UP MALE LUGANDA QUESTIONNAIRE ver 4, 4th June 2025

#			
	INTERVIEWER	|__|__|__|__|__|	FIELD_WRKR
	PLACE COMP_ID HERE	**PLACE LABNO HERE**	STUDY_ID
	INT DATE	___/___/20__ dd mm yyyy	INT_DATE
	VISIT #	**|R|2|2|**	VISITNO
	**Current ID**		CURR_ID
	Super Cluster #	|__|__|__|__|	REGION
	Community #	|__|__|__|__|	COMM_NUM
	HH #	|__|__|__|__|__|	HH_NUM
	Member #	|__|__|__|__|	MEMBER_NUM
	SEX	**M**	SEX
	NATIONAL ID CARD NO (NIN):	|__|__|__|__|__|__|__|__|__|__|__|__|__|__|__|	NATION_ID

**MOBILITY:**	No change ……..….1	Move within community.....................2	In migrant...……............…………
		Had out-migrated now back in the same Household..	
Other (specify)...........9 ___________________________		Had out-migrated now back in the different Household..10<br>Vicinity……………………………………………………………………………………….…………………………12	

PHOTO
Seen …………1 No............…...2 Taken......………..3 Taken/check album………....4

CONSENT
RCCS Baseline ……..………….1 RCCS Follow-up ……………....2

ADDITIONAL STUDY CONSENT
Agreed………….……………...1 Disagreed ……………..…...…..2

STORED SAMPLES CONSENT
With Study_ID...........................1 Without Study_ID......................2 Not for future studies..................3

GENETIC TESTING CONSENT

Agreed with Study ID………......1
Agreed without Study ID…......…3
Disagreed ……………..……...…..2

VIRAL/BACTERIAL GEN. CONSENT

Agreed with Study ID……....…1 Agreed without Study ID…...….3 Disagreed ………….……...…...2

INT LOCATION

Hub 1 Home 2 RHSP 3
Kampala 4 Masaka 5 Other 6

RAPID TEST

Yes.………………….............1
Refused 2
Seen at home……………..…3

CHPIE

Yes CHPIE...1
No CHPIE... 2

ART INITIATED

Yes ……….........1
No ………..…..2
Declined…......3
NA..………......8

**PHONE CONTACT:**	**C/O:**
Personal…...........1<br>Not Personal……2<br>NA……………...8	________________________<br>**Phone #:** _____________________

ANTHROPOMETRIC

Weight (Kgs) |__|__||__|: |__|__|	Weight2 (Kgs) |__|__||__|: |__|__|	Weight3 (Kgs) |__|__||__|: |__|__|
Height (cm) |__|__||__|: |__|__|	Height2 (cm) |__|__||__|: |__|__|	Height3 (cm) |__|__||__|: |__|__|
Waist (cm) |__|__||__|: |__|__|	Waist2 (cm) |__|__||__|: |__|__|	Waist3 (cm) |__|__||__|: |__|__|
Hip (cm) |__|__||__|: |__|__|	Hip2 (cm) |__|__||__|: |__|__|	Hip3 (cm) |__|__||__|: |__|__|

BLOOD PRESSURE

BP (mmHg) ___/____ BP2 (mmHg) ___/___ BP3 (mmHg) ___/___

	**Names:**			
	Usually called	Erinnya lyo gwe ani lyebatera okukuyita		COMM_NAME1
	Religious names	Erinnya ely'eddiini		COMM_NAME2
	Other names	Amannya amalala		COMM_NAME3
	Mother's maiden name:	Mother's maiden name:		NAME_MOT
Q.1 a	How old are you? **(Age in completed years.) (Valid range 15 and above**	Olina emyaka emeka?	|__|__|	AGEYRS
	RECORD CONFIRMED AGE		|__|__|	CONF_AGE

Q.2					

	Q.2b How long ago were you las interviewed by any study done by RHSP/**(If period less than a year STOP, clarify on the likely study and/or consult QC and editors).**	Wayiseewo bbanga ki okuva abasawo ba Rakai Program lwebasemba/lwebasembyeyo okukulabako mu kunoonyereza kwonna okukolebwa ekitongole kya Rakai program ng'ogyeko okujjanjabibwa mu bulwaliro. **(If period less than a year STOP, clarify on the likely study and/or consult QCs and editors).**		|__|__| <br>|__|__| |__|__| |__|__|	Days<br><br>weeks<br>Months<br>{MONTHLI}<br><br>Years<br>|{YEARLI}
Q.3	Have you ever gone to school?	Wali osomyeko?**)**	Yes…1<br>No….2 …..>Q.8	|__|	EDUCATE

Q.4	To what level? **(Probe if got additional training)** **(Code highest level)**	Wakomawa? **(probe if got additional training)** (**Code highest level)**	P1…………01<br>P2…………02<br>P3…………03<br>P4…………04<br>P5…………05<br>P6…………06<br>P7…………07<br>S1…………09<br>S2…………10<br>S3…………11<br>S4…………12<br>S5…………13<br>S6…………14<br>Technical/University……………15<br>Primary professional………<br>……16<br>O'level professional ……………17<br>Primary or O'level apprenticeship……18<br>A'level apprenticeship ……………… 19	|__|__	EDUCYRS

#					
**FOR THOSE 15-24 years please ask Q.5 else skip to Q.8**					
Q.5	Are you currently enrolled in school?	Mu kiseera kino oli mu ssomero?	Yes 1---->**Q.8**<br>No 2	|__|	SCHOOL
Q.6	At what age did you leave school?	Walina emyaka emeka we wavira mu musomero?		|___|	LAGESCH
Note: Code Yes in Q.5 applies for primary, secondary or university/Tertiary, not to vocational /apprenticeship or informal training programs. If not currently enrolled or left school					

Q.7	what was the main reason you left school	Nsonga ki enkulu eyakuleteera okuva mu ssomero	**Yes**	No	
a.	Financial	Tewaali ssente	1	2	FINSCH
b.	Impregnated	Wafunyisa omuwala olubuto	1	2	PREGSCH
c.	Moved	Wava mu kifo ekimu n'ongenda mu kifo ekirala	1	2	MOVESCH
d.	Sexual harassment in or on the way to school	Wakakibwa okwegatta ng'oli ku somero oba ng'ogenda ku ssomero	1	2	HARASCH
e.	Bullying on the way or into school	Okutulugunyizibwa oba okutisibwatisibwa ng'ogenda oba nga oli ku somero	1	2	BULLWSCH
f	Parent died	Omuzadde yafa	1	2	PARESCH
g	Accomplished my educational goals	Wafuna/watuukiriza ebirubirirwa byo eby'okusoma.	1	2	ACCOMSCH
h	Left school because of COVID-19	Bulwadde bwa COVID-19	1	2	COVIDSCH
i	Other		1	2	OTHSCH
	Specify				SPESCH

**Q.8**	What kind of work do you do, or what kind of activities keep you busy during an average day, whether you get money for them or not?	Okola mirimu ki? oba biki by'okola ebisinga okukumalira ebiseera mu lunaku kobeere ng'ofunamu ssente oba nedda?	**((Record answer(s) as given):** ________________________________ ________________________ **(Code up to two responses, code first mentioned occupation first)**<br>Agriculture for home use/barter ….. 01<br>Agriculture for selling…... 02<br>Housework in your own home ….. 03<br>Housekeeper (for relative or employer)….. 04<br>Home brewing…... 05<br>Government/clerical/teaching ..06<br>Fishing….. 07<br>Student ….. 08<br>Military/police….. 09	|__|__|	**OCCUPAT1**<br>**OCCUPAT2**<br>OCCUP1

			Shopkeeper….. 10<br>Trading/vending…... 11<br>Bar worker or owner ….. . 12<br>Trucker …... 13<br>Unemployed (**PROBE _ NO AGRIC OR HOUSE WORK?**)…... 14<br>Other (specify)_________________. 15 No additional response (use in 2nd field if one occupation is cited………. 88<br>Medical worker (non-government) ..16<br>Casual laborer……………. 17<br>Waitress/Waiter/restaurant owner……….... 18<br>Hair dresser/Salon owner……………19<br>Construction (brick maker, porter, roofing, builder, painter) ……. 20<br>Mechanic (automobiles, bicycles, electronics)…..21<br>BodaBoda……………………22<br>Client/sex worker…………….23<br>Sports betting, Gambling machine,Ludo……………….24<br>NA………………………….98<br>Baking……………….….…25<br>Mining………….…….……26<br>Textiles……………….……27<br>Carpentry…………………28	|__|__|	OCCUP2

#		
	**I WOULD LIKE TO ASK YOU ABOUT WAYS IN WHICH PEOPLE CAN PLAN THEIR FAMILIES.**	**Nandyagadde okukubuuzaayo ebibuuzo bitono ebikwata ku by'okuzaala. Ebibuuzo**

	**THE QUESTION WILL HELP US TO KNOW HOW PEOPLE PLAN THEIR FAMILIES IN RAKAI AND NEIGHBOURING COMMUNITIES.**		**bino bijja kutuyamba okumanya embeera y'ebyokuzaala bweri mu Rakai ne bitundu ebigiriranye.**		
Q.9	Do you have any living children?	Olinayo omwana yenna gw'ozaala akyali omulamu?	Yes 1<br>No 2----> Q.11	|__|	LIVECHILD
Q.10	How many living children do you have?	Olina abaana bameka bozaala abakyali abalamu?	|__|__|		NUMCHILD
	**I WOULD LIKE TO ASK YOU SOME QUESTIONS ABOUT FAMILY PLANNING**		**Nandyagadde okukubuuzaayo ebibuuzo ebikwata ku ngeri abantu gye bayinza okwegemamu okuzaala oba okwegema okufuna embuto amangu.**		
Q.11	Are you/your partner currently using any family planning method?	Gwe oba omuntu yenna gwewegatta naye mulina engeri yonna ey'okwegema okuzaala gy'emukozesa kati?	Yes 1<br>No 2	|__|	FPUSING
Q.12	If yes what method are you currently using? (Un prompted) For those using calendar method, code a backup method		If yes Ngeri ki gyokozesa / zemukozesa kati okwegema okuzaala/okwegema okufuna embuto amangu ? For those using calendar method, code a backup method **(unprompted).**		
	**Current use**	Yes	No	NA	
	Pills	1	**2**	8	FPUSING1
	Condom	1	**2**	8	FPUSING2
	Depo Prov/injection	1	**2**	8	FPUSING4
	Sayana Press/self FP injection	1	**2**	8	FPUSNG17
	IUD/coil	1	**2**	8	FPUSING7
	Implant/ Norplant	1	**2**	8	FPUSNG12
	BTL/VASECTOMY	1	**2**	8	FPUSNG11
	Spermicides	1	**2**	8	FPUSING3
	Abstinence	1	**2**	8	FPUSING5
	Calendar	1	**2**	8	FPUSING6
	Lactation	1	**2**	8	FPUSING9
	Herbs	1	**2**	8	FPUSNG10
	Other method	1	**2**	8	FPUSING8
	Specify				OTHFP

#	

Q.13	Have you ever been married or entered a consensual union? **(If present union is the ONLY marriage ever, code Yes)**	Wali owasizzaako (obufumbo obw'engeri yonna nebwoba nga tewali/toli mugatte)?	Yes 1<br>No 2------>**Q.**17<br>DK 7----->**Q.17**	|__|	EVERMARR
Q.14	How old were you the first time you got married?	Walina emyaka emeka lwewasokera ddala okuwasa?	|__|__|		AG1STMAR
Q.15	Are you currently married (whether traditional, civil or religious, or in a consensual union)?	Kakati oli mufumbo (obufumbo obw'engeri yonna ne bwoba nga toli mugatte)?	Yes 1<br>No 2--------->Q.17	|__|	CURRMARR
Q.16	How many wives do you have? [**Record actual # wherever possible, otherwis [code 92= a few (01-02), 93=a lot/many(03+), no response= 99**	Olina abakyala bameka?	|__|__|		POLYMAR
Q.17	Since the last RCCS visit, have you had a change in sexual relationship	Okuva lwetwasembayo okukukyalira mu nkyala zaffe ezabuli mwaka, obufumbo bwo oba enkolagana yo ne muganzi wo/baganzi bo ekyuseeko?	Yes 1<br>No 2------->**Q.19**<br>DK 7----->**Q.19**	|__|	CHANGESR
Q.18	**If yes**, how has it changed? **(PROBE and code below)**		**If yes**, Ekyuse etya? **PROBE and code below under "since last seen")**		
			Yes	No	
	Engaged	Yayanjulwa	1	2	STATVST1
	Married	Yawasa	1	2	STATVST2
	Ended a non-marital	Baakyawagana n'omuganzi	1	2	STATVST3
	Acquired non- marital	Yafuna omuganzi	1	2	STATVST4
	Divorced/ separated marital	Yanoba/baayawukana	1	2	STATVST5
	Widowed	Yafiirwa omukyala	1	2	STATVST6
	Death of a non- marital partner	Yafiirwa omuganzi	1	2	STATVST7
	Divorced/separated with marital then got back together	Yanoba/bayawukana ne badingana	1	2	STATVST9
	Separated with non-marital then got back Together	Bakyawagana n'omuganzi ne badingana	1	2	STATVST10
	Other		1	2	OSTATVST
	Specify				OTHSTATVST
Q.19	Have you had sexual intercourse with any person in the last 12 months?	Mu myezi ekkumi n'ebiri egiyise wegasseeko n'omuntu yenna?	Yes 1<br>No 2 ----->Q.22<br>NR 9	|__|	SEXYEAR

**For questions 20-22 if more than 69 partners code 93 and write the actual number beside the box or else code actual number**				
Q.20	How many different sexual partners have you had in the last 12 months, including married or consensual partners and anyone already mentioned? **[valid codes: Record actual # wherever possible, otherwise code 92= a few (01-02), 93=3-5, 94=6-10, 95= 11-20, 96=21-30, 98= 31-40, 99=41 and above**	Mu myezi ekkumi n'ebiri egiyise, wegasse n'abantu bameka ng'obaliddemu mukyalawo/bakyalabo, muganziwo/baganzibo n'abantu abalala bonna betwogeddeko?	|__|__|	SEXP1YR

#				
Q.21	How many partners in the last twelve months were from outside this community? **[valid codes: Record actual # wherever possible, otherwise code 92= a few (01-02), 93=3-5, 94=6-10, 95= 11-20, 96=21-30, 98= 31-40, 99=41 and above**	Bameka kw'abo bewegasse nabo mu myezi ekkumi n'ebiri egiyise abataali ba mukitundu kino?	|__|__|	SEXP1OUT
Q.22	How many different sexual partners have you had in your lifetime including married or consensual partners? **[valid codes: Record actual # wherever possible, otherwise code 92= a few (01-02), 93=3-5, 94=6-10, 95= 11-20, 96=21-30, 98= 31-40, 99=41 and above**	Mu bulamu bwo wakegatta n'abantu bameka ng'obaliddemu mukyalawo, muganziwo/ baganzibo n'abantu abalala bonna betwogeddeko?	|__|__|	SEXPEVER
**FOR THOSE 15-24 years please ask Q.23 else skip to Q.24**				
Q.23	How old were you when you first developed pubic hair? (Record completed years, Code DK=97 NR=99 no pubic hair =88)	Walina emyaka emeka wewamerera ebyoya mu bitundu byo eby'ekyama? (Record completed years, Code DK=97 NR=99 no pubic hair =88)	|__|__|	PUBYEARS

IF NEVER MARRIED OR IN A RELATIONSHIP AND NO SEXUAL PARTNER IN THE 12 months (i.e. Q.9-22 ARE NO/00) THEN ASK Q.24 ELSE SKIP TO Q.25					
Q.24	Have you ever had a sexual relationship?	Wali wegasseeko n'omuntu yenna?	Yes 1<br>No 2------>**Q.78**	|__|	EVERSEX
**FIRST SEXUAL EXPERIENCE: MEN**					
Q.25	How old were you the first time you had sexual intercourse? **Record completed years, DK=97, NR=99]**	Walina emyaka emeka lwewasookera ddala okwegatta n'omuntu yenna?	|__|__|		AG1STSX

COMPUTER ID	**PLACE LABNO HERE**	CURRENT ID ____/_____/______/______

BEGIN REPETITIVE SEXUAL PARTNER BLOCKS (FOUR TOTAL)	
**(The following blocks of questions should be asked for each current and past relationship <u>in the last 12 months up to 4 partners.</u> Begin by asking about the most recent partner. If the most recent relationship was more than a year ago, but after the date when last seen still ask about this most recent partner). If no sex since last seen and not had sex in the last 12 months, then skip all repetitive sexual blocks. "Now I would like to ask you about your most recent sex partners. Please remember that all of your answers are confidential. Your answers are very important to our research to help us understand health problems in Rakai and neigbouring districts".**	**(The following blocks of questions should be asked for each current and past relationship <u>in the last 12 months up to 4 partners.</u> Begin by asking about the most recent partner. If the most recent relationship was more than a year ago, still ask about this most recent partner).**<br><br>**"Nandyagadde okukubuuzaayo ku bibuuzo bitono ebikwata ku muntu gwewakasembayo okwegatta naye. Nkukakasa nti byonna by'onombulira bikuumibwa nga bya kyama. By'ononziramu bitwalibwa nga bya mugaso nnyo mu kunoonyereza kwaffe, kitusobozese okutegeera obulungi ebikwata ku bulamu bw'abantu ba Rakai ne districts ezigiriranye."**

Q.26a	Remembering the most recent time you had sex, what was your relationship to that partner at that time?	Bw'ojjukira omulundi gwewasembayo okwegata. Omuntu oyo gw'osembyeyo/gwewasembayo okwegatta naye wali omuyita otya	|__|__|	RLTN1
Current wife (at the time) …………………………………. 01<br>Current consensual partner (at the time) …………………….. 02<br>Former wife/consensual partner……………………………… 03<br>Girlfriend…………………………………………………. 04<br>Occasional or casual friend……………………………………05<br>Visitor (incl. wedding/funeral) ………………………………06<br>Stranger……………………………………………………. 07<br>Workmate…………………………………………………. 08<br>Boss/work supervisor…………………………………………09				

Employee……………………………………………………. 10<br>Fellow student……………………………………………….. 11<br>Sugar Mummy……………………………………………12<br>Relative other than spouse**(specify)__ ___________________**13<br>Other non relative **(specify)_________________________** 14<br>Rapist (by a stranger) …………………………………… 15<br>Don't Know ……………………………………………… 97<br>Client/sex worker…………………………………………16			OTHRLTN1<br>OTHRLNR1
Q.26b	What are/were your partner's main occupations? (Record answer(s) as given):_____________________ (OCCUPAT41)<br><br>_______________________________(OCCUPAT41)<br>**(Code up to two responses, code first mentioned occupation first.)**<br>Agriculture for home use/barter…………….01<br>Agriculture for selling…………………………02<br>Housework in own home……………... 03<br>Housekeeper (for relative or employer)…….04<br>Home brewing …………………………….. 05<br>Government/clerical/teaching …………………….. 06<br>Fishing…………………………………….. 07<br>Student………………………………………08<br>Military/police ……………………………...09<br>Shopkeeper ……………………………….. 10<br>Trading/vending …………………………. 11<br>Bar worker or Owner………………………...12<br>Trucker…………………………………... 13<br>Unemployed (PROBE _ NO AGRIC OR HOUSE WORK?).. 14<br>Other _________________……………… 15<br>Medical worker (non-government)…… ….. 16<br>Casual laborer …………………………... 17<br>Waiter/restaurant owner……………………18<br>Hair dresser/Salon owner…………………...19<br>Construction (brick maker, porter builder, painter, roofing).. 20<br>Mechanic (automobiles, bicycles, electronics)…….. 21<br>Boda Boda…………………………….. 22<br>No additional response (use in 2nd field if one occupation is cited …………………………88<br>Do Not Know …………………... 97<br>Sex worker…………………………………... 23<br>Sports betting, Gambling machine, Ludo….. 24<br>NA …………………………………………….98<br>Baking…………………………………………….. 25<br>Mining……………………………………………. 26<br>Textiles…………………………………………. 27	|__|__|{OCCUP11}<br>|__|__|{OCCUP12}<br><br>Omuntu ono yali/ akola mirimu ki? oba biki by'akola/ byeyali akola/ebyali ebisinga okumumalira ebiseera mu lunaku kabeere ng’afunamu/yali afunamu ssente oba nedda?<br><br>{OCCUPS1}	P1CURR-ID

	Carpentry……………………………………… 28				

Insert spouse's household current ID if spouse is in the study area and relationship still on going . If the spouse is not in study area, code 888/888/8888/888

Spouse's Current ID**.** |__|__|__|__|/|__|__|__|__|/|__|__|__|__|__|/|__|__|__|

Q.27	How long ago did you first have sex with this person?	Wayiseewo bbanga ki okuva lwe wasookera ddala okwegatta n'omuntu ono?	Less than 1 day code 00 days,<br>Less than 1 week code days<br>Less than one month code weeks<br>Less than one year code months<br>If 1 year or more code completed Years<br>(If DK Code 97 in days and 98 in other boxes; 99 for NR)	|__|__| |__|__| |__|__| |__|__|	DAYS1<br>WEEKS1<br>MONTHS1<br>YEARS1
Q.28	How long ago, did you last have sex with this person?	Wasemba ddi okwegatta naye?	Less than 1 day code 00 days<br>Less than a week code days<br>Less than a month code weeks<br>Less than one year code months<br>If one year or more code completed years<br>(if DK code 97 in days and 98 in other boxes; 99 for NR)	|__|__| |__|__| |__|__| |__|__|	RLDYSLT1<br>RLWKSLT1<br>RLMOSLT1<br>RLYYSLT1
Q.29	Are you still in a sexual relationship with her?	Okyegatta n'omuntu oyo?	Yes 1<br>No 2<br>DK 7	|__|	RLTONGO1
Q.30	Does (was) she live (living) in this household ?	Musula (Mwali musula) wamu?	Yes 1……………Q.32<br>No 2<br>DK 7	|__|	RLTNH1
Q.31	Does (was) she regularly live (living) in this community?	Abeera (Yali abeera) mu kitundu kino ebiseera ebisinga?	Yes 1<br>No 2<br>DK 7	|__|	RLTNCM1

<table>
<tr><td>Q.32</td><td>Is/was she older, younger, or about the same age?</td><td>Omuntu oyo (yali) muto kuggwe, nga mwenkana oba nga akusinga obukulu?</td><td>Older 1
Younger 2
Same age
3……….>Q.34
Do not know 7 ..Q.34</td><td>|__|</td><td>RLTNAGE1</td></tr>
<tr><td>Q.33</td><td>About how many years [older/younger]? (Record actual # or 97=don't know)</td><td>Akusinga (Yali akusinga) oba nga omusinga emyaka emeka? Record actual # or 97=don't know)</td><td></td><td>|__|__|</td><td>RLTNYRS1</td></tr>
<tr><td>Q.34</td><td>Have you and this partner ever used a condom?</td><td>Ggwe n'omuntu oyo, mwali mukozesezza ku bupiira (condoms)?</td><td>Yes 1
No 2……>Q.36a
NR 9……Q.36 a</td><td>|__|</td><td>CNDEVER1</td></tr>
<tr><td>Q.35</td><td>During the most recent/ last 12 months you were having sexual relationship with this partner, how often did you use condoms?</td><td>Mu myezi ekkumi n'ebiri egisembyeyo /egyasembayo nga wegatta n'omuntu oyo, obupiira (mwali) mubukozesa buli kiseera, luusi na luusi oba temubukozesezako \temwabukozesako nakatono?</td><td>Never 1
Sometimes/inconsistent 2
Always 3
DK 7</td><td>|__|</td><td>RNYRCON1</td></tr>
<tr><td></td><td></td><td></td><td></td><td></td><td></td></tr>
<tr><td colspan="3"></td><td colspan="3"></td></tr>
<tr><td></td><td></td><td></td><td></td><td></td><td></td></tr>
<tr><td colspan="3">Preamble: Sometimes people enter into relationships because they need help paying for things they need, even if that is not directly exchanged for sex.</td><td colspan="3">Ebiseera ebimu abantu basalawo okwegatta mu mukwano olw’okuba nti beetaga obuyambi okusobola okugula ebintu byebaba beetaga, ne bwekiba nti tekitwaliddwa buterevu nga okwetunda oba okwegatta ofune ssente.</td></tr>
<tr><td></td><td></td><td></td><td></td><td></td><td></td></tr>
<tr><td>Q.36b</td><td>Did you enter into a sexual relationship with this partner mainly in order to get things you need, money, gifts, or other things that are important to you</td><td>Wasaalawo okwegatta n’omuntu ono okusobola okufuna ebintu bye wali wetaga,ekirabo, sente oba ebintu eby’omugaso gyoli</td><td>Yes 1
No 2</td><td>|__|</td><td>SEXNEEDS1</td></tr>
</table>

SECOND BLOCK					
**"Can you please tell me about the sexual partner just prior to the one we just discussed?" (In the last twelve months)**			**"Nandyagadde okukubuuzayo ebibuuzo bitono ebikwata ku muntu omulala gwewegatta naye mumyezi ekkumi n'ebiri egiyise eyaddibwako oyo gwetwogeddeko. (In the last twelve months)**		
Q.38	Have you had any other sexual partners in the past 12 months other than the one we just discussed?	Mu myezi ekkumi n'ebiri egiyise, wali wegasse n'or omulala yenna eyaddibwa gwetwogeddeko?	**Yes 1**<br>**No 2………Q.77**	|__|	MORE2
Q.39a	Remembering the most recent time you had sex, what was your relationship to that partner at that time?	Omuntu oyo gw'osembyeyo/gwewasembayo okw naye wali omuyita otya?		|__|__|	RLTN2
Current wife(at the time) ………………………………………. 01<br>Current consensual partner (at the time) …………………….. 02<br>Former wife/consensual partner……………………………… 03<br>Girlfriend…………………………………………………. 04<br>Occasional or casual friend……………………………………05<br>Visitor (incl. wedding/funeral) ……………………………….06<br>Stranger……………………………………………………. 07<br>Workmate…………………………………………………. 08<br>Boss/work supervisor…………………………………………09<br>Employee…………………………………………………. 10<br>Fellow student……………………………………………….. 11<br>Sugar Mummy ……………………………………………12<br>Relative other than spouse**(specify)**__ ___________________13<br>Other non relative **(specify)**__________________________ 14<br>Rapist (by a stranger) …………………………………… 15<br>Don't Know ………………………………………………… 97<br>Client/sex worker…………………………………………….16					OTHRLTN2<br>OTHRLNR2
Q.39b	What are/were your partner's main occupations? (Record answer(s) as given):______________________ (OCCUPAT21)			|__|__|{OCCUP21}<br>|__|__|{OCCUP22}<br>Omuntu ono yali/ akola mirimu	

	________________________________(OCCUPAT22)<br>**(Code up to two responses, code first mentioned occupation first.)**<br>Agriculture for home use/barter…………….01<br>Agriculture for selling…………………………02<br>Housework in own home…………….. 03<br>Housekeeper (for relative or employer)…….04<br>Home brewing …………………………….. 05<br>Government/clerical/teaching …………………….. 06<br>Fishing…………………………………….. 07<br>Student……………………………………08<br>Military/police ……………………………...09<br>Shopkeeper ……………………………... 10<br>Trading/vending ………………………… 11<br>Bar worker or Owner……………………...12<br>Trucker………………………………... 13<br>Unemployed (PROBE _ NO AGRIC OR HOUSE WORK?).. 14<br>Other _____________________………. 15<br>Medical worker (non-government)…… ….. 16<br>Casual laborer …………………………... 17<br>Waiter/restaurant owner…………………….18<br>Hair dresser/Salon owner…………………...19<br>Construction (brick maker, porter builder, painter, roofing).. 20<br>Mechanic (automobiles, bicycles, electronics)…….21<br>Boda Boda……………………………….. 22<br>No additional response (use in 2nd field if one occupation is cited …………………………………………… 88<br>Do Not Know ……………………………... 97<br>Sex worker…………………………………... 23<br>Sports betting, Gambling machine, Ludo….. 24<br>NA …………………………………………………..98<br>Baking…………………………………………….. 25<br>Mining…………………………………………….. 26<br>Textiles……………………………………………. 27<br>Carpentry………………………………………… 28	ki? oba biki by'akola/ byeyali akola/ebyali ebisinga okumumalira ebiseera mu lunaku kabeere ng'afunamu/yali afunamu ssente oba nedda?<br><br>{OCCUPS2}	

Insert spouse's household current ID if spouse is in the study area and relationship still on going . If the spouse is not in study area, code 888/888/8888/888

Spouse's Current ID. |__|__|__|/|__|__|__|/|__|__|__|__|/|__|__|__|

Q.40	How long ago did you first have sex with this person?	Wayiseewo bbanga ki okuva lwe wasookera ddala okwegatta n'omuntu ono?	Less than 1 day code 00 days,<br>Less than 1 week code days<br>Less than a month code weeks<br>Less than one year code months<br>If 1 year or more code completed Years<br>(If DK Code 97 in days and 98 in other boxes; 99 for NR)	|__|__| |__|__| |__|__| |__|__|	DAYS2<br>WEEKS2<br>MONTHS2<br>YEARS2
Q.41	How long ago, did you last have sex with this person?	Wasemba ddi okwegatta naye?	Less than 1 day code 00 days<br>Less than a week code days<br>Less than a one month code weeks<br>Less than one year code months<br>(if DK code 97 in days and 98 in other boxes; 99 for NR)	|__|__| |__|__| |__|__|	RLDYSLT2<br>RLWKSLT2<br>RLMOSLT2
Q.42	Are you still in a sexual relationship with her?	Okyegatta n'omuntu oyo?	Yes 1<br>No 2<br>DK 7	|__|	RLTONGO2
Q.43	Does (was) she live (living) in this household ?	Musula (Mwali musula) wamu?	Yes 1………Q.45<br>No 2<br>DK 7	|__|	RLTNH2
Q.44	Does (was) she regularly live (living) in this community?	Abeera (Yali abeera) mu kitundu kino ebiseera ebisinga?	Yes 1<br>No 2<br>DK 7	|__|	RLTNCM2
Q.45	Is/was she older, younger, or about the same age?	Omuntu oyo (yali) muto kuggwe, nga mwenkana oba nga akusinga obukulu?	Older 1<br>Younger 2<br>Same age 3….>Q.47<br>Do not know 7..Q.47	|__|	RLTNAGE2
Q.46	About how many years [older/younger]? **(Record actual # or 97=don't know)**	Akusinga (Yali akusinga) oba nga omusinga emyaka emeka? **Record actual # or 97=don't know)**	|__|__|		RLTNYRS2
Q.47	Have you and this partner ever used a condom?	Ggwe n'omuntu oyo, mwali mukozesezza	Yes 1<br>No 2……>Q.49a<br>NR 9……Q.49a	|__|	CNDEVER2

		ku bupiira (condoms)?			
Q.48	During the most recent/ last 12 months you were having sexual relationship with this partner, how often did you use condoms?	Mu myezi ekkumi n'ebiri egisembyeyo /egyasembayo nga wegatta n'omuntu oyo, obupiira (mwali) mubukozesa buli kiseera, luusi na luusi oba temubukozesezako \temwabukozesako nakatono?	Never 1<br>Sometimes/inconsistent 2<br>Always 3<br>DK 7	|__|	RNYRCON2
Preamble: Sometimes people enter into relationships because they need help paying for things they need, even if that is not directly exchanged for sex.			**Ebiseera ebimu abantu basalawo okwegatta mu mukwano olw'okuba nti beetaga obuyambi okusobola okugula ebintu byebaba beetaga, ne bwekiba nti tekitwaliddwa buterevu nga okwetunda oba okwegatta ofune ssente.**		
Q.49b	*Did you enter into a sexual relationship with this partner mainly in order to get things you need, money, gifts, or other things that are important to you*	*Wasaalawo okwegatta n'omuntu ono okusobola okufuna ebintu bye wali wetaga,ekirabo, sente oba ebintu eby'omugaso gyoli*	Yes 1<br>No 2	|__|	SEXNEEDS2

**THIRD BLOCK**					
**"Can you please tell me about the sexual partner just prior to the one we just discussed?" (In the last twelve months)**			**"Nandyagadde okukubuuzayo ebibuuzo bitono ebikwata ku muntu omulala gwewegatta naye mumyezi ekkumi n'ebiri egiyise eyaddibwako oyo gwetwogeddeko. (In the last twelve months)**		
Q.50	Have you had any other sexual partners in the past 12 months other than the one we just discussed?	Mu myezi ekkumi n'ebiri egiyise wegasse n'omuntu omulala yenna eyaddibwako oyo gwetwoge	**Yes 1**<br>**No 2…Q.77**	|__|	MORE3

Q.51 a	Remembering the most recent time you had sex, what was your relationship to that partner at that time?	Omuntu oyo gw'osembyeyo/gwewasembayo okw naye wali omuyita otya?	|__|__|	RLTN3

Current wife(at the time) ……………………………………. 01<br>Current consensual partner (at the time) …………………….. 02<br>Former wife/consensual partner……………………………. 03<br>Girlfriend…………………………………………………. 04<br>Occasional or casual friend…………………………………05<br>Visitor (incl. wedding/funeral) ……………………………….06<br>Stranger……………………………………………………. 07<br>Workmate…………………………………………………. 08<br>Boss/work supervisor………………………………………09<br>Employee…………………………………………………. 10<br>Fellow student……………………………………………….. 11<br>Sugar Mummy ……………………………………………12<br>Relative other than spouse**(specify)__ ___________________**13<br>Other non relative **(specify)__________________________** 14<br>Rapist (by a stranger) …………………………………… 15<br>Don't Know ……………………………………………… 97<br>Client/sex worker…………………………………………….16	OTHRLTN3<br>OTHRLNR3

Q.52b	What are/were your partner's main occupations? (Record answer(s) as given):______________________ (OCCUPAT31)<br>______________________________(OCCUPAT32)<br>**(Code up to two responses, code first mentioned occupation first.)**<br>Agriculture for home use/barter…………….01<br>Agriculture for selling…………………02<br>Housework in own home…………… 03<br>Housekeeper (for relative or employer)…….04<br>Home brewing …………………….. 05<br>Government/clerical/teaching …………….. 06<br>Fishing…………………………... 07<br>Student……………………………08<br>Military/police ……………………...09<br>Shopkeeper ………………………... 10<br>Trading/vending …………………. 11<br>Bar worker or Owner……………...12<br>Trucker…………………………...13<br>Unemployed (PROBE _ NO AGRIC OR HOUSE WORK?).. 14<br>Other _____________………………. 15<br>Medical worker (non-government)……16<br>Casual laborer ……………………... 17	|__|__|{OCCUP31}<br>|__|__|{OCCUP32}<br><br>Omuntu ono yali/ akola mirimu ki? oba biki'by'akola/ byeyali akola ebisinga okumumalira ebiseera mu lunaku kabeere ng'afunamu/yali afunamu ssente oba nedda?	

	Waiter/restaurant owner…………….18<br>Hair dresser/Salon owner…………...19<br>Construction (brick maker, porter builder, painter, roofing).. 20<br>Mechanic (automobiles, bicycles, electronics)…….. 21<br>Boda Boda………………………….. 22<br>No additional response (use in 2nd field if one occupation is cited ………………………….88<br>Do Not Know …………………….. 97<br>Sex worker…………………………... 23<br>Sports betting, Gambling machine, Ludo…24<br>NA ………………………………………..98<br>Baking……………………………….. 25<br>Mining……………………………….. 26<br>Textiles………………………………. 27<br>Carpentry…………………………… 28	{OCCUPS3}	

Insert spouse's household current ID if spouse is in the study area and relationship still on going . If the spouse is not in study area, code 888/888/8888/888

Spouse's Current ID. |__|__|__|/|__|__|__|/|__|__|__|__|/|__|__|__|

Q.52	How long ago did you first have sex with this person?	Wayiseewo bbanga ki okuva lwe wasookera ddala okwegatta n'omuntu ono?	Less than 1 day code 00 days,<br>Less than 1 week code days<br>Less than one month code weeks<br>Less than one year code months<br>If 1 year or more code completed Years<br>(If DK Code 97 in days and 98 in other boxes; 99 for NR)	|__|__| |__|__| |__|__| |__|__|	DAYS3<br>WEEKS3<br>MONTHS3<br>YEARS3
Q.53	How long ago, did you last have sex with this person?	Wasemba ddi okwegatta naye?	Less than 1 day code 00 days<br>Less than a week code days<br>Less than a month code weeks<br>Less than one year code months	|__|__| |__|__| |__|__|	RLDYSLT3<br>RLWKSLT3<br>RLMOSLT3

			(if DK code 97 in days and 98 in other boxes; 99 for NR)		
Q.54	Are you still in a sexual relationship with her?	Okyegatta n'omuntu oyo?	Yes 1<br>No 2<br>DK 7	|__|	RLTONGO3
Q.55	Does (was) she live (living) in this household ?	Musula (Mwali musula) wamu?	Yes 1………Q.58<br>No 2<br>DK 7	|__|	RLTNH23
Q.56	Does (was) she regularly live (living) in this community?	Abeera (Yali abeera) mu kitundu kino ebiseera ebisinga?	Yes 1<br>No 2<br>DK 7	|__|	RLTNCM3
Q.57	Is/was she older, younger, or about the same age?	Omuntu oyo (yali) muto kuggwe, nga mwenkana oba nga akusinga obukulu?	Older 1<br>Younger 2<br>Same age 3….>Q.60a<br>Do not know 7..Q.60a	|__|	RLTNAGE3
Q.58	About how many years [older/younger]? **(Record actual # or 97=don't know)**	Akusinga (Yali akusinga) oba nga omusinga emyaka emeka? **Record actual # or 97=don't know)**	|__|__|		RLTNYRS3
Q.59	Have you and this partner ever used a condom?	Ggwe n'omuntu oyo, mwali mukozesezza ku bupiira (condoms)?	Yes 1<br>No 2……>Q.62a<br>NR 9……Q.62a	|__|	CNDEVER3
Q.60	During the most recent/ last 12 months you were having sexual relationship with this partner, how often did you use condoms?	Mu myezi ekkumi n'ebiri egisembyeyo /egyasembayo nga wegatta n'omuntu oyo, obupiira (mwali) mubukozesa buli kiseera, luusi na luusi oba temubukozesezako \temwabukozesako nakatono?	Never 1<br>Sometimes/inconsistent 2<br>Always 3<br>DK 7	|__|	RNYRCON3
Preamble: Sometimes people enter into relationships because they need help paying for things they need, even if that is not directly exchanged for sex.			**Ebiseera ebimu abantu basalawo okwegatta mu mukwano olw'okuba nti beetaga obuyambi okusobola okugula ebintu byebaba beetaga, ne bwekiba nti tekitwaliddwa buterevu nga okwetunda oba okwegatta ofune ssente.**		

Q.62b	*Did you enter into a sexual relationship with this partner mainly in order to get things you need, money, gifts, or other things that are important to you*	*Wasaalawo okwegatta n'omuntu ono okusobola okufuna ebintu bye wali wetaga,ekirabo,sente oba ebintu eby'omugaso gyoli*	Yes 1<br>No 2	|__|	SEXNEEDS3

FOURTH BLOCK

**"Can you please tell me about the sexual partner just prior to the one we just discussed?" (In the last twelve month)**	**"Nandyagadde okukubuuzayo ebibuuzo bitono ebikwata ku muntu omulala gwewegatta naye mumyezi ekkumi n'ebiri egiyise eyaddibwako oyo gwetwogeddeko. (In the last twelve months)**

Q.61	Have you had any other sexual partners in the past 12 months other than the one we just discussed?	Mu myezi ekkumi n'ebiri egiyise, wali wegasse n'or omulala yenna eyaddibwa gwetwogeddeko?	**Yes 1**<br>**No 2………Q.77**	|__|	MORE4
Q.62 a	Remembering the most recent time you had sex, what was your relationship to that partner at that time?	Omuntu oyo gw'osembyeyo/gwewasembayo okwegatta naye wali omuyita otya?		|__|__|	RLTN4

Current wife(at the time) …………………………………… 01<br>Current consensual partner (at the time) …………………….. 02<br>Former wife/consensual partner……………………………… 03<br>Girlfriend…………………………………………………. 04<br>Occasional or casual friend…………………………………05<br>Visitor (incl. wedding/funeral) ………………………………06<br>Stranger…………………………………………………… 07<br>Workmate…………………………………………………. 08<br>Boss/work supervisor………………………………………09<br>Employee…………………………………………………. 10<br>Fellow student…………………………………………….. 11<br>Sugar Mummy ………………………………………………12<br>Relative other than spouse**(specify)**__ ___________________13<br>Other non relative **(specify)**___________________________ 14<br>Rapist (by a stranger) …………………………………… 15<br>Don't Know ……………………………………………… 97<br>Client/sex worker……………………………………………16	OTHRLTN4<br>OTHRLNR4

Q.65 b	What are/were your partner's main occupations?	|__|__|{OCCUP41}<br>|__|__|{OCCUP42}

	(Record answer(s) as given):______________________ (OCCUPAT41)<br><br>________________________________(OCCUPAT42)<br>**(Code up to two responses, code first mentioned occupation first.)**<br>Agriculture for home use/barter…………….01<br>Agriculture for selling………………………02<br>Housework in own home……………... 03<br>Housekeeper (for relative or employer)…….04<br>Home brewing …………………………….. 05<br>Government/clerical/teaching …………………….. 06<br>Fishing……………………………………... 07<br>Student……………………………………08<br>Military/police …………………………...09<br>Shopkeeper ……………………………….. 10<br>Trading/vending …………………………. 11<br>Bar worker or Owner………………………...12<br>Trucker……………………………...13<br>Unemployed (PROBE _ NO AGRIC OR HOUSE WORK?).. 14<br>Other __________________………………. 15<br>Medical worker (non-government)…… ….. 16<br>Casual laborer …………………………….. 17<br>Waiter/restaurant owner……………………18<br>Hair dresser/Salon owner…………………...19<br>Construction (brick maker, porter builder, painter, roofing)…………….. 20<br>Mechanic (automobiles, bicycles, electronics)……..21<br>Boda Boda………………………………….. 22<br>No additional response (use in 2nd field if one occupation is cited ……………………………….88<br>Do Not Know …………………………... 97<br>Sex worker…………………………………...<br>23<br>Sports betting, Gambling machine,Ludo……….. 24<br>NA ………………………………………..98<br>Baking…………………………………………….. 25<br>Mining…………………………………………….. 26<br>Textiles…………………………………. 27<br>Carpentry…………………………………………… 28	Omuntu ono yali/ akola mirimu ki? oba biki by'akola/ byeyali akola/ebyali ebisinga okumumalira ebiseera mu lunaku kabeere ng’afunamu/yali afunamu ssente oba nedda?<br><br>{OCCUPS4}		
**Insert spouse’s household current ID if spouse is in the study area and**				

**relationship still on going . If the spouse is not in study area, code 888/888/8888/888**<br>Spouse's Current ID. |__|__|__|/|__|__|__|/|__|__|__|__|/|__|__|__|					PCURR_ID4
Q.63	How long ago did you first have sex with this person?	Wayiseewo bbanga ki okuva lwe wasookera ddala okwegatta n'omuntu ono?	Less than 1 day code 00 days,<br>Less than 1 week code days<br>Less than one month code weeks<br>Less than one year code months<br>If 1 year or more code completed Years<br>(If DK Code 97 in days and 98 in other boxes; 99 for NR)	|__|__| |__|__| |__|__| |__|__|	DAYS4<br>WEEKS4<br>MONTHS4<br>YEARS4
Q.64	How long ago, did you last have sex with this person?	Wasemba ddi okwegatta naye?	Less than 1 day code 00 days<br>Less than a week code days<br>Less than a month code weeks<br>Less than one year code months<br>(if DK code 97 in days and 98 in other boxes; 99 for NR)	|__|__| |__|__| |__|__|	RLDYSLT4<br>RLWKSLT4<br>RLMOSLT4
Q.65	Are you still in a sexual relationship with her?	Okyegatta n'omuntu oyo?	Yes 1<br>No 2<br>DK 7	|__|	RLTONGO4
Q.66	Does (was) she live (living) in this household?	Musula (Mwali musula) wamu?	Yes 1………Q.71<br>No 2<br>DK 7	|__|	RLTNH4
Q.67	Does (was) she regularly live (living) in this community?	Abeera (Yali abeera) mu kitundu kino ebiseera ebisinga?	Yes 1<br>No 2<br>DK 7	|__|	RLTNCM4

Q.68	Is/was she older, younger, or about the same age?	Omuntu oyo (yali) muto kuggwe, nga mwenkana oba nga akusinga obukulu?	Older 1<br>Younger 2<br>Same age 3….>Q.73<br>Do not know 7 ..Q.73	|__|	RLTNAGE4
Q.69	About how many years [older/younger]? **(Record actual # or 97=don't know)**	Akusinga (Yali akusinga) oba nga omusinga emyaka emeka? **Record actual # or 97=don't know)**	|__|__|		RLTNYRS4
Q.70	Have you and this partner ever used a condom?	Ggwe n'omuntu oyo, mwali mukozesezza ku bupiira (condoms)?	Yes 1<br>No 2……>Q.75a<br>NR 9……Q.75a	|__|	CNDEVER4
Q.71	During the most recent/ last 12 months you were having sexual relationship with this partner, how often did you use condoms?	Mu myezi ekkumi n'ebiri egisembyeyo /egyasembayo nga wegatta n'omuntu oyo, obupiira (mwali) mubukozesa buli kiseera, luusi na luusi oba temubukozesezako \temwabukozesako nakatono?	Never 1<br>Sometimes/inconsistent 2<br>Always 3<br>DK 7	|__|	RNYRCON4
Preamble: Sometimes people enter into relationships because they need help paying for things they need, even if that is not directly exchanged for sex.			**Ebiseera ebimu abantu basalawo okwegatta mu mukwano olw'okuba nti beetaga obuyambi okusobola okugula ebintu byebaba beetaga, ne bwekiba nti tekitwaliddwa buterevu nga okwetunda oba okwegatta ofune ssente.**		
Q.75b	*Did you enter into a sexual relationship with this partner mainly in order to get things you need, money, gifts, or other things that are important to you*	*Wasaalawo okwegatta n'omuntu ono okusobola okufuna ebintu bye wali wetaga,ekirabo, sente oba ebintu eby'omugaso gyoli*	Yes 1<br>No 2	|__|	SEXNEEDS4

END OF REPETITIVE SEXUAL BLOCK
IF NOT MARRIED AND NO SEX IN LAST 12 MONTHS SKIP TO Q78

**Disagreements on some issues can occur between men and women, which sometimes result into violence. i would like to ask you some questions on violence.**	**Mu nkolagana ez'omukwano wakati w'abaami n'abakyala obutakkaanya ku bintu ebimu butera okubaawo, emirundi egimu buvaamu obusambattuko. nandyagadde okukubuuzaayo ebibuuzo ku busambattuko.**		
Q.77 In the past 12 months did you do any of the following to any of your partners: **PROMPTED**	Mu myezi ekkumi n'ebiri egiyise, omuntu yena gwe wegatta naye wali omukozeeko ebintu bino wammanga? [PROMPT		
		Yes No NA	
Verbally abuse or shout at her?	Okumuvuma/okumuwogganira/ Okumuboggolera	1 2 8	PABUSEYR
Push, slap, punch, kick, drag or otherwise physically hurt her?	Okumusindika/ okumukuba oluyi /okumukuba ekikonde/okumusamba/okumusikambura/ oba okumulumya ku mubiri?	1 2 8	PSPKDYR
Forced her to have sex when she did not want to?	Okumukaka okwegatta nga tayagala.	1 2 8	OTHFSXPY
Other	Ekirala	1 2 8	POTHVYR
specify			OTHPVYRS

<table>
<tr><td colspan="3">I am now going to ask you some questions about hiv testing. Please know that whatever answers you provide will be kept strictly confidential</td><td colspan="3">Ngenda kukubuuzaayo ebibuuzo ebikwata ku kwekebeeza akawuka ka siriimu. Byonotuddamu byonna bijja kukumibwa nga byakyama.</td></tr>
<tr><td>Q.78</td><td>Have you ever received your HIV results from anywhere?</td><td>Wali ofunyeko ebyava mu musaayi gwo ebikwata ku kawuka ka siriimu okuva awantu wonna?</td><td>Yes 1
No 2------------->Q. 83a
NR 9------------->Q. 83a
NA(Never tested)8-->Q. 83a</td><td>|__|</td><td>RHIVEVER</td></tr>
<tr><td></td><td></td><td></td><td></td><td></td><td></td></tr>
<tr><td>Q.79</td><td>How long ago did you last receive your last HIV results?

Please give month and year if you can.</td><td>Wayisewo banga ki okuva lwewasembayo okufuna ebyava mu kukebeera omusaayi ebikwata ku kawuka ka siriimu?</td><td>code in days, weeks, months, years if completed years code 00 in the rest of the boxes if less than a week,month and year code 98 (If DK Code 97 in days and 98 in other boxes; 99 for NR)</td><td>|__|__|Days
|__|__|weeks
|__|__|months
|__|__|years</td><td>HIVPERIODD
HIVPERIODW
HIVPERIODM
HIVPERIODY</td></tr>
<tr><td>Q.80</td><td>What was the result of this last HIV test?</td><td>Ebyava mu musaayi gwo ebisembyeyo byali bitya?</td><td>Negative 1____Q.83a
Positive 2
Indeterminate 3___Q.83a
No Response 9
Don’t know/Don’t remember 7____Q.83a</td><td>|__|</td><td>HIVRSLT</td></tr>
</table>

Q.81	When was your first positive HIV test? Please give month and year if you can.	Ddi lwebasokeraddala okukebera nebakizuula/bakugamba nti olina akawuuka akaleeta siriimu? Please give month and year if you can.	Month<br><br>Year<br><br>DK/NR 9997	|__|__| |__|__|__|__|	FHIVTESTM<br>FHIVTESTY
Q82	When was your last negative HIV test? This would be your last negative before you tested positive. Please give month and year.	Basemba ddi okukukebera n'osangibwa nga tolina kawuka akaleeta siriimu? guno guyinza okuba nga gwe murundi gwewasembayo okwekebeza no sangibwa nga tolina kawuka ka siriimu Please give month and year.	Month<br><br>Year<br><br>DK/NR 9997, 9998 first time they tested, they were HIV positive or were born with HIV	|__| <br>|__|__|__|__|	LHIVTSTNM<br><br>LHIVTSTNY
Q83a	In the past 12 months, have you tried to get an HIV test at a health care facility but were refused?	Mu myezi ekumi nebbiri egiyise, ogenzeko ewakeberelwa okugezako okwekebeza akawuuka akaleeta sirimu mu musaayi nebagana okukukebera?	Yes 1<br>No 2<br>NR 9		THIVTSTR
Q.83b	Have you ever been contacted by partner notification services to get HIV tested?	Otukiridwako omuntu yenna nga akulaga obwetavu bwo kwekebeza akawuka ka silimu mu musaayi okugeza nga omusawo okukubira essimu oba okukutukirira mu buntu oba omwagalwa wo okukikutegezaako?	Yes 1<br>No 2------------→Q.85<br>NR 9----------→ Q.85	|__|	APN
Q.84	Did you get HIV tested because you	Wekebeza akawuka akaleeta silimu mu musaayi olwokuba	Yes 1<br>No 2<br>NR 9	|__|	APNTEST

	were contacted by partner notification services	omusawo oba omwagalwa wo yakukubira essimu oba okukutukirira mu buntu?	Already on ART 3		
Q.85	Have you ever used PreP?	Wali omizeeko ku ddagala erikendeza ku katyabaga akokukwatibwa akawuka ka siriimu PrEP?	Yes 1<br>No 2-------------Q.87<br>DK/NR 7	|__|	PREPEVER
Q.86	Are you currently using PreP?	Mukiseera kino omira eddagala erikendeza ku katyabaga kokukwatibwa akawuka ka siriimu PreP?	Yes 1<br>No 2<br>NR 7	|__|	PREPUSE

<table>
<tr><th>#</th><th colspan="5"></th></tr>
<tr><td colspan="6">FOR PERSONS WHO ARE HIV POSITIVE (I.E RESPONSE TO Qn 80 IS 2) ASK Qn 88 ELSE SKIP TO 93</td></tr>
<tr><td colspan="3">We want to ask you about use of health care services and long term medication. this information will be strictly confidential. you do not have to answer any of these questions.</td><td colspan="3">Nandyagadde okukubuzayo ebibuuzo ebikwata ku ngeri abantu gyebakozesamu obujjanjabi ne ku ddagala erimiribwa abantu okumala ebbanga egwanvu. By'ononziramu byonna bijja kukumibwa nga byakyama. Osobola okuddamu ebibuuzo ebimu ebirala n'obireka.</td></tr>
<tr><td>Q.88</td><td>Have you ever been to a clinic to receive care for HIV?</td><td>Wali obaddeko/ogenzeko mu kifo kyona ew'ajjanjabirwa okufuna edaggala erikendeeza obungi bw'akawuka ka sirimu mu musaayi (ARVS)?</td><td>Yes 1
No 2--skip to 93
DK 7__Q.93
NR 9</td><td>|__|</td><td>HIVCARE</td></tr>
<tr><td>Q.89</td><td>Have you ever been on ARVs?</td><td>Omira/wali omizeeko eddagala erikendeeza obungi bw'akawuka ka siriimu mu musaayi (ARVS)?</td><td>Yes 1
No 2__Q.93
DK 7___Q.93
NR 9</td><td>|__|</td><td>ARVMED</td></tr>
</table>

Q.90	Are you currently taking ARVs?	Eddagala lino erikendeeza obungi bw'akawuka ka siriimu mu musaayi olimira kati (ARVS)?	Yes 1<br>No 2<br>DK 7<br>NR 9	|__|	CUARVMED
Q.91a	What is/was your current source of ARVs? **{Get codes fromthe table below}**	Eddagala lino erikendeeza obungi bwa akawuka ka siriimu mu musaayi (ARVS) otera/wateranga kulifuna kuva wa?	|__|__|__| Other arv current source<br>___________________________________<br><br>Other clinic type for current medication<br>Gov't 1<br>NGO 2<br>Private 3<br>|__|		ARVSOURC2<br><br>OTHARVSOU1<br><br>ARVTYPE1

HEALTH UNITS CODES FOR RAKAI DISTRICT 2008 TO DATE

NAME OF HEALTH UNIT	CODE	OWNERSHIP
Baale Gunda	037	GOV'T
Bakka	104	GOV'T
Bethrehem	010	GOV'T
Bikira	002	NGO
Bitabago	047	PRIVATE
Bugona	099	GOV'T
Bukeeri HCIII	123	GOV'T
Bulamu	100	GOV'T
Butembe	078	GOV'T
Butiti	098	GOV'T
Buyamba Dwaniro	028	GOV'T
Buyamba NGO	050	NGO
Buyiisa	072	GOV'T
Buziranduulu	015	GOV'T

Byakabanda	076	GOV'T
Byerima	095	GOV'T
Gayaaza	005	GOV'T
Gwanda	085	GOV'T
Heal the nation	044	NGO
Kabira	012	GOV'T
Kabusota	070	GOV'T
Kabuwoko H/C	006	GOV'T
Kabuwoko NGO	007	NGO
Kacheera	031	GOV'T
Kagamba	027	GOV'T
Kakundi	033	GOV'T
Kakuuto	016	GOV'T
Kaleere	034	GOV'T
Kalisizo H/C	087	NGO
Kalisizo T.C/Hosp	001	GOV'T
Kalisizo/Kyango	014	GOV'T
Kamulegu H/C III	120	GOV’T
Kasaali	004	GOV'T
Kasankala	055	GOV'T
Kasankala NGO	059	NGO
Kasasa	023	GOV'T
Kasensero	089	GOV'T
Katatenga	049	GOV'T
Kayanja	063	GOV'T
Kayanja Prison	045	GOV'T
Kayayumbe	048	NGO
Kayonza	035	GOV'T
Kayonza Ddwaniro	103	GOV'T
Kibaale H/C II	030	GOV'T
Kibaale Home	067	NGO
Kibanda	022	GOV'T

Kibanda Clinic	068	PRIVATE
Kibuuka	011	GOV'T
Kifamba	021	GOV'T
Kijjeja	086	GOV'T
Kijonjo	097	GOV'T
Kimuli	041	GOV'T
Kirumba	101	GOV'T
Kiziba	051	GOV'T
Kitovu Hospital	118	Private
Kitovu Mobile	119	NGO
Kyabigondo	057	GOV'T
Kyakanyomozi	079	GOV'T
Kyakonda	074	GOV'T
Kyakuwa	092	GOV'T
Kyalulangira	065	GOV'T
Kyanamukaaka HC IV	121	GOV'T
Kyebe	018	GOV'T
Kyempewo	102	GOV'T
Kyotera G/C	071	PRIVATE
Kyotera M/C	080	PRIVATE
Kyotera Moslems	075	NGO
Kyotera T.C/Mitukula	024	GOV'T
Lukerere	061	GOV'T
Lwabakooba	056	GOV'T
Lwakaloolo	054	GOV'T
Lwamaggwa NGO	069	NGO
Lwamaggwwa	029	GOV'T
Lwamba	036	GOV'T
Lwanda	025	GOV'T
Lwankoni	042	GOV'T
Lwembajjo	052	GOV'T
Lwensinga	060	GOV'T

Lyantonde Hospital	122	GOV'T
Magabi	082	GOV'T
Mayanja	090	GOV'T
Mbuye	026	NGO
Micungiro	038	GOV'T
Minziiro	040	GOV'T
Mirigwe	094	GOV'T
Mukisa	064	PRIVATE
Mutukula	017	GOV'T
Muzito	081	PRIVATE
Nabigasa	009	GOV'T
Nabyajjwe	062	GOV'T
Nakasoga	084	GOV'T
Nakatoogo	008	GOV'T
Nangoma	020	GOV'T
Nazareth	088	NGO
Ndolo	039	GOV'T
Nkenge	053	GOV'T
No additional Response	888	
Not applicable	998	
Others	115	
Rakai Program Kalisizo Hub	105	NGO
Rakai hospital	032	GOV'T
Sanje Dom	046	PRIVATE
Sserulanda	013	GOV'T
St. Bernard Mannya	093	NGO
St. Joseph	083	PRIVATE
St. Martine	043	PRIVATE
St.Gyaviira	058	PRIVATE
St.Mugagga	073	PRIVATE
Taso Masaka	117	NGO
Uganda Cares Masaka	116	NGO

Q91b	**How long ago did you first start ART medications?**	Wayiseewo bbanga ki okuva lwe watandika okumira eddagala erikendeza obungi bw'akawuka ka siriimu mu musaayi?	_ ARTDAYS (0-6 acceptable responses)<br><br>_ ARTWKS (1-3 acceptable responses)<br><br>ARTMOS (1-11 acceptable responses)<br><br>ARTYRS (1 – 20 acceptable responses)<br><br>**## Code 97 for Don't know in ARTDAYS**	|__|__|{artdays}<br><br>|__|__|{artwks}<br><br>|__|__|{artmos}<br><br>|__|__|{artyrs}

2						

IF YES TO Q.89 PLEASE ASK

Q.91a. In the past year, did you change the clinic where you receive HIV care?

Mu myezi ekumi n'ebbiri egise okyusizako eddwaliro w'ofunira eddagala erikendeza obungi bw'akawuka ka siriimu mu musaayi?

- Yes
- No
- Unsure/don't remember
- Refused

Q91b. What motivated you to change the clinic where you receive HIV care? Kiki ekyakusikiriza okukyusa eddwaliro w'ofunira eddagala erikendeza obungi bw'akawuka ka siriimu mu musaayi?

[*SELECT ALL THAT APPLY*]

- I didn't want to be seen or recognized when accessing services
- Clinic was too far away
- Clinic was too crowded or busy
- I relocated to a new community
- I was mistreated by staff or providers
- I thought I could get better care somewhere else
- Clinic closed or shut down
- Other, specify: ________

Q91c. Were you issued a transfer form before you switched to the new facility? **Wafuna ebaluwa eyakukiriza okukyusa eddwaliro w'ofunira eddagala erikendeza obungi bw'akawuka ka siriimu mu musaayi okugenda ku ddwaliro eddala?**

- Yes (*formal transfer*)
- No (*self-transfer*)
    - Unsure/don't remember
    - Refused

Q91d. Did you disclose your HIV status or ARV use history when you enrolled in the new facility? **Bwewagenda okufuna eddagala erikendeza obungi bw'akawuka ka siriimu mu musaayi ewapya wabategeza nti omira eddagala erikendeza akawuka akaleeta obulwadde bwa siriimu?**

- Disclosed both HIV status and ARV use history
- Disclosed only HIV status (not ARV use history)
- Did not disclose HIV status (retested for HIV)
- Unsure/don't remember

- Disclosed both HIV status and ARV use history
- Disclosed only HIV status (not ARV use history)
- Did not disclose HIV status (retested for HIV)
- Unsure/don't remember

"People sometimes forget to take their ARVs daily. Olw'olumu abantu berabira okumira eddagala lyabwe erikendeza obungi bw'akawuka ka siriimu mu musaayi buli luna
Q.92a In the past 30 days, how many days have you missed taking any ARV pills?"Mu naku amakumi assaatu eziyise naku meka zewerabira okumira eddagala lyo erikendeza akawuka ka siriimu mu musaayi? Response should be 0-30.

Q. 92c Through which of the following service delivery models do you currently receive your ARVs?<br><br>Ku bino wammanga ngeriki gy'ofunamu eddagala lyo erikendeeza obungi bwa akawuka ka siriimu mu musaayi(ARVs)	Pick-up at a clinic or hospital after interacting with a clinician (FBIM) 01<br><br>Participate in an adherence support group where we receive our ARVs at the facility (FBG) 02<br><br>Pick-up at a clinic or hospital directly at the pharmacy *without* interacting with a clinician or nurse (FTDR) 03<br><br>Direct delivery by a neighbor, family member, facility peer, or other provider (CCLAD) 04<br><br>Pick-up at a pop-up space set up by providers or peers in the community (CDDP) 05<br><br>Pick-up at a private or retail pharmacy in the community 06<br><br>Other, specify: _____	Ndifuna okuva mu dwaliro ng'omusaawo amaze okundaba oba okunzijjanjaba (FBIM) 01<br><br>Ndifuna okuva ku ddwaliro nga maze okufuna okubuddabuddibwa okwawamu ku kumira eddagala lyange obulungi (FBG) 02<br><br>Ndifuna okuva mu dwaliro buterevu ku dirisa webagabira eddagala nga simaze kwogera namusaawo yena ajjanjaba (FTDR) 03<br><br>Abantu ab'enjawulo balindetera buterevu okugeza balirwana bange,ab'enganda zange mikwano gyange oba abagaba eddagala abalala (CCLAD) 04<br><br>Ndifuna okuva mu kitundu okiba kitekeddwawo abasaawo mu kitundu wetusobola okufunira eddagala oba ku balwadde banange 05<br><br>Ndifuna okuva webatunda eddagala mu kitundu mwembera pharmacy 06<br><br>Other, specify: _____	|___|{DSDM}}<br><br>ARTCURMEDS

#	
**International Physical Activity Questionnaire**	
Think about all the vigorous activities that you did in the last 7 days. Vigorous physical activities refer to activities that take	Lowooza ku bintu byokoze nga byeetaaga amanyi mu nnaku omusanvu eziyise. Ebintu ebikolebwa namanyi

hard physical effort and make you breathe much harder than normal, like heavy lifting, digging, vigorous exercise, fetching water, or fast bicycling. Think only about those physical activities that you did for at least 10 minutes at a time.			byebyo ebyetaaga okuteekamu amaanyi amangi nebikuleetera okussa enyo okusinga kumbeera eyabulijo. okugeza okusitula ebizitowa, okulima,okukima amazzi oba okuvuga akagaali mubwangu.Lowooza kwebyo byokka ebyakolebwa mu ddakiika kkumi(10minutes) omulundi ggumu.	
Q.93	During the last 7 days, on how many days did you do vigorous physical activities?	Mu nnaku omusanvu eziyise, ennaku mmeka z'okoze ebintu ebyetaaga amanyi amanji?	|__| days [range 0-7] per week<br>If 0, skip to Q.95	IPAQ1
Q.94	How much time did you usually spend doing vigorous physical activities on one of those days?	Watwalangabudde ki ngokola ebintu ebyetaaga amanyi amangi mu lunaku olumu kwezo?	|__|__| hours per day<br>|__|__| minutes per day<br>97 Don't know/Not sure	IPAQ2h<br>IPAQ2m
Think about all the moderate activities that you did in the last 7 days. Moderate activities refer to activities that take moderate physical effort and make you breathe somewhat harder than normal, like carrying light loads, bicycling at a regular pace, sweeping, or moderate exercise? Do not include walking. Think only about those physical activities that you did for **at least 10 minutes at a time.**			***Munnaku omusanvu eziyise, Lowooza ku bintu byokoze nga byetaaga amaanyi agekigero. Ebintu ebikolebwa namaanyi agekigero byebyo ebyetaaga okuteekamu amaanyi agekigero nebikuleetera okussa namaanyi amangiko okusinga ku mbeera eyabulijo okugeza okusutula ebintu ebiwewuka ebitali bizito, okuvuga eggali mu mpolampola, okweera. Lowooza kwebyo byokka ebyakolebwa wakiri mu ddakika kumi(10minutes) omulundi gumu.***	
Q.95	During the last 7 days, on how many days did you do moderate physical activities?	Mu nnaku musanvu eziyise, ennaku mmeka zokozemu ebintu ebyetaaga amaanyi agekigero?	|__| days<br>[range 0-7] per week<br>If 0, skip to Q.97	IPAQ3
Q.96	How much time did you usually spend doing moderate physical activities on one of those days?	Watwalanga budde ki nga okola ebintu ebyetaaga amanyi agekigero mulunaku olumu kwezo?	|__|__| hours per day<br>|__|__| minutes per day<br>97 Don't know/Not sure	IPAQ4h<br>IPAQ4m
Think about the time you spent walking in the last 7 days. This includes at work and at home, walking to travel from place to place, and any other walking that you have done solely for recreation, sport, exercise, or leisure.			Munnaku omusanvu eziyise, lowooza ku budde bwewamala ng'otambula. Muno mulimu ng'oli kumulimu, awaka, okutambula okuva mukifo ekimu okudda mukirala, n'okukola ebyemizanyo, okukola dduyiro mu budde bwo obweddembe.	
Q.97	During the last 7 days, on how many days did you walk for at least 10 minutes at a time?	Mu nnaku omusanvu eziyisse, naku mekka zotambudde wakiri eddakiika kumi omulundi gumu nga toyimiriddemu?	|__| days [range 0-7] per week<br>**If 0, SKIP TO Q.99**	IPAQ5

Q.98	How much time did you usually spend walking on one of those days?	Watwalanga budde ki ngotambula mu lunaku olumu kwezo?	|__|__| hours per day |__|__| minutes per day<br>97 Don't know/Not sure	IPAQ6h<br>IPAQ6m

Nandyagandde okukubuuzayo ebibuuzo ebikwata ku kufumba.

#					
**Cooking Practices**					
Q.99	In order of frequency of use, what are the three main types of fuel that your household uses for cooking?	Nga otandika ne kyemusinga okufumbisa, biki ebisatu amaka go byegasinga okukozesa okufumba?	Wood …………01<br>Charcoal……….02<br>Dung ……….03<br>Liquified petroleum gas (LPG)… 04<br>Natural Gas(bio)………05<br>Kerosene………06<br>Electricity………07<br>Crop Waste………08<br>Other…………09<br>No additional response 88<br>Do not cook 98. Q.102<br>Specify_____	|__|__
__	__
__	__		COOKPRAC1<br>COOKPRAC2<br>COOKPRAC3<br>COOKPRACS
Q.100	Does your household primarily cook indoors or outdoors?	Mu maka mwobeera, musinga kufumbira munda oba wabweru?	Indoors…….1<br>Outdoors……2	|__|	COOKPRAC4
Q.101	Are you the primary person responsible for cooking in your household?	Mumaka mwobera, gwe avunanyizibwa ku kufumba?	Yes…………1<br>No…………2	|__|	COOKPRAC5
**Diet**					
The next questions ask about foods and drinks. I have cards here that shows you some examples. As you answer these questions please think of a typical week in the last year.			Ebibuuzo ebiddako bikwata ku bibala n'enva endiirwa zotera okulya.<br>Nina ekipande ekiraga ebimu kubika byebibala nenva endiirwa ebiiri mukitundu. Buli kifananyi ekiraga ebintu ebyandibadde biribwa, ngoddamu ebibuuzo bino<br>mwattu lowooza ku wiiki emu mu myezi ekkumi nebiri egiyise?		
Q.102	In a typical week, on how many days do you eat fruit? (SHOW CARD)	Okutwalira awamu mu wiiki, nnaku mmeka z'olyamu ebibala (show card)	|__|__| number of days [range 0-7]		FRUIT
Q.103	In a typical week, on how many days do you eat vegetables? (SHOW CARD)	Okutwalira awamu mu wiiki, nnaku mekka z'oliddemu enva endiirwa? (show card)	|__|__| number of days [range 0-7]		VEG

Q.104	In a typical week, on how many days do you eat processed food, e.g. packaged snacks? (SHOW CARD)	Okutwalira awamu mu wiiki, nnaku mmeka z'olyamu eby'okulya ebikoleddwawo/obumpankyumpankyu	|__|__| number of days [range 0-7]	SNACKS
Q.105	In a typical week, on how many days do you eat food that has been fried in oil or fat, e.g. mandazi, chapati, rolex, fried meats and fish? (SHOW CARD)	Okutwalira awamu mu wiiki, nnaku mmeka z'olyamu ebintu ebisikiddwa mu butto oba omuzigo okugeza mandazi, chapati ,eby'enyanja ebisike, enyama	|__|__| number of days [range 0-7]	FRIEDFOOD
Q.106	In a typical week, on how many days do you drink sugar-sweetened beverages, e.g. sodas, pre-packed juices, sweetened fruit drinks? (SHOW CARD)	Okutwalira awamu mu wiiki, nnaku mmeka z'onyweddemu eby'okunywa ebirimu sukali, soda oba juice omupakingire ddala	|__|__| number of days [range 0-7]	BEVERAGE

#					
With the next questions, we would like to learn more about salt in your diet.			Ebibuuzo ebiddako twagala okwongera okumanya ebikwata ku mmunyo mubyolya		
Q.107	How often do you add salt, salty seasoning or a salty sauce to your prepared foods	Emirundi gyenkanawa gyoyongera omunyo, oba ebirungo ebirimu omunyo mu byokulya ebimaze okufumbibwa?	Always………1<br>Often…………2<br>Sometimes…3<br>Rarely………4<br>Never………5<br>Don't know…7	Buli kiseera 1<br>Ebiseera ebisinga obungi 2<br>Luusi na luusi 3<br>Tekitera kubaawo 4<br>Tekibererawo ddala 5<br>Don't know 7	|__|SALT
**Smoking**					
Q.108	Have you ever smoked any of the following tobacco products?	Wali onyweddeko kubintu bino wammanga ebiva mu taaba?	Yes No<br>Cigarettes(Sigala) 1 2<br>Taaba 1 2<br>Pipes(Emindi) 1 2<br>**If all are 2, then skip to Q.112.**		CIGAEVER<br>TABAEVER<br>PIPEEVER
Q.109	Do you currently smoke any of the following tobacco products?	Mukiseera kino onywa ku bintu	Yes No<br>Cigarettes(Sigala) 1 2<br>Taaba 1 2<br>Pipes(Emindi) 1 2		CIGACUR<br>TABACUR<br>PIPECUR

		bino wammanga ebiva mu taaba?			
Q.110	If smoked cigarette ask else skip to Q. 111<br><br>On a typical day, when you smoke (or smoked), how many cigarettes do you smoke?	Mulunaku olumu lwonywa oba lwoba onywedde sigala onywa/wanywanga a eminwe emmeka?	|__|__|		SMOKENUM
Q.111	About how many years have you smoked in total?	Emyaka emeka nga ogigasse gyonyweredde sigala/gyewanywera sigala , taaba oba emindi?	|__|__| ***If les than a year, code 96***		SMOKEYRS
**Alcohol**					
Q.112	How often do you have a drink containing alcohol?	Emirundi gyenkanawa gyonywa ekintu ekirimu omwenge?(prompted)	Never 1<br><br>Monthly or less 2<br><br>2-4 times a month 3<br><br>2-3 times a week 4<br><br>4 or more times a week 5	***Never (no alcohol in the past 12 months) [skip to Q.115]***<br>|__| Tonywa ngako oba tonyweddeko mumyezi 12 eyise….. 1<br>omulundi gumu mu mwezi oba obutaweera…2<br>emirundi 2 okutuka kwe 4 mu mwezi…. 3<br>emirundi 2 okutuka kwe'essatu mu wiiki…. 4<br>Emirundi 4 oba okusingawo mu wiiki…5	ETOH1

Q.113	How many standard drinks containing alcohol do you have on a typical day? (show card)	Olunaku lwoba onywedde omwenge byakunywa bimeka ebirimu ebitamiza byonywa?(prompted)	1 or 2… 1<br>3 to 4……2<br>5 to 6…….3<br>7 to 9…….4<br>10 or more…5	|__|	ETOH2
Q.114	How often do you have six or more drinks on one occasion?	Emirundi gyenkanawa gyofuna eby'okunywa mukaaga oba okusingawo omulundi gumu.	Never 1<br>Daily or almost daily 2<br>Weekly 3<br>Monthly 4<br>Less than monthly 5	Tekibangawo 1<br>|__| Buli lunaku oba kumpi buli lunaku 2<br>Wakiri omulundi gumu mu wiiki 3<br>Wakiri omulundi gumu mu mwezi 4<br>Emyezi egimu mu mwaka naye si buli mwezi 5	ETOH3

#				
**Quality of Life**				
Under each heading, please tick the ONE box that best describes your health TODAY.		Kubino byengenda okukusomera njagala ombulire ekisinga okunyonyola embeera y'obulamu bwo bweri olwalero.		
Q.115	O I have no problems walking about.<br>O I have some problems in walking about.<br>O I am confined to bed.	Sirina buzibu mukutambula tambula….1<br>Ninamu ko obuzibu obutonotono mu kutambula tambula ……. 2<br>Sisobolera ddala kutambula……3	|__|	QOLWALK
Q.116	O I have no problems washing or dressing myself.<br>O I have some problems washing or dressing myself.<br>O I am unable to wash or dress myself.	Sirina buzibu mukwenaaza oba okweyambaza 1<br>Ninamu ko obuzibu obutonotono mukwenaaza oba okweyambaza 2<br>Sisobolera ddala kwenaaza oba okweyambaza 3	|__|	QOLDRESS
Q.117	O I have no problems doing my usual activities.<br>O I have some problems doing my usual activities.<br>O I am unable to do my usual activities.	Sirina buzibu mu kukola mirimu gyange egyabulijjo 1<br>Ninamu ko obuzibu butonotono mu kukola emirimu gyange egyabulijjo 2<br>Sisobolera ddala kukola mirimu gyange egyabulijjo 3	|__|	QOLACT
Q.118	O I have no pain or discomfort.	Sirina wannuma oba okuwulira obubi 1<br>Ninamu ko obulumi oba okuwulira obubi kutonotono 2	|__|	QOLPAIN

	O I have moderate pain or discomfort.<br>O I am in extreme pain or discomfort.	Nina obulumi bwamaanyi nnyo oba mpulira bubi nnyo nnyo 3			
Q.119	O I am not anxious and depressed.<br>O I am moderately anxious and depressed.<br>O I am extremely anxious and depressed.	Siri mweralikirivu era siri mwenyamivvu 1<br>Ninamu ko obweralikirivvu era n'obwenyamivvu butonotono 2<br>Nina obwerarikirivvu n'obwenyamivvu bwamaanyi nnyo 3		|__|	QOLANX
Q.120	We would like to know how good or bad your health is TODAY.<br>-This scale is numbered from 0 to 100.<br>-100 means the best health you can imagine.<br>0 means the worst health you can imagine.<br>-Mark an X on the scale to indicate how your health is TODAY.<br>-Now, please write the number you marked on the scale in the box below.	Twandyagadde okumanya embeera y'obulamu bwo bweri kati? nnungi oba mbi *(provide a scale)*<br>Akapimo kano katekeddwako ennamba okuva ku zeero paka ku kikumi (0-100)<br>-kikumi (100) kitegeeza obulamu obulungi enyo bwosobola okulowooza ko<br>-zeero (0) kitegeeza obulamu obubi enyo bwoyinza okulowooza ko<br>-Ng'okozesa akapimo kano laga obulamu bwo bwebuli olwaleero<br>-kati wandiika ennamba gy'olambye kukapimo kano mu ka boxi wammanga		|__|__|__|	QOL100
**TB HISTORY/SYMPTOMS**					
Q.121	Have you ever been diagnosed with TB before?	Wali okebedwako n'ozulibwa ng'olina obulwadde bwa kafuba (TB)?	Yes………1<br>No………2<br>skip to Qn 123<br>DK/NR….7---skip to Qn 123	|__|	TBDX
Q.121	Have you ever been treated for TB before?	Wali ofunyeeko obujanjabi bw'obulwadde bwakafuba (TB)?	Yes………1<br>No…………2<br>DK/NR……7	|__|	TBTX
	•				
**History of NCDs and Health Care Utilization**					
Q.123	Before today, have you ever been told by a doctor or other health worker that you have raised blood pressure or hypertension?	Ngojjeko olwaleero,wali otegezeddwako Dokita oba omusawo omutendeke nti	Yes…………1<br>No…………2---→127<br>DK/NR……7--->127	|__|	HTNTOLD

		olina obulwadde bwa pulesa?			
Q.124	In the past two weeks, have you taken any drugs (medication) for raised blood pressure prescribed by a doctor or other health worker?	Munnaku kkumi nanya (14) eziyise omizeeko ku ddagala lyonna erijjanjaba pulesa nga Dokita oba omusawo omutendeke yenna yalikuwandiikidde? ***(to consult)***	Yes………..1<br>No……2-skip to Q.126<br>DK/NR…-skip to Q.126	|__|	HTNMED
Q.125	Where does this doctor/health worker who asked you to take this medication work at (name, location)?	Omusaawo/dokita eyakugamba oba eyakuwa eddagala/obujjanjabi akolera wa?	|__|__|__| Health unit code<br><br>|_______|if not from health unit, then write out name and location	|__|	HTNLOCD<br>HTNLOC<br>HTNLOCS
Q.126	Are you currently taking any herbal or traditional remedy for your raised blood pressure?	Mukiseera kino okozesa eddagala lyekinnansi okukujjanjaba obulwadde bwa pulesa?	Yes……………1<br>No…………2<br>DK/NR………7	|__|	HTNTRAD
Q.127	Have you ever been told by a doctor or other health worker that you have raised blood sugar or diabetes?	Wali otegezeddwako Dokita oba omusawo omutendeke nti olina obulwadde bwa sukaali oba sukali mungi mu musaayi gwo?	Yes………..1<br>No……2___-skip to 131<br>DK/NR……7--→Q.131	|__|	DIABTOLD
Q.128	In the past two weeks, have you taken any drugs (medication) for diabetes prescribed by a doctor or other health worker?	Munnaku kumi nannya(14) eziyise omizeeko/okozesezaako eddagala erijjanjaba sukaali nga Dokita oba omusawo omutendeke yalikuwandikidde?	Yes……………1<br>No…2-skip to Q.130<br>DK/NR..7-skip to Q130	|__|	DIABMED
Q.129	Where does this doctor/health worker who asked you to take this medication work at (name, location)?	Omusaawo/dokita eyakugamba oba eyakuwa eddagala/obujjanjabi akolera wa?	|__|__|__| Health unit code<br><br>|_______|if not from health unit, then write out name and location	|__|	DIABLOCD<br>DIABLOC<br>DIABLOCS
Q.130	Are you currently taking any herbal or traditional remedy for your diabetes?	Mukiseera kino okozesa eddagala ly'ekinnansi okukujjanjaba obulwadde bwa sukaali?	Yes……………1<br>No……………2<br>DK/NR………7	|__|	DIABTRAD

Q.131	Have you ever been told by a doctor or other health worker that you have had a heart attack or a stroke?	**Wali otegezeddwako Dokita oba omusawo omutendeke nti olina obulwadde bwo** okulumwa mukifuba nga kizze mubwangu nga kireteddwa obulwadde bw'omutima oba okusanyalala oludda olumu olw'omubiri oba okusanyalala omubiri gwona (heart attack)?	Yes……………1<br>No……………2<br>DK/NR………7	|__|	HEARTTOLD
Q.132	Have you ever been told by a doctor or other health worker that you have respiratory condition such as asthma?	**Wali otegezeddwako Dokita oba omusawo omutendeke nti olina obulwadde bwa asthma /oluyiro?**	Yes……………1<br>No……2-→Q.135<br>DK/NR…7→Q.135	|__|	ASTHMATOLD
Q.133	Are you currently on any medicine for respiratory condition such as asthma or chronic obstructive pulmonary disease (COPD)?	**Mukiseera kino olina eddagala lyona ly'okozesa okwejjanjaba Asthma/oluyiro?**	Yes……………1<br>No……………2- skip to Q,135<br>DK/NR………7- skip to Q.135	|__|	ASTHMAMED
Q.134	Where does this doctor/health worker who asked you to take this medication work at?	Omusaawo/dokita eyakugamba oba eyakuwa eddagala/obujjanjabi akolera wa?	|__|__|__| Health unit code<br><br>|_______|if not from health unit, then write out name and location	|__|	ASTHMALOCD<br>ASTHMALOC<br>ASTHMALOCS
Q.135	How many times have you visited a doctor or health care worker in the past 12 months?	Mumyezi ekumi n'ebbiri egiyise emirundi emmeka gyogenzeeko okulaba omusawo?	|__|__| <br>If 0, skip to Q.**137**<br>DK……97		HEALTHNUM
Q.136	For what health condition(s) did you visit the doctor or health care worker(s) in the	Mumyezi ekumi n'ebbiri egiyise buzibuki oba bulwaddeki bwewalina obwakuleetera	Yes No<br>HIV 1 2<br>Hypertension 1 2<br>HeartDisease 1 2<br>Diabetes 1 2<br>Respiratory Condition (Asthma, COPD) 1 2<br>Malaria 1 2	|__| |__| |__| |__| |__| |__| |__| |__|	HIV12M<br>HTN12M<br>HEART12M<br>DIAB12M<br>COPD12M<br>MALA12M<br>STI12M<br>PREG12M<br>INJURY12M

	past 12 months? (Select all that apply, unprompted)	okugenda okulaba omusawo?	STI 1 2<br>Pregnancy 1 2<br>Injury 1 2<br>Diarrhea 1 2<br>Fever 1 2<br>Other|__________ 1 2	|__| |__| |__| |__|	DIARRH12M<br>FEVER12M<br>OTH12M<br>OTH12MSP
**Asthma and Chronic Bronchitis**					
Q.137	Have you had any wheezing in the past 12 months?	Mumyezi ekumi n'ebbiri egiyise, ofunyeko obukaaba mukifuba?	Yes……………1<br>No……………2<br>DK/NR………7	|__|	ASTHMA
Q.138	Have you had any productive cough (with phlegmn) for 3 consecutive months, 2 years in a row?	Ofunyeko okukolola nga ekikolondorwa kivaayo okumala emyezi esatu egiddiringana mu bbanga eryemyaka ebiri?	Yes……………1<br>No……………2<br>DK/NR………7	|__|	BRONCHITI<br>S

**If ever interviewed in R20 and not a Vicinity participant skip to Q.192**	**ST. GEORGE'S RESPIRATORY QUESTIONAIRE (SGRQ)**

This questionnaire is designed to help us learn much more about your breathing and how it affects your life.				Ebibuuzo bino bikoleddwa okutuyamba okumanya<br>engeri okussakwo gyekukutataganya n'engeri gyekukosamu obulamu bwo. Tubikozesa okuzuula<br>ebintu ebyenjawulo mu bulwadde bwo ebisinga<br>okukuletera obuzibu ng'ojjeko e'kyaba dokita oba<br>abasawo byebalowoozanti bwebuzibu bwo.<br>Bambi wuliriza bulungi n'obwegendereza era obuuze kyonna kyonooba totegedde.		
**Q.140**	***Would you describe your current health as Very good, good, fair, poor, or very poor?***	Wandigambye nti embeera y'obulamu bwo nungi nnyo , nungi,<br>BwetyoBwetyo,<br>mbi,<br>mbi nnyo.	Very good 1<br>Good 2<br>Fair 3<br>Poor 4<br>Very poor 5	Nnungi nnyo..1<br>Nnungi …. 2<br>BwetyoBwetyo.. 3<br>Mbi …. 4<br>Mbi nnyo… 5	|__|	**SG1**
**Questions about how much chest trouble you have had over the past 03 months**			**Ebibuuzo bino bikwata kungeri gyotataganyiziddwa mu kifuba mu banga eryemyezi esaatu egiyise.**			
Q.141	over the past 3 months, I have coughed	Mu myezi esaatu egiyise, okolodde	**Most days a week 1**<br>**Several days a week, 2**<br>**A few days a month, 3**<br>**Only with chest 4 infections**<br>**Not at all 5**	Ennaku ezisinga mu wiiki… 1<br>Ennaku eziwerako mu wiiki …2<br>Ennaku ntono mu mwezi… 3<br>Obulwadde bubadde mukifuba mwoka….4<br>Tekibaddewo nakatono… 5	|__|	**SG2**
Q.142	Over the past 3 months, I have brought up phlegm (sputum)	Mu myezi esaatu egiyise okolodde novamu ekikolondolwa:	**Most days a week 1**<br>**Several days a week 2**<br>**A few days a month 3**<br>**Only with chest 4**	Ennaku ezisinga mu wiiki…1<br>Ennaku eziwerako mu wiiki…. 2<br>Ennaku ntono mu mwezi…. 3	|__|	**SG3**

			**infections,<br>Not at all 5**	Obulwadde bubadde mukifuba mwoka…..4<br>Tekibaddewo nakatono…. 5		
Q.142	Over the past 3 months, I have had shortness of breath	Mu myezi esaatu egiyise, obadde n'obuzibu mu kussa:	**Most days a week 1<br>Several days a week, 2<br>A few days a month, 3<br>Only with chest 4 infections<br>Not at all 5**	Ennaku ezisinga mu wiiki … 1<br>Ennaku eziwerako mu wiiki… 2<br>Ennaku ntono mu mwezi…. 3<br>Obulwadde bubadde mukifuba mwoka…..4<br>Tekibaddewo nakatono…..5	|__|	**SG4**
Q.143	Over the past 3 months, I have had attacks of wheezing	Mu myezi esaatu egiyise ofunyeko obukaaba mukifuba ng'ossa:	**Most days a week 1<br>Several days a week, 2<br>A few days a month, 3<br>Only with chest 4 infections<br>Not at all 5**	Ennaku ezisinga mu wiiki…. 1<br>Ennaku eziwerako mu wiiki …..2<br>Ennaku ntono mu mwezi… 3<br>Obulwadde bubadde mukifuba mwoka….4<br>Tekibaddewo nakatono…. 5 _	|__|	**SG5**
Q.144	**During the past 3 months how many severe or very unpleasant attacks of chest trouble have you had? *Please tick in one***	Mubanga ery'emyezi esaatu egiyise emirundi emeka gy'ofunyeko obuzibu obwamanyi mukifuba?	More than 3 attacks 1<br>3 attacks 2<br>2 attacks 3<br>1 attack 4<br>No attacks 5	Emirundi egissuka mwe'saatu…. 1<br>Emirundi esaatu… 2<br>Emirundi ebiiri..3<br>Omulundi gumu…4<br>Tokifunyeko… 5____Q 146	|__|	**SG6**
Q.145	**How long did the worst attack of**	Obuzibu mukifuba obwasinga okuba obwamaanyi	A week or more 1	Wiiki namba oba okusingawo… 1	|__|	**SG7**

	**chest trouble last? *Please tick in one* (go to SECTION 7 if you had no severe attacks)**	bwatwala banga ki? (Go to SECTION 7 if you had no severe attacks)	3 or more days 2<br>1 or 2 days 3<br>Less than a day 4	Ennaku ssatu oba okusingawo… 2<br>Olunaku lumu oba bbiri….. 3<br>Olunaku telwawera…. 4		
Q.146	**Over the past 3 months, in an average week, how many good days (with little chest trouble) have you had? *Please tick in one***	Mu myezi essatu egiyise, okutwalirawamu mu wiiki, nnaku mmeka zobadde n'obuzibu mukifuba obutono?	No good days 1<br>1 or 2 good days 2<br>3 or 4 good days 3<br>Nearly every day is good 4<br>Every day is good 5	Tewabaddewo lunaku lulungi..1<br><br>Olunaku lumu oba bbiri… 2<br>Ennaku ssatu oba nnya… 3<br>Kyenkana buli lunaku luba lulungi …4<br>Buli lunaku luba lulungi…. 5	|__|	**SG8**
Q.147	**If you have a wheeze, is it worse in the morning? *Please tick in one***	Bwofuna obukaaba mu kifuba nga ossa, buyitirira/bweyongera mubudde obw'okumakya?	Yes 1<br>No 2	|__|	**SG9**	
Section 1						
Q.148	**How would you describe your chest condition? (please tick one)**	Oyinza kunyonyola otya embeera y'ekifuba kyo?	The most important problem I have 1<br>Causes me quite a lot of problems 2<br>Causes me a few problems 3<br>Causes no problem 4	Kyekizibu kyosinga okubera nakyo..1 |__| Kikuleetera obuzibu bwamaanyi … 2<br>Kikuleetera obuzibu butono…. 3<br>Tewali buzibu bwekikuleetera… 4	|__| SGA1	
Q.151	If you have ever had paid employment	Bwoba nga wali obaddeko nomulimu ogukusasuza ssente	My chest trouble made me stop work 1	Obuzibu bwekifuba bwakuletera	|__| SGA3	

			My chest trouble interferes with my work or made me change my work 2<br>My chest trouble does not affect my work 3	okulekerawo okukola ….. 1<br>Obuzibu bwekifuba bwakutataganya mu mirimugyo oba bwakuletera okukyusa omulimo gwo…2<br>Obuzibu bwekifuba kyo tebukosa mirimo gyo…..3		
**SECTION 2**						
**Questions about what activities usually make you feel breathless these days (please tick in each box that applies to you these days)**				Ebibuuzo ebikwata ku bintu byokola ebikuletera okuwulira ng'olina obuzibu mu kussa/okuziyira ennaku zino<br>Nsaba onziremu oba kitufu oba sikitufu ku bino<br>byengenda okukusomera		
				**True (Kitufu)**	**False (Sikitufu)**	
Q.152	Sitting or lying still	Okutuula oba okwebaka awamu nga tovaawo		**1**	**2**	**SGB1**
Q.153	Getting washed or dressed	Okunaaba oba okwambala		**1**	**2**	**SGB2**
Q.155	Walking around the home	Okutambulatambulirako awaka		**1**	**2**	**SGB3B**
Q.154	Walking outside on the level	Okutambulira ku museetwe oba awaterevu		**1**	**2**	**SGB3**
Q.156	Walking up a flight of stairs	Okulinnya amaddaala		**1**	**2**	**SGB4**
Q.157	Walking up hills	Okulinnya akasozi		**1**	**2**	**SGB5**
Q.158	Playing sports or games (eg football, netball, Ludo, etc)	Okuzanya obuzanyo/ emizanyo (okugeza okubaka omupiira, okusamba omupiira, Ludo)		**1**	**2**	**SGB6**
**SECTION 3**						
	Some more questions about your cough and	Ebibuuzo bino ebirala bikwata kukukolola n'obuzibu mukussa/okuziyiira ennaku zino.		**True (Kituufu)**	**False (Sikituufu)**	

	breathlessness these days.	Nsaba onziremu oba kitufu oba sikitufu ku bino byengenda okukusomera			
Q.160	My cough hurts	Okukolola kukukosa /kukulumya	**1**	**2**	**SGC1**
Q.161	My cough makes me tired	Okukolola kukukooya	**1**	**2**	**SGC2**
Q.162	I am breathless when I talk	Ofuna obuzibu mu kussa /okuziyira ngoyogera	**1**	**2**	**SGC3**
Q.163	I am breathless when I bend over	Oziyira oba ofuna obuzibu mu kusa ng'okutamyeko	**1**	**2**	**SGC3B**
Q.164	My cough or breathing disturbs my sleep	Okukolola oba engeri gyossamu kutataganya okwebaka kwo	**1**	**2**	**SGC4**
Q.165	I get exhausted easily	Okoowa mangu	**1**	**2**	**SGC5**
**SECTION 4**					
	Questions about other effects that your chest troubles may have on you these days.	Ebibuuzo bino bikwata ku bintu ebirala ebiyinza okuba nga bivudde ku buzibu bwekifuba munnaku zino<br><br>Nsaba onziremu oba kitufu oba sikitufu ku bino byengenda okukusomera	**True (kitufu)**	**False (Sikitufu)**	
Q.166	My cough or breathing is embarrassing in public	Okukolola oba obuzibu mu kussa kukuswaza ngo'limu bantu	**1**	**2**	**SGD1**
Q.167	My chest trouble is nuisance to my family, friends or neighbours	Obuzibu mukifuba kizibu eri abengandazo, mikwanogyo oba abemirirwano	**1**	**2**	**SGD2**
Q.168	I get afraid or panic when I cannot get my breath	Otya bwoba tosobola kussa	**1**	**2**	**SGD3**
Q.169	I feel that I am not in control of my chest problem	Owulira ng'obulwadde bw'ekifuuba kyo tobusobola	**1**	**2**	**SGD3B**
Q.170	I do not expect my	Tosuubira kifuba kyo kutereera	**1**	**2**	**SGD4**

	chest to get any better				
Q.171	I have become frail or an invalid because of my chest	Onafuye oba ofuuse owokwegendereza olw'ekifuba kyo	**1**	**2**	**SGD5**
Q.172	Exercise is not safe for me	Okukola dduyiro kyabulabe gyoli	**1**	**2**	**SGD6**
Q.173	Everything seems too much of an effort	Buli Kintu kyona kyokola kirabika nga ekyetaaga okusaamu amaanyi amangi	**1**	**2**	**SGD7**
**SECTION 5**<br>**Q.171**<br>**Probe to see if respondent is taking any medication for chest problem** Yes 1 |__| If not receiving medication go straight to Section 6 No 2<br>N/A (no chest problem) 8					**CHESTPROB**
	**Question about medication if you are receiving no medication go straight to section 6.**	Ebibuuzo bino bikwata ku ddagala lyo lyomira/okozesa olw'obuzibu bw'ekifuba<br><br>Nsaba onziremu oba kitufu oba sikitufu ku bino byengenda okukusomera	**True (Kitufu)**	**False (Sikitufu)**	
Q.174	My medication does not help me very much	Eddagala lyo terilina nnyo kyerikuyamba	**1**	**2**	**SGE1**
Q.175	I get embarrassed using my medication in public	Oswala okukozesa eddagala lyo nga oli mu bantu	**1**	**2**	**SGE2**
Q.176	I have unpleasant side effects from my medication	Ofuna obuzibu nga buva ku ddagala lyo	**1**	**2**	**SGE3**
Q.177	My medication interferes with my life a lot	Eddagala lyo litataganya nyo obulamu bwo	**1**	**2**	**SGE4**
**SECTION 6**					
	**These are questions about how your**	Ebibuuzo bino bikwata kungeri ebintu byokola gyebiyinza okuba nga bikoseddwa olwengeri gyossamu	**True (Kitufu)**	**False (Sikitufu)**	

	**activities might be effected by your breathing.**	Nsaba onziremu oba kitufu oba sikitufu ku bino byengenda okukusomera			
Q.178	I take long time to get washed or dressed	Otwala obudde buwanvu okunaaba oba okwambala	**1**	**2**	**SGF1**
Q.179	I cannot take a bath or shower, or I take a long time	Tosobola kunaaba oba otwala obudde buwanvu okunaaba	**1**	**2**	**SGF2**
Q.180	I walk slower than other people, or I stop for rests	Otambula mpola bwogerageranya ku bantu abalala oba oyimiriramu okuwumulamu bw'oba otambula	**1**	**2**	**SGF3**
Q.181	Jobs such as housework take a long time, or I have to stop for rest	Emirimo j'ewaka gikutwalira obudde buwanvu oba Ojiyimirizamu oluvanyuma nogenda mumaaso okujikola	**1**	**2**	**SGF4**
Q.182	If I walk up one flight of stairs, I have to go slowly or stop	Bwoba otambula ngolinya amaddala okutuuka kumwaliiro ogudako/kunyumba eddako oba olina okugenda empola oba okuyimiriramu.	**1**	**2**	**SGF5**
Q.183	If I hurry or walk fast, I have to stop or slow down	Bwo'tambula ngoyanguwa, oba oyina okuyimiriramu oba okukendeezako	**1**	**2**	**SGF6**
Q.184	My breathing makes it difficult to do things such as walk up hills, carrying things Upstairs, Light gardening such as weeding, dance, Play bowl or play golf	Engeri gyossamu ekifula kizibu okukola ebintu okugeza okutambula ng'olinya akasozi, okwambusa ebintu waggulu mu nyumba eyakalina, okulima okutonotono okugeza okukoola, okuzinamu/okubibya, oba okuzanya golf	**1**	**2**	**SGF7**
Q.185	My breathing makes it difficult to do	Engeri gyossamu ekifula kizibu okukola ebintu okugeza okusitula ebizito, okulima, okudduka oba	**1**	**2**	**SGF9**

	things such as heavy loads, dig the garden Shovel snow, jog or walk at 5 miles per hour, play tennis or swim	okutambula mayilo taano(5miles) buli saawa,<br>okuzanya tennis oba okuwuga			
Q.186	My breathing makes it difficult to do things such as very heavy manual work, Run, cycle swim fast or play competitive sport	Engeri gyossamu ekifula kizibu okukola ebintu okugeza okukola ebintu ebyeetaaga amanyi amangi, okudduka, okuvuga eggali, okuwuga mubwangu<br>oba okuzanya emizanyo egyokuvuganya	**1**	**2**	**SGF11**
**SECTION 7**					
	We would like to know how your chest usually affects your daily life.	Twandyagadde okumanya engeri ekifuba kyo gyekitera okukosa embeera y'obulamu bwo eyabulijjo Nsaba onziremu oba kitufu oba sikitufu ku bino byengenda okukusomera	**True (KITUFU)**	**False (Sikitufu)**	
Q.187	I cannot play sports or games	Tosobola kuzanya mizanyo	**1**	**2**	**SGG1**
Q.188a	I cannot go out for entertainment or recreation	Tosobola kugenda kwesanyusamu	**1**	**2**	**SGG2**
Q.188b	I cannot go out of the house to do the shopping	Tosobola kuva munyumba kugenda kugula bintu<br>kumadduuka	**1**	**2**	**SGG3**
Q.189	I cannot do housework	Tosobola kukola mirimo gyawaka	**1**	**2**	**SGG4**
Q.190	I cannot move far from my bed or chair	Tosobola ku genda wala kuva ku kubuliri bwo oba mu ntebe	**1**	**2**	**SGG5**

	**Here is a list of other activities that your chest troubles may prevent you doing. (You do not have to tick these, they are just to remind of way in which your breathlessness may affect you):**<br>Going for walk or walk the dog<br>Doing things at home or in the garden<br>Sexual intercourse<br>Going out to church, pub, or place of entertainment<br>Going out in bad weather or into smoky rooms<br>Visiting family or friend or playing with children	Bino wammanga bye bintu ebirala ebiyinza okukulema okukola olw'obuzibu bwolina mukifuba. Ebintu bino bikujjukizamu engeri obuzibu mukussa gyebuyinza okukossamu.<br>Okugenda okutambula oba okutambuza embwa<br>Okukola ebintu awaka oba okugenda okulima<br>Okwegatta	Okugenda mu sinzizo, mu baala, ekiddula oba ebifo ebisanyukirwamu<br><br>Okugenda ebweru mumbeera y'obudde embi oba mubisenge omuli omuka<br>Okukyalira ab'engandazo oba mikwanogyo oba okuzanya n'abaana	

		Kati njagala ombulire ku bino, kiki kyolowooza ekisinga okunyonyola obulungi engeri ekifuba gyekikukossamu	
Q.191	It does not stop me doing anything I would like to do 1<br>It stops me doing on or two things I would like to do 2<br>It stops me doing most of the things I would like to do 3<br>It stops me doing everything I would like to do 4	Tekikulemesa kukola kintu kyona kyewandyagadde kukola 1<br>Kikulemesa okukola ekintu kimu oba bibbiri byewangyagadde okukola 2 Kikulemesa okukola ebintu ebisinga byewandyagadde okukola 3<br>Kikulemesa okukola ebintu byona byewangyagadde okukola 4	|__| SGEND

			**Nandyagadde okukubuzaayo ekibuuzo ekikwata kukomolebwa**		
Q.192	Are you circumcised?	Oli mukomole?	Yes 1<br>No 2	|_ _|	CIRCUM

Migration and Mobility Module

Q.193	**In the past 12 months, have you spent any nights away from your current residence ?**	**Mu myezi kumi n'ebbiri egiyise osuzeeko mukifo ekirala ekitali kyobeeramu kati?**	Yes 1<br>No 2..<br>**If 2 skip to Q.195a**	|___|	NIGHTWAY

Q.194 In the past 12 months, how many nights did you spend in Kampala?

Mu myezi kumi n'ebbiri egiyise biro bimeka by'osuzeeko e Kampala?

|__|__|DDNWKLA
|__|__|WWNWKLA
|__|__|MMNWKLA

Q.195 In the past 12 months, how many nights did you spend in Masaka?

Mu myezi kumi n'ebbiri egiyise biro bimeka by'osuzeeko e Masaka?

|__|__|DDNWMSK
|__|__|WWNWMSK
|__|__|MMNWMSK

**TIMENDED |__|__|:|__|__|am/pm END_TIME}**
Team Leader #|__|__|__| {TEAMLEAD} Editor #|__|__|__||__|{EDITOR} QC# |__|__|__||___|{**QC**}<br>Data entry clerk #|__|__|__| {DATACLERK} {DE DATE } ____/___/___{dd mm yyyy}
**------------>******(END ACTIVITIES)******<------------**

COMPUTER ID | **PLACE LABNO HERE** | **CURRENT ID**

**PARTNER IDENTIFICATION**			
	PLACE COMP_ID HERE	**PLACE LABNO HERE**	STUDY_ID
	INT DATE	___/___/20__ dd mm yyyy	INT_DATE
	VISIT #	**|R|2|2|**	VISITNO

	**Current ID**	|__|__|__|__|__|__|__|__|__|__|__|__|__|__|	CURR_ID
	Super Cluster #	|__|__|__|	REGION
	Community #	|__|__|__|	COMM_NUM
	HH #	|__|__|__|__|	HH_NUM
	Member #	|__|__|__|	MEMBER_NUM

INTERVIEWER: FOR ALL RESPONDENTS IN MARRIAGE OR CONSENSUAL UNION TRY TO OBTAIN INFORMATION THAT WILL ALLOW US TO LINK THE INDIVIDUAL TO HIS MARRIAG OR CONSENSUAL PARTNERS WITHIN THE SAME HOUSEHOLD OR WITHIN THE SAME COMMUNITY. PAY ATTENTION TO CASES OF POLYGAMY WHERE WIVES MAY BE LIVING WITHIN ONE HOUSE OR IN DIFFERENT HOUSEHOLDS. USE ADDITIONAL PAGES IF >3 PARTNERS. FOR SAME HOUSEHOLD PARTNERS, TRY TO FIND THE MEMBER # FROM THE HOUSEHOLD CENSUS LISTING. FOR PARTNERS IN DIFFERENT HOUSEHOLDS, THIS INFORMATION WILL HAVE TO BE COMPLETED LATER.

**Partner 1**	**BLOCK # |__|**			**BLOCKNO1**
I would like to clearly know, do you have a wife/partner that you are staying within this household? **(If Yes,)** Names tell me all the possible names by which she is called.	Nandyagadde ntegeere bulungi, olina omukyala gw'osula naye mu maka muno?<br>**(If yes,)** Amanya ge y'ani? Mbuulira amannya gonna gebatera okumuyita			
Current ID # for partner 1:	|___|____|__|/|___|___|___|/|__|___|__|__/|__|__|__| (Supercluster / Community / Household / Member)			P1_CURR_ID
Does she normally stay in this community?	**(If no,)**Atera kubeera mu kitundu kino?	Yes ... 1 **|__|**<br>No... 2<br>Out spouse....3		
What are her names?<br>______________________<br>______________<br>**SUPERVISOR: TRY TO LOCATE ID FROM HOUSEHOLD CENSUSES IN COMMUNITY**.<br>Current ID # for partner 1:<br>|___|____|__|/|___|___|___|/|__|___|__|__/|__|__|__| (Supercluster / Community / Household / Member)		Erinya lye y'ani?<br>______________________<br>______________<br>**EDITOR: TRY TO LOCATE ID FROM HOUSEHOLD CENSUSES IN COMMUNITY**<br>**Current ID# for partner1:**<br>|___|____|__|/|___|___|___|/|__|___|__|__/|__|__|__<br>(Supercluster / Community / Household / Member)		
**(If not)** Can you tell me where she stays?<br>______________________<br>______________		**(If not)** Osobola okumbulira wa gy'abeera?<br>______________________		

#			
	**Partner 2**	**BLOCK # |__|**	**BLOCKNO2**
I would like to clearly know, do you have a /wife/partner that you are staying within this household?	Nandyagadde ntegeere bulungi, olina omukyala gw'osula naye mu maka muno?<br>**(If yes,)** Amanya ge y'ani? Mbuulira amannya gonna gebatera okumuyita		

<table>
<tr><td>(If Yes,) Names tell me all the possible names by which she is called.</td><td colspan="3"></td></tr>
<tr><td colspan="4"></td></tr>
<tr><td>Current ID # for partner 1:</td><td colspan="2">|___|____|__|/|___|___|___|/|__|___|__|__/|__|__|__|
(Supercluster / Community / Household / Member)</td><td>P1_CURR_ID</td></tr>
<tr><td>Does she normally stay in this community?</td><td>(If no,)Atera kubeera mu kitundu kino?</td><td>Yes ... 1|__|
No... 2
Out spouse....3</td><td></td></tr>
<tr><td colspan="2">What are her names?
____
SUPERVISOR: TRY TO LOCATE ID FROM HOUSEHOLD CENSUSES IN COMMUNITY.
Current ID # for partner 1:
|___|____|__|/|___|___|___|/|__|___|__|__/|__|__|__|
(Supercluster / Community / Household / Member)</td><td colspan="2">Erinya lye y’ani?
____
EDITOR: TRY TO LOCATE ID FROM HOUSEHOLD CENSUSES IN COMMUNITY
Current ID# for partner1:
|___|____|__|/|___|___|___|/|__|___|__|__/|__|__|__
(Supercluster / Community / Household / Member)</td></tr>
<tr><td colspan="2">(If not) Can you tell me where he/she stays?
______________________________</td><td colspan="2">(If not) Osobola okumbulira wa gy’abeera?
______________________________</td></tr>
</table>

<table>
<tr><th>#</th><th colspan="4"></th></tr>
<tr><td></td><td><u>Partner 3</u></td><td colspan="2"><u>BLOCK # |__|</u></td><td><u>BLOCKNO3</u></td></tr>
<tr><td colspan="2">I would like to clearly know, do you have a /wife/partner that you are staying within this household?
(If Yes,) Names tell me all the possible names by which she is called.</td><td colspan="3">Nandyagadde ntegeere bulungi, olina omukyala gw’osula naye mu maka muno?
(If yes,) Amanya ge y’ani? Mbuulira amannya gonna gebatera okumuyita</td></tr>
<tr><td colspan="5"></td></tr>
<tr><td colspan="2">Current ID # for partner 1:</td><td colspan="2">|___|____|__|/|___|___|___|/|__|___|__|__/|__|__|__|
(Supercluster / Community / Household / Member)</td><td>P1_CURR_ID</td></tr>
<tr><td colspan="2">Does she normally stay in this community?</td><td>(If no,)Atera kubeera mu kitundu kino?</td><td>Yes ... 1|__|
No... 2
Out spouse....3</td><td></td></tr>
<tr><td colspan="3">What are her names?
____
SUPERVISOR: TRY TO LOCATE ID FROM HOUSEHOLD CENSUSES IN COMMUNITY.
Current ID # for partner 1:
|___|____|__|/|___|___|___|/|__|___|__|__/|__|__|__|
(Supercluster / Community / Household / Member)</td><td colspan="2">Erinya lye y’ani?
____
EDITOR: TRY TO LOCATE ID FROM HOUSEHOLD CENSUSES IN COMMUNITY
Current ID# for partner1:
|___|____|__|/|___|___|___|/|__|___|__|__/|__|__|__
(Supercluster / Community / Household / Member)</td></tr>
<tr><td colspan="3">(If not) Can you tell me where she stays?</td><td colspan="2">(If not) Osobola okumbulira wa gy’abeera?</td></tr>
</table>

______________________________ ____________	______________________________

#			
	**Partner 4**	**BLOCK # |__|**	**BLOCKNO4**
I would like to clearly know, do you have a /wife/partner that you are staying within this household? **(If Yes,)** Names tell me all the possible names by which she is called.	Nandyagadde ntegeere bulungi, olina omukyala gw'osula naye mu maka muno? **(If yes,)** Amanya ge y'ani? Mbuulira amannya gonna gebatera okumuyita		
Current ID # for partner 1:	|___|____|__|/|___|___|___|/|__|___|__|__/|__|__|__| (Supercluster / Community / Household / Member)		P1_CURR_ID
Does she normally stay in this community?	**(If no,)**Atera kubeera mu kitundu kino?	Yes ... 1 |__| No... 2 Out spouse....3	
What are her names? ______________________________ ____________ **SUPERVISOR: TRY TO LOCATE ID FROM HOUSEHOLD CENSUSES IN COMMUNITY**. Current ID # for partner 1: |___|____|__|/|___|___|___|/|__|___|__|__/|__|__|__| (Supercluster / Community / Household / Member)	Erinya lye y'ani? ______________________________ ____________ **EDITOR: TRY TO LOCATE ID FROM HOUSEHOLD CENSUSES IN COMMUNITY** **Current ID# for partner1:** |___|____|__|/|___|___|___|/|__|___|__|__/|__|__|__ (Supercluster / Community / Household / Member)		
**(If not)** Can you tell me where she stays? ______________________________ ____________	**(If not)** Osobola okumbulira wa gy'abeera? ______________________________		

SPECIMENS COLLECTED

**Purple Top**	**PT Result Req.**	**Blood**	**RT Result Req.**
Yes …….1<br>No …......2<br>NA …......8<br>Re-bled..11	Single……….........1<br>No............................2<br>Couple-Married ....3<br>Couple non-marital...4<br>AKP…………….….5<br>NA……….………...8	Yes …..….1<br>No …........2<br>NA ..….....8<br>Re-bled….11	Single ....1<br>No.............................2<br>Couple-Married …….3<br>Couple-non-marital ...4<br>AKP……………...….5<br>NA…………....….....8
Rebled Date: ____/____/______		Rebled Date: ____/____/______	

ART INITIATED

Yes ……..........1 CLINIC CODE______PATIENT'S ID_______
No ………..…..2
Declined…........3
NA..………......8
