# Supplementary material for: Seroprevalence of Rift Valley fever virus and factors associated with exposure in south-central Uganda
Source: BMC Infect Dis. 2026 Jul 7;26:1751. doi: 10.1186/s12879-026-13923-1 (PMC13628802; doi:10.1186/s12879-026-13923-1)
Supplement: Supplementary file 3 [file 12879_2026_13923_MOESM3_ESM.docx]

**Supplemental Table 1: Demographic characteristics of the 1199 RCCS participants by agrarian, trade, and fish landing site communities.**

| **Variable** | **Agrarian Community**  **n (%)** | **Trading Community**  **n (%)** | **Fishing Community**  **n (%)** | **p-value*** |
| --- | --- | --- | --- | --- |
| **Total** | 400(100) | 399(100) | 400(100) |  |
| **Sex** | | | | |
| Male | 198(49.5) | 166(41.6) | 228(57.0) | **<0.001** |
| Female | 202(50.5) | 233(58.4) | 172(43.0) |  |
| **Age (years)** | | | | |
| 15-24 | 163(40.8) | 174(43.6) | 112(28.0) | **<0.001** |
| 25-34 | 110(27.5) | 112(28.1) | 156(39.0) |  |
| ≥35 | 127(31.8) | 113(28.3) | 132(33.0) |  |
| **Highest level of education** | | | | |
| None | 10(2.5) | 7(1.8) | 21(5.3) | **<0.001** |
| Primary | 198(49.5) | 146(36.4) | 254(63.5) |  |
| Secondary/ Tertiary | 192(48.0) | 246(61.7) | 125(31.3) |  |
| **Occupation** | | | | |
| Outdoor/Animal-Related^a^ | 264(66.0) | 237(59.4) | 272(68.0) | **<0.001** |
| Formal/Structured^b^ | 85(21.3) | 82(20.6) | 22(5.5) |  |
| Informal/Unstructured^c^ | 51(12.8) | 80(20.1) | 106(26.5) |  |
| **Livestock Ownership** | | | | |
| **Own goats** | | | | |
| Yes | 138(34.5) | 103(25.8) | 32(8.0) | **<0.001** |
| No | 262(65.5) | 296(74.2) | 368(92.0) |  |
| **Own cattle** | | | | |
| Yes | 58(14.5) | 64(16.0) | 31(7.8) | **0.001** |
| No | 342(85.5) | 335(84.0) | 369(92.3) |  |
| **Own pigs** | | | | |
| Yes | 209(52.3) | 173(43.4) | 49(12.3) | **<0.001** |
| No | 191(47.8) | 226(56.6) | 351(87.8) |  |
| **Bednet use** | | | | |
| Does not use | 33(8.3) | 24(6.0) | 69(17.3) | **<0.001** |
| Uses bed-net | 367(91.8) | 375(94.0) | 331(82.8) |  |
| **Cultivable land** | | | | |
| No land | 18(4.5) | 64(16.0) | 271(67.8) | **<0.001** |
| Owns cultivable land | 382(95.5) | 335(84.0) | 129(32.3) |  |

* *p-values are from chi-square or Fisher’s exact test as appropriate, comparing the distribution of each variable across the three community types (Agrarian, Trading, and Fishing).*

*a; Outdoor/Animal-Related livelihoods; primarily involving agriculture, fishing, or animal handling.*

*b; Formal/Structured; with stable income and defined roles, such as teaching, civil service, or students.*

*c; Informal/Unstructured; Small-scale work with irregular income, such as gambling, sex work, or domestic help.*

***Note****: Some demographic parameters (sex, age, education, and livestock ownership) were previously described in a CCHFV study* (*1*) *using the same RCCS cohort and are included here for context in the RVFV analysis.*

1. E. A. Mihalakakos, V. Ssempijja, R. M. Ribeiro, C. Molina-Paris, G. Katushabe, J. Nalwadda, J. Omooja, D. K. Byarugaba, K. Rosenke, S. J. Reynolds, M. K. Grabowski, R. M. Galiwango, R. Ssekubugu, H. Feldmann, D. W. Hawman, Longitudinal seroprevalence of Crimean-Congo hemorrhagic fever virus in Southern Uganda. *Emerg. Microbes Infect.* **14**, 2465315 (2025).
